# The stochastic nonlocal Cahn–Hilliard equation with regular potential and multiplicative noise

**DOI:** 10.1007/s40072-026-00430-2

**Published:** 2026-05-18

**Authors:** Andrea Di Primio, Christoph Hurm

**Affiliations:** 1 Classe di Scienze, Scuola Normale Superiore, Pisa, 56126 Italy; 2 Fakultät für Mathematik, Universität Regensburg, Regensburg, 93040 Germany

**Keywords:** Nonlocal Cahn–Hilliard system, Stochastic two-phase flow, Martingale solutions, Pathwise uniqueness, Nonlocal-to-local asymptotics, Primary 35R60, 60H15, 76T06

## Abstract

In this work, we deal with the stochastic counterpart of the nonlocal Cahn–Hilliard equation with regular potential in a smooth bounded one-, two- or three-dimensional domain. The problem is endowed with homogeneous Neumann boundary conditions and random initial data. Furthermore, the system is driven by cylindrical noise of multiplicative type. For the resulting system, we are able to show the existence of probabilistically-weak (or martingale) solutions in two and three dimensions, that are unique and probabilistically-strong under suitable assumptions on the stochastic diffusion. Moreover, we investigate the nonlocal-to-local asymptotics toward solutions of the local stochastic Cahn–Hilliard equations, establishing, under regularity conditions, a precise rate of convergence as well.

## Introduction

Phase separation is a peculiar physical phenomenon arising in certain multicompoment systems when subject to thermodynamical stress. A canonical example of this behavior is given by a binary homogeneous mixture that is rapidly quenched under a critical temperature. Under these conditions, separation dynamics may occur: the two components of the system reorganize through a pattern formation process, often leading, at equilibrium, to the formation of subregions that are rich in a single phase. The dynamics of phase separation generally unfold through two successive regimes, each characterized by different temporal and spatial scales. The initial stage, called spinodal decomposition, is the driver of the early stages of phase separation: microstructures form at small spatial scales as a result of thermodynamical instabilities. On longer time scales, the system enters the coarsening stage: macroscopic patterns emerge as multiple microstructures merge into larger regions, eventually towards some equilibrium state. From the modeling standpoint, the first complete description of phase separation dates back to the pioneering contributions of J. W. Cahn and J. E. Hilliard, in the context of metallic binary alloys (see, e.g., [4]). Precisely, for $d \in \{1,2,3\}$, let $\mathcal {O}\subset \mathbb {R}^d$ be a bounded smooth domain and fix a time horizon T>0. The well-known Cahn–Hilliard equation for a binary system reads

1.1 $$\begin{aligned} {\left\{ \begin{array}{ll} \partial _t \varphi - \Delta \mu =0 &  \quad \text {in } (0,T) \times \mathcal {O}, \\ \mu = -\Delta \varphi + F'(\varphi )&  \quad \text {in } (0,T) \times \mathcal {O}, \\ \partial _{{\boldsymbol{n}}} \mu = \partial _{{\boldsymbol{n}}} \varphi = 0 &  \quad \text {on } (0,T) \times \partial \mathcal {O}, \\ \varphi (0) = \varphi _0 &  \quad \text {in } \mathcal {O}, \end{array}\right. } \end{aligned}$$

where φ denotes the relative concentration difference between the two components, μ denotes the chemical potential and *F* represents a potential energy density for the system. The choice of the potential *F* plays a crucial role when evaluating how close (1.1) is to an actual physical model. Indeed, since φ denotes a difference of two concentrations, it is natural to expect the physically meaningful constraint

1.2 $$\begin{aligned} \varphi (x,t) \in [-1,1] \qquad \forall \, (x,t) \in \mathcal {O}\times [0,T), \end{aligned}$$

so that, at a fixed time t≥0, the level sets $\{\varphi (\cdot ,\, t) = 1\}$ and $\{\varphi (\cdot ,\, t) = -1\}$ represent the subregions filled with a single component of the system, while the set $\{\varphi (\cdot ,\, t) \in (-1,1)\}$ denotes the so-called diffuse interface, i.e., a layer of positive thickness where some mixing takes place. However, it is a priori unclear how (1.1) should ensure this property. In this regard, choosing a function *F* whose derivatives blow up at ±1 can yield to a physically consistent description of phase separation. The most appropriate choice of the potential *F* can be derived from thermodynamical arguments and is the so-called Flory–Huggins (or singular) double-well potential (see [21, 31]), i.e.,

$$ F_{\text {log}}:[-1,1] \rightarrow \mathbb {R}, \qquad F_{\text {log}}(s) = \dfrac{\theta }{2}\left[ (1+s)\log (1+s)+(1-s)\log (1-s)\right] - \dfrac{\theta _0}{2}s^2, $$

extended by continuity at the endpoints. Here, θ denotes the absolute temperature of the system (which is assumed to be constant) and $\theta _0$, that satisfies $0< \theta < \theta _0$, is the critical temperature under which phase separation takes place. Nonetheless, from both the modeling and the analytical viewpoints, alternative choices of *F* are also of significant interest. A second, widely employed example for the potential *F* is actually a regular approximation of $F_{\text {log}}$, for instance,

$$ F_{\text {reg}}: \mathbb {R} \rightarrow \mathbb {R}, \qquad F_{\text {reg}}(s) = \dfrac{(s^2-1)^2}{4}, $$

which retains a double-well structure, but shifts the two global minimum points at s=±1 and is globally smooth over $\mathbb {R}$ (i.e., no derivative of $F_{\text {reg}}$ exhibits singularities). Although regular potentials as $F_{\text {reg}}$ do not strictly enforce the physical constraint (1.2), they provide a flexible and robust modeling framework. For instance, they allow for a clearer mathematical analysis and facilitate numerical approximations. Moreover, the investigation of the Cahn–Hilliard system (1.1) with regular potentials is a necessary preliminary step to understand the singular case, while also retaining independent interest in several models (see, for instance, [16, Section 1] for some remarks on the topic). Regardless of the specific form of the potential energy density *F*, formally, the Cahn–Hilliard system (1.1) has a $H^{-1}$-gradient flow structure: indeed, it can be derived starting from the energy functional

1.3 $$\begin{aligned} \mathcal {E}_\text {L}(\varphi ) = \int _\mathcal {O}\dfrac{1}{2}|\nabla \varphi (x)|^2 + F(\varphi (x)) \, {\mathrm d}x \end{aligned}$$

and computing the chemical potential μ as its first variational derivative. In this work, we shall actually be interested in a variant of system (1.1), which accounts for long-range interactions. Indeed, the energy functional (1.3) only takes into consideration contributions due to local interactions (so that system (1.1) is also named the local Cahn–Hilliard equation), penalizing the presence of spatial variations across the interface. At the end of the last century, G. Giacomin and J. L. Lebowitz (see [26, 27] and [28]) addressed the fundamental problem of deriving a model of phase separation through a stochastic hydrodynamic limit. The resulting model is similar to (1.1) in structure, but captures the energy contributions due to nonlocal interactions. Retaining the notation in (1.1), the so-called nonlocal Cahn–Hilliard equation reads

1.4 $$\begin{aligned} {\left\{ \begin{array}{ll} \partial _t \varphi - \Delta \mu =0 &  \quad \text {in } (0,T) \times \mathcal {O}, \\ \mu = (K*1)\varphi -K * \varphi + F'(\varphi )&  \quad \text {in } (0,T) \times \mathcal {O}, \\ \partial _{{\boldsymbol{n}}} \mu = 0 &  \quad \text {on } (0,T) \times \partial \mathcal {O}, \\ \varphi (0) = \varphi _0 &  \quad \text {in } \mathcal {O}, \end{array}\right. } \end{aligned}$$

where $K: \mathbb {R}^d \rightarrow \mathbb {R}$ is a suitable interaction kernel and $F: \mathbb {R} \rightarrow \mathbb {R}$ is still a potential energy density function. The peculiarity of this model is that an analogous derivation for the local equation (1.1) is, to our knowledge, still not available to this day. Of course, the energy functional (1.5) is not the appropriate one when considering the nonlocal variant (1.4): indeed, the gradient squared term has to be replaced by a suitable nonlocal counterpart, that is

1.5 $$\begin{aligned} \mathcal {E}_\text {NL}(\varphi ) = \dfrac{1}{4}\int _\mathcal {O}\int _\mathcal {O}K(x-y)(\varphi (x)-\varphi (y))^2 \, {\mathrm d}x \, {\mathrm d}y + \int _\mathcal {O}F(\varphi (x)) \, {\mathrm d}x. \end{aligned}$$

Equation (1.4) has been studied in a number of recent contributions, both as a standalone problem or coupled with other equations from mathematical physics (e.g., the Navier–Stokes equations), refer for instance to [22–25] and the references therein.

On the modeling side, however, it should be noted that the purely deterministic description of phase separation (either local or nonlocal) fails in capturing thermodynamical fluctuations or environmental noise, that are characterized by unpredictable oscillations at the microscopic level and play are particularly relevant during spinodal decomoposition. The first effort towards a refinement of the local Cahn–Hilliard equation in this direction was proposed in 1970 (see [5]), where a stochastic version of equation (1.1) was introduced (known as the stochastic Cahn–Hilliard equation or the Cahn–Hilliard–Cook equation). Since then, this stochastic counterpart has been analyzed in a number of works (see, for instance, [7, 11, 12, 18, 29] and, more recently, [15, 36–38], see also [14] for a coupled model). All of the previously mentioned works, however, deal with the local version of the model. Indeed, the only references about nonlocal diffuse interface models concern more complicated models including a drift, either under additive noise (see [6]), or without a direct stochastic forcing (see [13], for instance). In this work, we shall consider the stochastic version of the nonlocal Cahn–Hilliard equation, that is,

1.6 $$\begin{aligned} {\left\{ \begin{array}{ll} {\mathrm d}\varphi - \Delta \mu \, {\mathrm d}t = G(\varphi ) \, {\mathrm d}W &  \quad \text {in } (0,T) \times \mathcal {O}, \\ \mu = (K*1)\varphi - K * \varphi + F'(\varphi )&  \quad \text {in } (0,T) \times \mathcal {O}, \\ \partial _{{\boldsymbol{n}}} \mu = 0 &  \quad \text {on } (0,T) \times \partial \mathcal {O}, \\ \varphi (0) = \varphi _0 &  \quad \text {in } \mathcal {O}, \end{array}\right. } \end{aligned}$$

where $F: \mathbb {R}\rightarrow \mathbb {R}$ is a given regular potential density function, $K: \mathbb {R}^d \rightarrow \mathbb {R}$ is a given interaction kernel and *G* is a suitable stochastically integrable diffusion process of multiplicative type. After the necessary preliminaries, we employ a Yosida regularization scheme and a stochastic compactness argument to show the existence of martingale solutions. If the noise enables conservation of mass (see also [15] for further observations on the topic), then, by establishing pathwise uniqueness, we are able to show that even probabilistically-strong solutions exist. Finally, we estalish the nonlocal-to-local asymptotics of the problem, showing that along a suitable sequence of nonlocal kernels, solutions to the nonlocal problem (1.6) converge to solutions of the corresponding local problem. The content of this work is structured as follows. In Section 2, we introduce the notation used throughout the work and state the main results. Sections 3 and 4 are devoted to the proof of existence of a martingale solution and of a probabilistically-strong solution, respectively. Finally, in Sections 5 and 6 we investigate the nonlocal-to-local asymptotics of the model.

## Preliminaries and main results

### Functional setting and notation

The notation and both the analytical and stochastic frameworks that are needed to analyze the problem are illustrated in the following paragraphs.

*Stochastic setting.* Let $(\Omega ,\mathscr {F},(\mathscr {F}_t)_{t\in [0,T]},\mathbb {P})$ be any filtered probability space satisfying the usual conditions (namely the filtration is saturated and right-continuous), with T>0 being a prescribed final time. Let us denote the progressive σ-algebra on $\Omega \times [0,T]$ by $\mathscr {P}$. Let further $(E,\, \mathcal {M},\, m)$ be any probability space. If $X: \Omega \rightarrow E$ and $Y: \Omega \rightarrow E$ are two random variables, then the notation

$$ X {\mathop {=}\limits ^{\mathcal {L}}}Y $$

denotes the identity of their laws. When *E* is also a Banach space (in these cases, we always understand that $\mathcal {M}$ is the Borel σ-algebra of *E* unless otherwise stated), for any positive real quantity p≥1 the symbol $L^p(\Omega ; E)$ denotes the set of strongly measurable *E*-valued random variables on Ω with finite moments up to order *p*. If the space *E* is allowed to depend on time, as in classical Bochner spaces, we may denote the resulting space with $L^p_\mathscr {P}(\Omega ; E)$ to stress that measurability is intended with respect to the progressive σ-algebra $\mathscr {P}$. Throughout the work, the symbol *W* denotes a cylindrical Wiener process on some separable, fixed Hilbert space *U*. We shall denote by $\{u_j\}_{j\in \mathbb {N}} \subset U$ a fixed arbitrary orthonormal system for *U*. In order to avoid any ambiguities, we precise the rigorous interpretation of the stochastic perturbation appearing in (1.6) as an Itô integral. Being a cylindrical process on *U*, the Wiener process *W* admits the representation

2.1 $$\begin{aligned} W = \sum _{k=0}^{+\infty } \beta _k u_k, \end{aligned}$$

where $\{\beta _k\}_{k \in \mathbb {N}}$ is a family of real and independent Brownian motions. However, the series (2.1) can not be expected to always converge in *U*. In general, there exists some larger Hilbert space $U_0$ such that $U \hookrightarrow U_0$ with Hilbert-Schmidt embedding ι and such that we can identify *W* as a $Q^0$-Wiener process on $U_0$, for some trace-class operator $Q^0$ (see [33, Subsection 2.5.1]). Actually, it holds that $Q^0 = \iota \circ \iota ^*$. In the following, we may implicitly assume this extension by simply saying that *W* is a cylindrical process on *U*. This holds also for stochastic integration with respect to *W*. Indeed, the symbol

$$ \int _0^\cdot B(s)\,{\mathrm d}W(s) := \int _0^\cdot B(s) \circ \iota ^{-1}(s)\,{\mathrm d}W(s), $$

for every process $B \in L^2_\mathscr {P}(\Omega ;L^2(0,T;\mathscr {L}^2(U,K)))$, where *K* is any real Hilbert space and the space $\mathscr {L}^2(U,K)$ is defined later. It is well known that such a definition is well posed and does not depend on the choice of $U_0$ or ι (see [33, Subsection 2.5.2]).

*Analytical setting*. Let *E* be any Banach space. The symbol $\boldsymbol{E}$ denotes the product space $E^d$ (or even $E^{d\times d}$, if no ambiguities arise), while its topological dual is denoted by $E^*$ and the corresponding duality pairing is denoted by $\left\langle \cdot ,\cdot \right\rangle _{E^*,E}$. If *E* is a Hilbert space, then the scalar product of *E* is denoted by $(\cdot ,\cdot )_E$. Given two Banach spaces $E_1$ and $E_2$ and an extended real number $s \in [1,+\infty ]$, the symbol $L^s(E_1; E_2)$ indicates the usual spaces of strongly measurable, Bochner-integrable functions defined on the Banach space $E_1$ and with values in the Banach spaces $E_2$. If $E_2$ is omitted, it is understood that $E_2 = \mathbb {R}$. When $E_1$ and $E_2$ are also Hilbert spaces, we denote the space of Hilbert-Schmidt operators from $E_1$ to $E_2$ by the symbol $\mathscr {L}^2(E_1,\,E_2)$, and we endow it with its canonical norm $\left\| \,\cdot \,\right\| _{\mathscr {L}^2(E_1,\,E_2)}$. For all $s\in (1,+\infty )$ and for every separable and reflexive Banach space *E*, we also define

$$\begin{aligned}&L^s_w(\Omega ; L^\infty (0,T; E^*)):= \left\{ v:\Omega \rightarrow L^\infty (0,T; E^*) \right. \\&\left. \quad \text { weakly*-measurable and } \left\| v\right\| _{L^\infty (0,T; E^*)}\in L^s(\Omega ) \right\} \,, \end{aligned}$$

which yields by [17, Theorem 8.20.3] the identification

$$ L^s_w(\Omega ; L^\infty (0,T; E^*))= \left( L^{\frac{s}{s-1}}(\Omega ; L^1(0,T; E))\right) ^*\,. $$

Fixed a smooth and bounded domain $\mathcal {O}\subset \mathbb {R}^d$, with $d \in \{1,2,3\}$, we denote by $W^{s,p}(\mathcal {O})$ the classical Sobolev spaces of order $s \in \mathbb {R}$ and $p \in [1,+\infty ]$, and we denote by $\left\| \,\cdot \,\right\| _{W^{s,p}(\mathcal {O}))}$ their canonical norms (with the usual understanding that $W^{0,p}(\mathcal {O}) = L^p(\mathcal {O})$). Accordingly, we define the Hilbert space $H^s(\mathcal {O}):=W^{s,2}(\mathcal {O})$ for all $s\in \mathbb {R}$, endowed with its canonical norm $\left\| \,\cdot \,\right\| _{H^s(\mathcal {O})}$. Moreover, we set

$$ H:=L^2(\mathcal {O})\,, \qquad V:=H^1(\mathcal {O})\, $$

endowed with their standard norms $\left\| \cdot \right\| _H$, $\left\| \cdot \right\| _{V}$, respectively. As usual, we identify the Hilbert space *H* with its dual through the corresponding Riesz isomorphism, so that we have the variational structure

$$ V\hookrightarrow H \hookrightarrow V^*, $$

with dense and compact embeddings (both in the cases d=2 and d=3). Finally, we set the zero-mean spaces

$$ H_0 := \left\{ u \in H : \overline{u} := \dfrac{1}{|\mathcal {O}|}\int _{\mathcal {O}} u \, {\mathrm d}x = 0\right\} , \qquad V_0 :=V \cap H_0. $$

The space $H_0$ is endowed with the structure induced by *H*, hence we still carry over the same notation. Instead, owing to the Poincaré inequality, we set the $H^1$-seminorm structure on $V_0$, namely

$$ ( u,\,v)_{V_0} := (\nabla u, \,\nabla v)_{ {\boldsymbol{H}}}, \qquad \Vert u\Vert _{V_0} := \Vert \nabla u\Vert _ {\boldsymbol{H}}, \qquad u,\,v \in V_0. $$

This generates the zero-mean variational triplet

$$ V_0 \hookrightarrow H_0 \hookrightarrow V_0^*, $$

once again with compact and dense embeddings in two and three dimensions. It is well-known that the operator

$$\begin{aligned} \mathcal {A}: V_0\rightarrow V_0^*,\quad \langle \mathcal {A}u,v\rangle _{V_0} := (\nabla u, \,\nabla v)_{ {\boldsymbol{H}}}\quad \text { for all }v\in V_0 \end{aligned}$$

is a linear isomorphism. The operator $\mathcal {A}$ is then called the negative Neumann Laplace operator. We denote its inverse, which is a bounded linear operator, by

$$\begin{aligned} \mathcal {N} := \mathcal {A}^{-1}: V_0^*\rightarrow V_0. \end{aligned}$$

### Structural assumptions

The following assumptions will be needed throughout the whole work. Here and in the following, we refer to the stochastic nonlocal Cahn–Hilliard problem (1.6).

**(A1)**: The nonlocal kernel $K \in W^{1,1}_{\text {loc}}(\mathbb {R}^d)$ satisfies K(x)=K(-x) for all $x \in \mathbb {R}^d$. Throughout the entire work, we stress once and for all that the norms of *K* shall be computed over a ball $B_R \supset {\overline{\mathcal {O}}}$ with a sufficiently large radius R>0. Moreover, we assume that
  $$\begin{aligned} a(x) := (K*1)(x) = \int _\mathcal {O}K(x-y) \, {\mathrm d}y \ge 0 \end{aligned}$$
  for almost every $x\in \mathcal {O}$.
**(A2)**: The potential $F:\mathbb {R}\rightarrow \mathbb {R}$ is such that $F \in C^2(\mathbb {R})$ and satisfies the following properties:
  (i): it holds $F^{\prime }\left(0\right)=0$ and F(r)≥0 for all $$r \in \mathbb {R}$$,
  (ii): there exists a constant $C_F > 0$ such that
    $$ |F'(r)|+|F''(r)| \le C_F(1 + F(r)) $$
    for all $$r \in \mathbb {R}$$,
  (iii): the second derivative $F^{\prime \prime }$ is bounded from below and there exists a constant $$C_0>0$$ such that
    $$\begin{aligned} {\inf _{s \in \mathbb {R}} F^{\prime \prime }(s)+ \mathop {\textrm{ess}\,\textrm{inf}}\limits _{{x \in \mathcal {O}}} \, a(x)} \ge C_0. \end{aligned}$$
**(A3)**: The measurable operator $G: H \rightarrow \mathscr {L}^2(U,H)$ satisfies the following properties:
  (i): there exists a constant $L_G > 0$ such that
    $$ \Vert G(\psi _1)-G(\psi _2)\Vert _{\mathscr {L}^2(U,H)} \le L_G\Vert \psi _1-\psi _2\Vert _H \qquad \forall \, \psi _1,\,\psi _2 \in H, $$
    i.e., *G* is $L_G$-Lipschitz-continuous;
  (ii): there exists a constant, that we still denote with $L_G > 0$ without loss of generality, such that
    $$ \sum _{k=0}^{+\infty } \Vert G(\psi )[u_k]\Vert _{L^\infty (\mathcal {O})}^2 \le L_G $$
    for all $$\psi \in H$$;
  (iii): there exists a constant, that we still denote with $L_G > 0$ without loss of generality, such that
    $$ \Vert G(\psi )\Vert _{\mathscr {L}^2(U,V)} \le L_G\left( 1 + \Vert \psi \Vert _V\right) \qquad \forall \, \psi \in V, $$
    i.e., *G* is linearly bounded in *V*.

#### Remark 2.1

Let us briefly comment further on the previous assumptions. First of all, observe that for a sufficiently large R>0, so that ${\mathcal {O}- \mathcal {O}:= \left\{ x-y: x \in \mathcal {O}, \, y \in \mathcal {O}\right\} } \subset B_R$, we have

$$ { |a(x)| =\left| \int _{\mathcal {O}} K(x-y) \, {\mathrm d}y \right| = \left| \int _{x-\mathcal {O}} K(z) \, {\mathrm d}z \right| \le \Vert K\Vert _{L^1(\mathcal {O}- \mathcal {O})} \le \Vert K\Vert _{L^1(B_R)} } $$

and therefore $a \in L^\infty (\mathcal {O})$, owing to Assumption (**A1**). Assumptions (**A2**)-(i) and (ii) allow for a wide class of regular potentials encompassing the usual quartic $F_{\text {reg}}$, as seen in Section 1, but also including functions of exponential growth. However, Assumption (**A2**)-(iii) poses a compatibility condition between the nonlocal kernel *K* and the potential *F*, in terms of a coercivity condition. Under Assumption (**A1**), this is trivially satisfied whenever *F* is strongly convex, but is in general crucial in the nonlocal case, as no control on *V*-norms is otherwise possible. Indeed, this hypothesis has two important consequences. First of all, we clearly have

$$ F''(s) + a(x) \ge \inf _{s \in \mathbb {R}} F''(s) + \mathop {\textrm{ess}\,\textrm{inf}}\limits _{{x \in \mathcal {O}}} \, a(x) \ge C_0 $$

for all $s \in \mathbb {R}$ and almost all $x \in \mathcal {O}$. Observe that, in general, the above may hold even if *F* is not convex. Secondly, it is clear that *F* can be written as

$$ F(s) = \Psi (s) + R(s) \qquad s \in \mathbb {R}, $$

where Ψ is a strongly convex function and *R* is a concave second-order perturbation. Letting indeed

$$ \inf _{s \in \mathbb {R}} F''(s) = -\alpha , $$

and assuming $\alpha \ge 0$ (otherwise one can take F=Ψ and R≡0) one can set

$$ R(s) = -\dfrac{\gamma }{2} s^2 $$

for some $\gamma > \alpha$ and then set Ψ(s)=F(s)-R(s) for all $s \in \mathbb {R}$. Observe further that the function *a* defined in Assumption (**A1**), in order to be compatible with the potential *F*, must satisfy

$$ a(x) \ge C_0 + \alpha $$

for almost all $x \in \mathcal {O}$. Finally, Assumption (**A3**) is often assumed in the context of stochastic diffuse interface models (see, for instance, [14, 15, 19, 36]), also in the singular case. In most instances, the noise is assumed to be of superposition type, i.e., for every $k \in \mathbb {N}$ and $\psi \in H$

$$ G(\psi )[u_k] = g_k(\psi ) $$

with $\{g_k\}_{k \in \mathbb {N}} \subset W^{1,\infty }(\mathbb {R})$ and such that

$$ \sum _{k=0}^{+\infty }\Vert g_k\Vert _{W^{1,\infty }(\mathbb {R})}^2 \le C. $$

When pathwise mass conservation is required, it is also necessary to require that the range of *G* lies in $\mathscr {L}^2(U,H_0)$ instead, so for example one can define

$$ G(\psi )[u_k] = g_k(\psi ) - \overline{g_k(\psi )} $$

for every $k \in \mathbb {N}$ and $\psi \in H$, or express $G(\psi )[u_k]$ as the divergence of suitably regular vector fields (see also [15] for further details).

### Main results

Before stating the main results of the work, let us first precise the concept of martingale and probabilistically-strong solutions for system (1.6), starting with the former.

#### Definition 2.2

Let p≥2 and let $\varphi _0$ satisfy

$$ \varphi _0 \in L^p(\Omega , \mathscr {F}_0; H), \qquad F(\varphi _0) \in L^{\frac{p}{2}}(\Omega , \mathscr {F}_0; L^1(\mathcal {O})). $$

A martingale solution to the stochastic nonlocal Cahn–Hilliard problem (1.6) with respect to the initial datum $\varphi _0$ is a family

$$ \left\{ \left( \widehat{\Omega }, \widehat{\mathscr {F}}, (\widehat{\mathscr {F}})_{t \in [0,T]}, \widehat{\mathbb {P}}\right) , \widehat{W}, \widehat{\varphi }\right\} $$

such that $\left( \widehat{\Omega }, \widehat{\mathscr {F}}, (\widehat{\mathscr {F}})_{t \in [0,T]}, \widehat{\mathbb {P}}\right)$ is a filtered probability space satisfying the usual conditions, $\widehat{W}$ is a cylindrical Wiener process on a separable Hilbert space *U*, and $\widehat{\varphi }$ is an $\widehat{\mathscr {F}}_t$-adapted process such that

$$\begin{aligned}&\widehat{\varphi }\in L^p_w(\widehat{\Omega }; L^\infty (0,T;H)) \cap L^p_\mathscr {P}(\widehat{\Omega };L^2(0,T;V)) \\&\widehat{\mu }:=a\widehat{\varphi }-K*\widehat{\varphi }+F'(\widehat{\varphi }) \in L^{\frac{p}{2}}_\mathscr {P}(\widehat{\Omega }; L^2(0,T; V))\,,\\&\widehat{\varphi }(0) {\mathop {=}\limits ^{\mathcal {L}}}\varphi _0, \end{aligned}$$

and

$$\begin{aligned} (\widehat{\varphi }(t),\psi )_H + \int _0^t\!\int _\mathcal {O}\nabla \widehat{\mu }(s)\cdot \nabla \psi \,{{\mathrm d}x\,}{\mathrm d}s = ( \widehat{\varphi }(0),\,\psi )_{H} + \left( \int _0^t G( \widehat{\varphi }(s))\,{\mathrm d}\widehat{W}(s),\, \psi \right) _{H} \end{aligned}$$

for all t∈[0,T], for every $\psi \in V$ and $\widehat{\mathbb {P}}$-almost surely.

#### Definition 2.3

Let p≥2 and let $\varphi _0$ satisfy

$$ \varphi _0 \in L^p(\Omega , \mathscr {F}_0; H), \qquad F(\varphi _0) \in L^{\frac{p}{2}}(\Omega , \mathscr {F}_0; L^1(\mathcal {O})). $$

Given a stochastic basis

$$ \left( \Omega ,\, \mathscr {F},\,(\mathscr {F}_t)_{t \in [0,T]},\, \mathbb {P}\right) $$

satisfying the usual conditions and a cylindrical Wiener process *W* on a separable Hilbert space *U*, a probabilistically-strong solution to the stochastic nonlocal Cahn–Hilliard problem (1.6) with respect to the initial datum $\varphi _0$ is an $\mathscr {F}_t$-adapted process φ such that

$$\begin{aligned}&\varphi \in L^p_w(\Omega ; L^\infty (0,T;H)) \cap L^p_\mathscr {P}(\Omega ;L^2(0,T;V)) \\&\mu :=a\varphi -K*\varphi +F'(\varphi ) \in L^{\frac{p}{2}}_\mathscr {P}(\Omega ; L^2(0,T; V))\,,\\&\varphi (0) = \varphi _0, \end{aligned}$$

and

$$\begin{aligned} (\varphi (t),\psi )_H + \int _0^t\!\int _\mathcal {O}\nabla \mu (s)\cdot \nabla \psi \,{{\mathrm d}x}\,{\mathrm d}s = ( \varphi (0),\,\psi )_{H} + \left( \int _0^t G( \varphi (s))\,{\mathrm d}W(s),\, \psi \right) _{H} \end{aligned}$$

for all t∈[0,T], for every $\psi \in V$ and $\mathbb {P}$-almost surely.

We are now in a position to state the main results of this work. The first result concerns the existence of martingale solutions and will be proven in Section 3.

#### Theorem 2.4

Let Assumptions (**A1**)-(**A3**) hold. Let p>2 and let $\varphi _0$ satisfy

$$ \varphi _0 \in L^p(\Omega , \mathscr {F}_0; H), \qquad F(\varphi _0) \in L^{\frac{p}{2}}(\Omega , \mathscr {F}_0; L^1(\mathcal {O})). $$

Then, there exists at least one martingale solution to the stochastic nonlocal Cahn–Hilliard equation (1.6).

Under stronger assumptions on the stochastic diffusion *G*, we are also able to prove a continuous dependence estimate on the full paths, yielding pathwise uniqueness of martingale solutions.

(**A4**): Fixed a complete orthonormal system $\{u_k\}_{k \in \mathbb {N}}\subset U$, the stochastic diffusion $G:H\rightarrow \mathscr {L}^2(U, H)$ satisfies
  $$ \overline{G(\psi )[u_k]} = 0 $$
  for all $k \in \mathbb {N}$, and there exists a positive constant, that we still denote with $L_G > 0$, such that
  $$ \Vert G(\psi _1)-G(\psi _2)\Vert _{\mathscr {L}^2(U,V^*)} \le L_G\Vert \psi _1-\psi _2\Vert _{V^*} $$
  for all $\psi _1,\,\psi _2 \in H$, i.e., *G* is $L_G$-Lipschitz-continuous with respect to the $V^*$-topology.

#### Remark 2.5

As we shall better see later, the assumption of Lipschitz continuity in the $V^*$-topology could be replaced by a smallness assumption on the Lipschitz constant with respect to the *H*-topology. Nonetheless, in order to avoid ambiguities in the following, we stick with this more classical assumption.

#### Remark 2.6

Assumption (**A4**) implies that the dynamics of problem is mass-conservative in a pathwise sense, i.e., that any probabilistically-strong solution satisfies

$$ \overline{\varphi (t)} = \overline{\varphi _0} $$

for all t>0, $\mathbb {P}$-almost surely. This follows by taking ψ=1 in the variational formulation of the problem and recovers a key feature of the deterministic version of the system. Moreover, Assumption (**A4**) accounts for the fact that a continuous dependence estimate in the *H*-norm for the Cahn–Hilliard equation (both local and nonlocal) is generally out of reach due to the nonlinear potential term.

#### Theorem 2.7

Let Assumptions (**A1**)-(**A4**) hold. Let p>2 and, for $i \in \{1, 2\}$, let $\varphi _{0i}$ comply with Theorem 2.4. Assume that $\widehat{\varphi }_1$ and $\widehat{\varphi }_2$ are defined on the same filtered probability space $(\widehat{\Omega }, \widehat{\mathscr {F}}, (\widehat{\mathscr {F}}_t)_{t \in [0,T]},\widehat{\mathbb {P}})$ and solve (1.6) driven by the same $\widehat{\mathscr {F}}_t$-adapted Wiener process $\widehat{W}$ and with respect to the initial data $\varphi _{01}$ and $\varphi _{02}$, respectively. If $\overline{\varphi _{01}} = \overline{\varphi _{02}}$ $\mathbb {P}$-almost surely, then, the continuous dependence estimate

$$\begin{aligned} \Vert \widehat{\varphi }_1-\widehat{\varphi }_2\Vert _{L^p(\widehat{\Omega }; L^\infty (0,T;V^*))} + \Vert \widehat{\varphi }_1-\widehat{\varphi }_2\Vert _{L^p(\widehat{\Omega }; L^2(0,T;H))} \le C\Vert \widehat{\varphi }_{01}-\widehat{\varphi }_{02}\Vert _{L^p(\widehat{\Omega };V^*)} \end{aligned}$$

holds, where $\widehat{\varphi }_{0i}:= \widehat{\varphi }_{i}(0) {\mathop {=}\limits ^{\mathcal {L}}}\varphi _{0i}$ as *H*-valued random variables for $i \in \{1,2\}$. In particular, the martingale solution to (1.6) is pathwise unique.

In any case, as a consequence of the previous result and the Yamada–Watanabe theorem (see, for instance, [33, Theorem E.0.8]), we have the straightforward corollary

#### Corollary 2.8

Let Assumptions (**A1**)-(**A4**) hold. Let p>2 and let $\varphi _0$ satisfy

$$ \varphi _0 \in L^p(\Omega , \mathscr {F}_0; H), \qquad F(\varphi _0) \in L^{\frac{p}{2}}(\Omega , \mathscr {F}_0; L^1(\mathcal {O})). $$

Then, there exists a unique probabilistically-strong solution to the stochastic nonlocal Cahn–Hilliard equation (1.6).

The proofs of Theorem 2.7 and Corollary 2.8 will be discussed in Section 4. The last results of the work concerns the nonlocal-to-local asymptotics for the stochastic Cahn–Hilliard equation, i.e., establishing whether and in which sense the solutions of the nonlocal equations converge to the ones of the local problem, that reads

2.2 $$\begin{aligned} {\left\{ \begin{array}{ll} {\mathrm d}\varphi - \Delta \mu \, {\mathrm d}t = G(\varphi ) \, {\mathrm d}W &  \quad \text {in } (0,T) \times \mathcal {O}, \\ \mu = -\Delta \varphi + F'(\varphi )&  \quad \text {in } (0,T) \times \mathcal {O}, \\ \partial _{{\boldsymbol{n}}} \mu = \partial _{\boldsymbol{n}}\varphi =0 &  \quad \text {on } (0,T) \times \partial \mathcal {O}, \\ \varphi (0) = \varphi _0 &  \quad \text {in } \mathcal {O}. \end{array}\right. } \end{aligned}$$

More precisely, we shall make use of a specific sequence of nonlocal kernels. In the following, we denote by $\{\rho _\varepsilon : \mathbb {R} \rightarrow [0,+\infty )\}_{\varepsilon >0}$ a family of nonnegative mollifiers satisfying the following properties:

(i) the function $\rho _\varepsilon$ is integrable for all ε>0;
(ii) the function $\rho _\varepsilon$ is even, i.e., $\rho _\varepsilon (r) = \rho _\varepsilon (-r)$ for all $r \in \mathbb {R}$ and ε>0;
(iii) the relation
  $$ \int _{0}^{+\infty }\rho _\varepsilon (r)\,r^{n-1}\,\text {d}r = \frac{2}{C_n} $$
  holds for all integers n≥2, where
  $$ C_n := \int _{\mathbb {S}^{n-1}}|e_1\cdot \sigma |^2\,\text {d}\mathcal {H}^{n-1}(\sigma ), $$
  with $e_1$ being the first element of the canonical basis in $\mathbb {R}^{n}$, $\mathbb {S}^{n-1}$ denoting the unit sphere of $\mathbb {R}^n$ and $\mathcal {H}^{n-1}$ being the (n-1)-dimensional Hausdorff measure;
(iv) it holds
  $$ \lim _{\varepsilon \rightarrow 0^+}\int _{\delta }^\infty \rho _\varepsilon (r)\,r^{n-1}\,\text {d}r = 0 $$
  for all δ>0.

The nonlocal kernels are of the following form.

(**A5**): For any ε>0, we define a nonlocal kernel $K_\varepsilon : \mathbb {R}^n\rightarrow [0,+\infty )$ by
  $$ K_\varepsilon (x) = \frac{\rho _\varepsilon (x)}{|x|^2} $$
  for any $$x \in \mathbb {R}^n$$.

#### Remark 2.9

Assumption (**A5**) is not new in the context of nonlocal-to-local asymptotics for the Cahn–Hilliard equation: see for instance [1, 9, 10] for further motivation. Observe that by [9, Lemma 3.1], we have $K_\varepsilon \in W^{1,1}(\mathbb {R}^n)$.

For the sake of convenience, for any ε>0 and any sufficiently regular function $u:\mathcal {O}\rightarrow \mathbb {R}$, we define the linear nonlocal operator $\mathcal {L}_\varepsilon u$ as

2.3 $$\begin{aligned} \mathcal {L}_\varepsilon u(x) := \int _\mathcal {O}K_\varepsilon (x-y)(u(x)-u(y)) \, {\mathrm d}y \end{aligned}$$

for almost all $x\in \mathcal {O}$. In the limit $\varepsilon \rightarrow 0^+$, it is well-known that the nonlocal operator as in (2.3) converges to the negative Laplacian, cf., e.g., [1]. In this perspective, we analyze the nonlocal problems

2.4 $$\begin{aligned} {\left\{ \begin{array}{ll} {\mathrm d}\varphi _\varepsilon - \Delta \mu _\varepsilon \, {\mathrm d}t = G(\varphi _\varepsilon ) \, {\mathrm d}W &  \quad \text {in } (0,T) \times \mathcal {O}, \\ \mu _\varepsilon = \mathcal {L}_\varepsilon \varphi _\varepsilon + F'(\varphi _\varepsilon )&  \quad \text {in } (0,T) \times \mathcal {O}, \\ \partial _{{\boldsymbol{n}}} \mu _\varepsilon = 0 &  \quad \text {on } (0,T) \times \partial \mathcal {O}, \\ \varphi _\varepsilon (0) = \varphi _{0,\varepsilon } &  \quad \text {in } \mathcal {O}. \end{array}\right. } \end{aligned}$$

Since $K_\varepsilon$ is nonnegative for all ε>0, it is immediate to check that $K_\varepsilon$ satisfies Assumption (**A1**) for all ε>0. Therefore, under Assumptions (**A1**)-(**A3**) and (**A5**), we infer from Theorem 2.4 that (2.4) admits at least one martingale solution. On the remaining Assumption (**A4**), we observe the following:

1. under Assumption (**A4**), Corollary 2.8 gives unique probabilistically-strong solutions for the nonlocal problems (2.4). The same actually holds for the local problem (2.2) as well, as addressed in [37]. In this setting, all the needed solutions can be framed with respect to the same stochastic basis. In particular, it makes sense to compute differences between solutions and estimate a rate of nonlocal-to-local convergence, extending the work previously done in [1];
2. if we do not assume Assumption (**A4**), we lack uniqueness both on the nonlocal and on the local problems, and we can only work with martingale solutions. A meaningful convergence result can then only be achieved in law and for subsequences by means of a compactness argument similar to the one that is necessary to prove existence of martingale solutions.

Therefore, in order to give a precise nonlocal-to-local convergence result, Assumption (**A4**) can not be dropped. In general, we expect that the second case could be improved if a uniqueness result is available at least for the limit problem, for instance by adopting the technique introduced by Gyöngy and Krylov (see [30, Lemma 1.1], see also [15] for an application in the context of diffuse interface models).

#### Remark 2.10

Actually, it turns out that the structure of the kernels $K_\varepsilon$ would in principle enable some relaxation of Assumption (**A4**), as the Lipschitz continuity in the $V^*$-topology turns out not to be necessary to investigate the nonlocal-to-local asymptotics of the system. As will be clear later, this is due to the validity of the so-called nonlocal Ehrling interpolation inequality (see [9, Lemma 3.4]). However, in the present setting Assumption (**A4**) is still needed as-is to grant pathwise uniqueness to the limit local problem (and, coincidentally, to the nonlocal problems as well).

Nonetheless, both the statements of the final results of this work shall need an additional assumption. In light of the results in [1], establishing a rate of convergence strongly depends on some degree of higher-order regularity of the limit solution, which has been proven in [36] only in the case of sub-quartic potentials (in three dimensions). Therefore, we shall need the following assumption.

(**A6**): There exists a positive constant, that without loss of generality we still denote with $C_F >0$, such that
  $$ F''(s) \le C_F(1 + |s|^{q_F}) \qquad \forall \, s \in \mathbb {R}, $$
  where $q_F \ge 2$ if d=2 and $q_F = 2$ if d=3.

Under the additional Assumption (**A6**), it has been shown that solutions of the local problem enjoy more regularity. More precisely, confining ourselves to what is needed in this work, the following lemma holds.

#### Lemma 2.11

( [36, Theorem 2.2] and [37, Theorem 2.10]) Let Assumptions (**A1**)-(**A3**) and (**A6**) hold. Let p≥4 and let $\varphi _0 \in L^p(\Omega , \mathscr {F}_0; V)$ satisfy $F(\varphi _0) \in L^\frac{p}{2}(\Omega , \mathscr {F}_0; L^1(\mathcal {O}))$. Then, the local problem (2.2) admits at least a martingale solution $\{ ( \widehat{\Omega }, \widehat{\mathscr {F}}, (\widehat{\mathscr {F}}_t)_{t \in [0,T]}, \widehat{\mathbb {P}}), \widehat{W}, \widehat{\varphi }\}$ such that

$$ \widehat{\varphi }\in L^\frac{p}{4}(\widehat{\Omega }; L^2(0;T;H^3(\mathcal {O}))). $$

Furthermore, if also Assumption (**A4**) holds, then the solution is unique and probabilistically-strong.

Having Lemma 2.11 at disposal, we can use the results of [1] to derive concrete rates of convergence. This will be done in Section 5. In particular, we show the following result.

#### Theorem 2.12

Let Assumptions (**A1**)-(**A6**) hold. Let p≥4 and, for ε>0, let $\varphi _{0,\varepsilon }$ comply with Theorem 2.4, and let $\varphi _\varepsilon$ denote the probabilistically-strong solution to (2.4) originating from $\varphi _{0,\varepsilon }$. Let further $\varphi _0 \in L^p(\Omega , \mathscr {F}_0; V)$ and $F(\varphi _0) \in L^\frac{p}{2}(\Omega , \mathscr {F}_0;L^1(\mathcal {O}))$ and let φ denote the probabilistically-strong solution to (2.2) originating from $\varphi _{0}$. Assuming that the integral average of $\varphi _{0,\varepsilon }$ does not depend on ε, if

$$ \Vert \varphi _{0,\varepsilon }-\varphi _0\Vert _{L^\frac{p}{4}(\Omega ; V^*)} \le C\sqrt{\varepsilon }, $$

then the estimate

$$\begin{aligned}&\Vert \varphi _\varepsilon -\varphi \Vert _{L^\frac{p}{4}(\Omega ; L^\infty (0,T;V^*))} + \Vert \varphi _\varepsilon -\varphi \Vert _{L^\frac{p}{4}(\Omega ; L^2(0,T;H))} \\&\quad \le C\sqrt{\varepsilon }\left( 1 + \Vert \varphi \Vert _{L^\frac{p}{4}(\Omega ; L^2(0,T;H^3(\mathcal {O})))} \right) \end{aligned}$$

holds. In particular, we have $\varphi _\varepsilon \rightarrow \varphi$ strongly in $L^\frac{p}{4}(\Omega ; L^\infty (0,T;V^*) \cap L^2(0,T;H))$.

The assumption p≥4 is not restrictive, as the initial conditions are in any case tailored for convergence (in fact, they could also be taken deterministic without much loss of generality). However, the main drawback of Theorem 2.12 is restricting the family of potentials to a subset of polynomial functions. Dropping Assumption (**A6**) yields the need of establishing ε-uniform estimates in order to retrieve a solution. To this end, an additional assumption on *G* is needed.

(**A7**): There exists a positive constant, that without loss of generality we denote with $L_G >0$, such that
  $$ \Vert G(\psi )\Vert _{\mathscr {L}^2(U,V)} \le L_G\left( 1+\Vert \psi \Vert _H \right) \qquad \forall \, \psi \in V. $$

#### Remark 2.13

Assumption (**A7**) is a technical requirement to make use of a nonlocal Poincaré inequality requiring a higher order norm of *G*. Refinements or alternative formulations of this assumption may be possible, also taking Assumption (**A3**) into account. For example, it would be sufficient to ask that

$$ \operatorname {Range}(G) \subset \mathscr {L}^2(U,W^{1,p}(\mathcal {O})) $$

for some p>d. However, this property would appear much more natural on the restriction of *G* over $W^{1,p}(\mathcal {O})$, but proving additional spatial regularity (beyond $H^1(\mathcal {O})$) on the solutions of the nonlocal Cahn–Hilliard equation appears challenging.

The last result of this work, which we prove in Section 6, reads as follows.

#### Theorem 2.14

Let Assumptions (**A1**)-(**A5**) and (**A7**) hold and let p>2. Fixed $\varepsilon _0>0$ sufficiently small, define a family of random variables $\{\varphi _{0,\varepsilon }\}_{\varepsilon \in (0,\varepsilon _0)} \subset L^p(\Omega ;H)$ such that $\varphi _{0,\varepsilon }$ satisfies the assumptions of Theorem 2.4 for all $\varepsilon \in (0,\varepsilon _0)$ and

$$ \sup _{\varepsilon \in (0,\varepsilon _0)}\mathop { \mathbb {E}}\left| \dfrac{1}{4}\int _\mathcal {O}\int _\mathcal {O}K_\varepsilon (x-y)(\varphi _{0,\varepsilon }(x)-\varphi _{0,\varepsilon }(y))^2 \, {\mathrm d}x \, {\mathrm d}y + \int _\mathcal {O}F(\varphi _{0,\varepsilon }(x)) \, {\mathrm d}x \right| ^\frac{p}{2} \le C $$

for a constant C>0 independent of ε. Let further $\varphi _0 \in L^p(\Omega , \mathscr {F}_0; V)$ and $F(\varphi _0) \in L^\frac{p}{2}(\Omega , \mathscr {F}_0;L^1(\mathcal {O}))$ and assume that $\varphi _{0,\varepsilon } \rightarrow \varphi _0$ in $L^p(\Omega ;H)$. For any $\varepsilon \in (0,\varepsilon _0)$, let $\varphi _\varepsilon$ denote the unique probabilistically-strong solution to (1.6) with respect to the initial datum $\varphi _{0,\varepsilon }$. Accordingly, let φ denote the unique probabilistically-strong solution to (2.2) with respect to the initial datum $\varphi _{0}$. Then, for any q<p, it holds

$$ \varphi _{\varepsilon } \rightarrow \varphi \quad \text {in }L^q(\Omega ;L^\infty (0,T;H)) $$

as $$\varepsilon \rightarrow 0^+$$.

## Existence of martingale solutions

The proof of existence is carried out via a two-stage approximation scheme and a stochastic compactness argument, borrowing some ideas from [36, Section 3]. The argument is split in several steps for the sake of clarity.

### The approximation scheme

As anticipated, the approximation scheme is carried out in two steps. Firstly, we recast problem (1.6) exploiting Remark 2.1, hence arriving at

3.1 $$\begin{aligned} {\left\{ \begin{array}{ll} {\mathrm d}\varphi - \Delta \mu \, {\mathrm d}t = G(\varphi ) \, {\mathrm d}W &  \quad \text {in } (0,T) \times \mathcal {O}, \\ \mu = a\varphi - K * \varphi + \Psi '(\varphi ) - \gamma \varphi &  \quad \text {in } (0,T) \times \mathcal {O}, \\ \partial _{{\boldsymbol{n}}} \mu = 0 &  \quad \text {on } (0,T) \times \partial \mathcal {O}, \\ \varphi (0) = \varphi _0 &  \quad \text {in } \mathcal {O}, \end{array}\right. } \end{aligned}$$

for some γ>0 to be defined later and such that Ψ is strongly convex. This enables us to employ a Yosida regularization scheme (see, for reference, the classical textbooks [2, 3]). Indeed, the function Ψ is a proper, convex, continuously differentiable function on $$\mathbb {R}$$ (hence, in particular, lower semicontinuous), and therefore its derivative $\Psi^{\prime }$ can be identified with a maximal monotone graph on $$\mathbb {R}\times \mathbb {R}$$. This allows us to define for all $$\lambda \in (0,1)$$ the resolvent operator $$J_\lambda $$ and the Yosida approximation $$\Psi '_\lambda $$ of $\Psi^{\prime }$, that is

$$\begin{aligned}&J_\lambda : \mathbb {R}\rightarrow \mathbb {R},\qquad J_\lambda (s) := (I+\lambda \Psi ')^{-1}(s)\qquad \forall \, s\in \mathbb {R}, \\&\Psi '_\lambda : \mathbb {R}\rightarrow \mathbb {R},\qquad \Psi '_\lambda (s) := \lambda ^{-1}(s-J_\lambda (s))\qquad \forall \, s\in \mathbb {R}, \end{aligned}$$

where *I* denotes the identity operator on $\mathbb {R}$. Now, for every $\lambda \in (0,1)$, we define the Yosida approximation of *F* by setting

3.2 $$\begin{aligned} F_\lambda : \mathbb {R} \rightarrow [0,+\infty ),\qquad F_\lambda (s) := F(0) + \int _0^s\Psi '_\lambda (x)\;\textrm{d}x - \frac{\gamma }{2}s^2\qquad \forall \, s\in \mathbb {R}. \end{aligned}$$

By construction, the function $F_\lambda$ is two times continuously differentiable and

3.3 $$\begin{aligned} F^\prime _\lambda (s) = \Psi '_\lambda (s) - \gamma s \qquad \forall \, s\in \mathbb {R} \end{aligned}$$

yielding also, by differentiating once more, that $F'_\lambda$ is Lipschitz-continuous (since $F''_\lambda$ is bounded). Owing to the considerations above, we are now in a position to introduce the Yosida approximation of problem (1.4), namely

3.4 $$\begin{aligned} {\left\{ \begin{array}{ll} {\mathrm d}\varphi _\lambda - \Delta \mu _\lambda \,{\mathrm d}t = G(\varphi _\lambda )\,{\mathrm d}W \qquad & \text {in } (0,T)\times \mathcal {O},\\ \mu _\lambda = a\varphi _\lambda -K*\varphi _\lambda + F_\lambda '(\varphi _\lambda ) \qquad & \text {in } (0,T)\times \mathcal {O},\\ \varphi _\lambda (0)=\varphi _0 \qquad & \text {in } \mathcal {O}. \end{array}\right. } \end{aligned}$$

In order to solve (3.4), we employ a discretization scheme. Indeed, let $\{(\ell _j,\, e_j)\}_{j\in \mathbb {N}_+} \subset \mathbb {R} \times D({\mathcal {A}})$ be countably many independent eigencouples of the negative Laplacian with homogeneous Neumann boundary condition, i.e., let the functions $e_j$ solve

$$ {\left\{ \begin{array}{ll} -\Delta e_j = \ell _j e_j, &  \quad \text {in } \mathcal {O}, \\ \partial _{\boldsymbol{n}}e_j = 0 &  \quad \text {on } \partial \mathcal {O}, \end{array}\right. } \qquad \forall \, j \in \mathbb {N}_+ $$

in the strong sense. Then, up to a possible renormalization, the set $\{e_j\}_{j\in \mathbb {N}_+}$ is an orthonormal system in *H* and an orthogonal system in *V*. For $n\in \mathbb {N}_+$, we now define the finite dimensional space $H_n:= \textrm{span}\{e_1,\dots ,e_n\} \subset V$, endowed with the *H*-norm. This enables us to introduce the approximate stochastic diffusion

$$ G_{n}: H_n \rightarrow \mathscr {L}^2(U,H_n) $$

such that

$$\begin{aligned} G_{n}(v)[u_k] := \sum _{j=1}^n(G(v)[u_k],e_j)_He_j \end{aligned}$$

for all $k\in \mathbb {N}$ and $v\in H_n$. Note that the operator $G_{n}$ is well-defined: indeed, for $v\in H_n$ and $n\in \mathbb {N}_+$, it holds

3.5 $$\begin{aligned} \begin{aligned} \Vert G_{n}(v)\Vert _{\mathscr {L}^2(U,H_n)}^2&= \Vert G_{n}(v)\Vert _{\mathscr {L}^2(U,H)}^2 \\  &= \sum _{k=1}^{+\infty }\sum _{j=1}^n|(G(v)[u_k],e_j)_H|^2 \\&\le \sum _{k=1}^{+\infty }\sum _{j=1}^{+\infty }|(G(v)[u_k],e_j)_H|^2 \\  &= \Vert G(v)\Vert _{\mathscr {L}^2(U,H)}^2 \end{aligned} \end{aligned}$$

and hence $G_{n}(v)\in \mathscr {L}^2(U,H_n)$ for all $v\in H_n$ and $n\in \mathbb {N}_+$. Owing to Assumption (**A3**), a similar calculation then shows that $G_{n}$ is $L_G$-Lipschitz-continuous (hence uniformly in *n*) as an operator from $H_n$ to $\mathscr {L}^2(U,H_n)$ for all $n \in \mathbb {N}_+$. Next, we define the orthogonal projection of the initial datum onto $H_n$, namely, we set

$$\begin{aligned} \varphi _{0,n} := \sum _{j=1}^n(\varphi _0,e_j)_H\,e_j \end{aligned}$$

for $n\in \mathbb {N}_+$. Now, we can formulate the discretized problem projected onto $H_n$, which reads

3.6 $$\begin{aligned} {\left\{ \begin{array}{ll} {\mathrm d}\varphi _{\lambda ,n} - \Delta \mu _{\lambda ,n}\,{\mathrm d}t = G_{n}(\varphi _{\lambda ,n})\,{\mathrm d}W \qquad & \text {in } (0,T)\times \mathcal {O},\\ \mu _{\lambda ,n} = a\varphi _{\lambda ,n} - K*\varphi _{\lambda ,n}+ F_\lambda '(\varphi _{\lambda ,n}) \qquad & \text {in } (0,T)\times \mathcal {O},\\ \partial _{{\boldsymbol{n}}}\mu _{\lambda ,n} = 0 \qquad & \text {on }(0,T)\times \partial \mathcal {O}, \\ \varphi _{\lambda ,n}(0)=\varphi _{0,n} \qquad & \text {in } \mathcal {O}. \end{array}\right. } \end{aligned}$$

For any fixed λ>0 and $n\in \mathbb {N}_+$, we look for a solution $(\varphi _{\lambda ,n},\mu _{\lambda ,n})$ to the discretized problem (3.6) of the form

$$\begin{aligned} \varphi _{\lambda ,n} = \sum _{j=1}^n {\alpha }_j^{\lambda ,n}e_j,\qquad \mu _{\lambda ,n} = \sum _{j=1}^n {\beta }_j^{\lambda ,n}e_j, \end{aligned}$$

for some suitable vector-valued processes

$$\begin{aligned}&{\alpha }^{\lambda ,n}:= ({\alpha }_1^{\lambda ,n},\ldots ,{\alpha }_n^{\lambda ,n}): \Omega \times [0,T] \rightarrow \mathbb {R}^n,\\&{\beta }^{\lambda ,n}:= ({\beta }_1^{\lambda ,n},\ldots ,{\beta }_n^{\lambda ,n}): \Omega \times [0,T] \rightarrow \mathbb {R}^n. \end{aligned}$$

Using this ansatz on $(\varphi _{\lambda ,n},\mu _{\lambda ,n})$ and choosing $e_i$ as test functions for all $i\in \{1,\ldots ,n\}$, it follows that the variational form of (3.6) is given by

3.7 $$\begin{aligned}&\int _{\mathcal {O}}\varphi _{\lambda ,n}(t)e_i\, {\mathrm d}x + \int _0^t \int _{\mathcal {O}}\nabla \mu _{\lambda ,n}(s)\cdot \nabla e_i\, {\mathrm d}x\,{\mathrm d}s\nonumber \\&\quad = \int _{\mathcal {O}}\varphi _{0,n}e_i\, {\mathrm d}x + \int _{\mathcal {O}}\left( \int _0^t G_{n}(\varphi _{\lambda ,n}(s))\, {\mathrm d}W(s)\right) e_i\, {\mathrm d}x \end{aligned}$$

for all $i \in \{1,\ldots ,n\}$ and every t∈[0,T], $\mathbb {P}$-almost surely, as well as

3.8 $$\begin{aligned} \int _{\mathcal {O}}\mu _{\lambda ,n}(t)e_i\, {\mathrm d}x = \int _{\mathcal {O}}{a\varphi _{\lambda ,n}(t)e_i}\,{\mathrm d}x - \int _{\mathcal {O}}{[K*\varphi _{\lambda ,n}(t)]e_i}\,{\mathrm d}x + \int _{\mathcal {O}}F^\prime _\lambda (\varphi _{\lambda ,n}(t))e_i \,{\mathrm d}x \end{aligned}$$

for all $i \in \{1,\ldots ,n\}$ and every t∈[0,T], $\mathbb {P}$-almost surely. Due to the orthogonality properties of $\{e_i\}_{i\in \mathbb {N}_+}$, we deduce that the processes ${\alpha }^{\lambda ,n}$ and ${\beta }^{\lambda ,n}$ satisfy the following ordinary stochastic differential equations,

3.9 $$\begin{aligned} {\left\{ \begin{array}{ll} \displaystyle {\mathrm d}{\alpha }_i^{\lambda ,n} + {\ell _i\beta }_i^{\lambda ,n} = \left( G_{n}\left( \sum _{l=1}^n {\alpha }_l^{\lambda ,n}e_l\right) {\mathrm d}W,\,e_i\right) _H, \\ \displaystyle {\beta }_i^{\lambda ,n} = \sum _{j=1}^n{\alpha }_j^{\lambda ,n}\int _\mathcal {O}ae_je_i \, {\mathrm d}x - \sum _{j=1}^n{\alpha _j^{\lambda ,n} \int _\mathcal {O}[K*e_j]e_i\, {\mathrm d}x} + \int _\mathcal {O}F^\prime _{\lambda }\left( \sum _{l=1}^n{\alpha }_l^{\lambda ,n}e_l\right) e_i \, {\mathrm d}x, \\ \displaystyle a_i^{\lambda ,n}(0) = (\varphi _0,e_i)_H \end{array}\right. } \end{aligned}$$

for all $i \in \{1,\ldots ,n\}$. Note that the stochastic integrals on the right-hand side have to be regarded, for all $i \in \{1,\ldots ,n\}$, as $G_i^{n}\,{\mathrm d}W$, where

$$\begin{aligned} G_i^{n}: H_n \rightarrow \mathscr {L}^2(U,\mathbb {R}),\qquad G_i^{n}\left( \sum _{l=1}^nc_le_l\right) [u_k] := \left( G_n\left( \sum _{l=1}^nc_le_l\right) {[u_k]},e_i\right) _H \end{aligned}$$

for all $k\in \mathbb {N}$. Since all the nonlinearities appearing in the discretized problem (3.9) are Lipschitz continuous, the theory of abstract stochastic evolution equations applies. Hence, there exists a unique solution

$$\begin{aligned} {\alpha }^{\lambda ,n},\, {\beta }^{\lambda ,n} \in L^{p}(\Omega ;C^0([0,T];\mathbb {R}^n)). \end{aligned}$$

Therefore, the approximated problem (3.9) admits a unique solution

$$\begin{aligned} \varphi _{\lambda ,n},\, \mu _{\lambda ,n}\in L^p(\Omega ;C^0([0,T];H_n)) \end{aligned}$$

where *p* is given by the moment regularity of initial conditions.

### Uniform estimates with respect to *n*

Next, we prove that the approximated solutions $(\varphi _{\lambda ,n},\mu _{\lambda ,n})$ satisfy some basic regularity estimates uniformly in *n*, while keeping λ>0 fixed. Here, the symbol *C* (possibly indexed) denotes a positive constant whose special dependencies are explicitly pointed out, if necessary. In particular, throughout this subsection, the dependence on λ is always explicited by the symbol $C_\lambda$. Before continuing, we point out a key preliminary lemma.

#### Lemma 3.1

Assume Assumptions (**A1**) and (**A2**). There exists γ>0 such that

$$ F_\lambda ''(s) + a(x) = \Psi ''_\lambda (s) - \gamma + a(x) \ge \dfrac{C_0}{2} $$

for all $s \in \mathbb {R}$, almost every $x \in \mathcal {O}$ and all $\lambda \in (0,1]$.

#### Proof

As established in Remark 2.1, for every $s \in \mathbb {R}$ we have

$$ \Psi ''(s) \ge \gamma - \alpha > 0 $$

and invoking, for instance, [25, Lemma 2.10], we get

3.10 $$\begin{aligned} \Psi ''_\lambda (s) \ge \dfrac{\gamma -\alpha }{1+\gamma -\alpha }, \end{aligned}$$

for all $s \in \mathbb {R}$ and for all $\lambda \in (0,1]$. Therefore, recalling that $a(x) \ge C_0 + \alpha$ almost everywhere in $\mathcal {O}$,

$$ \begin{aligned} \Psi _\lambda ''(s) - \gamma + a(x)&\ge \dfrac{\gamma -\alpha }{1+\gamma -\alpha } - \gamma + C_0 + \alpha \\&= - \dfrac{(\gamma -\alpha )^2}{1+\gamma -\alpha } + C_0 \end{aligned} $$

and as

$$ x \mapsto \dfrac{x^2}{1+x} $$

vanishes as $x \rightarrow 0^+$, we can choose $\gamma -\alpha$ small enough in such a way that the claim holds. □

In all of the following, in light of Lemma 3.1, we shall implicitly assume, without loss of generality, that $\lambda \le 1$. Moreover, we set γ>0 in (3.1) in such a way that Lemma 3.1 holds. The first estimate reads as follows.

#### Lemma 3.2

The sequence $\{\varphi _{\lambda , n}\}_{n \in \mathbb {N}}$ is uniformly bounded in $L^p(\Omega ;C^0([0,T];H)) \cap L^p(\Omega ;L^2(0,T;V))$ for all p≥2.

#### Proof

We apply the Itô formula for the *H*-norm of $\varphi _{\lambda ,n}$ (see, for instance, [8, Theorem 4.32] or [33, Theorem 4.2.5]). This yields

3.11 $$\begin{aligned} \Vert \varphi _{\lambda ,n}(t)\Vert _H^2&= \Vert \varphi _{0,n}\Vert _H^2 + 2\int _0^t(\Delta \mu _{\lambda ,n}(s),\varphi _{\lambda ,n}(s))_{H} + \Vert G_{n}(\varphi _{\lambda ,n}(s))\Vert _{\mathscr {L}^2(U,H_n)}^2\, {\mathrm d}s \nonumber \\&\quad + 2\int _0^t\big (\varphi _{\lambda ,n}(s),G_n(\varphi _{\lambda ,n}(s))\, {\mathrm d}W(s)\big )_H. \end{aligned}$$

Recalling the definition of the chemical potential and the properties of convolutions, as well as appealing to Lemma 3.1, we obtain

$$\begin{aligned} (-\Delta \mu _{\lambda ,n},\varphi _{\lambda ,n})_{H}&= (\nabla \mu _{\lambda ,n},\nabla \varphi _{\lambda ,n})_{{\boldsymbol{H}}} \\&= (a\nabla \varphi _{\lambda ,n} + \varphi _{\lambda ,n}\nabla a - \nabla {K}*\varphi _{\lambda ,n}+ F''_\lambda (\varphi _{\lambda ,n})\nabla \varphi _{\lambda ,n},\,\nabla \varphi _{\lambda ,n})_{{\boldsymbol{H}}} \\&\ge \dfrac{C_0}{2}\Vert \nabla \varphi _{\lambda ,n}\Vert _{\boldsymbol{H}}^2 - 2\Vert \nabla {K}\Vert _{{\boldsymbol{L}}^1({B_R})}\Vert \nabla \varphi _{\lambda ,n}\Vert _{\boldsymbol{H}}\Vert \varphi _{\lambda ,n}\Vert _H \\&\ge \frac{C_0}{4} \Vert \nabla \varphi _{\lambda ,n}\Vert _{\boldsymbol{H}}^2 - \dfrac{4}{C_0}{\Vert \nabla {K}\Vert _{{\boldsymbol{L}}^1({B_R})}^2}\Vert \varphi _{\lambda ,n}\Vert _H^2. \end{aligned}$$

Moreover, thanks to (3.5) and Assumption (**A3**) it holds

$$\begin{aligned} \begin{aligned} \Vert G_{n}(\varphi _{\lambda ,n})\Vert _{\mathscr {L}^2(U,H_n)}^2&\le \Vert G(\varphi _{\lambda ,n})\Vert _{\mathscr {L}^2(U,H)}^2 \\&\le L_G^2 \end{aligned} \end{aligned}$$

for all $n \in \mathbb {N}$. Combining the above estimates, we have

3.12 $$\begin{aligned}  &   \Vert \varphi _{\lambda ,n}(t)\Vert _H^2 + \dfrac{C_0}{2}\int _0^t\Vert \nabla \varphi _{\lambda ,n}(s)\Vert _{\boldsymbol{H}}^2 \, {\mathrm d}s \nonumber \\    &   \quad \le \Vert \varphi _{0,n}\Vert _H^2 + 4\left( \dfrac{2}{C_0}\Vert \nabla K\Vert ^2_{{\boldsymbol{L}}^1(B_R)} + L_G^2\right) \int _0^t\Vert \varphi _{\lambda ,n}(s)\Vert _H^2 \, {\mathrm d}s + 2L_G^2t \nonumber \\    &   \quad \quad \quad \quad \quad \quad \quad \quad \quad \quad + 2\int _0^t\big (\varphi _{\lambda ,n}(s),G_n(\varphi _{\lambda ,n}(s)) \,{\mathrm d}W(s)\big )_H \end{aligned}$$

for all $n \in \mathbb {N}$. Note that by linearity and 1-Lipschitz continuity of the projection $\Pi _n: H\rightarrow H_n$, it follows

$$\begin{aligned} \Vert \varphi _{0,n}\Vert _H^2 \le \Vert \varphi _{0}\Vert _H^2. \end{aligned}$$

Next, raising (3.12) to the power $\frac{p}{2}$, then taking the supremum on the interval [0, *t*] and $\mathbb {P}$-expectations, we eventually arrive at

$$\begin{aligned}&\mathop { \mathbb {E}}\sup _{\tau \in [0,\,t]}\Vert \varphi _{\lambda ,n}(\tau )\Vert _H^p + \mathop { \mathbb {E}}\left| \int _0^t\Vert \nabla \varphi _{\lambda ,n}(\tau )\Vert _{\boldsymbol{H}}^2\, {\mathrm d}\tau \right| ^{\frac{p}{2}} \\&\le C\left( 1 + \mathop { \mathbb {E}}\Vert \varphi _{0}\Vert _H^p + \left| \mathop { \mathbb {E}}\int _0^t\Vert \varphi _{\lambda ,n}(\tau )\Vert _H^2 \, {\mathrm d}\tau \right| ^\frac{p}{2} \, {\mathrm d}\tau \right. \\&\left. \quad + \mathop { \mathbb {E}}\sup _{\tau \in [0,\,t]}\left| \int _0^\tau \big (\varphi _{\lambda ,n}(s),G_n(\varphi _{\lambda ,n}(s)) \, {\mathrm d}W(s)\big )_H\right| ^{\frac{p}{2}}\right) , \end{aligned}$$

where the constant *C* only depends on the structural parameters of the problem, including *p* and *T*. Finally, the Burkholder–Davis–Gundy and Hölder inequalities imply

3.13 $$\begin{aligned}&\mathop { \mathbb {E}}\sup _{{\tau } \in [0,t]}\left| \int _0^{{\tau }}\big (\varphi _{\lambda ,n}({s}),G_n(\varphi _{\lambda ,n}({s}))\, {\mathrm d}W({s})\big )_H\right| ^{\frac{p}{2}} \nonumber \\&\qquad \le C\mathop { \mathbb {E}}\left| \int _0^t\Vert \varphi _{\lambda ,n}({s})\Vert _H^2\Vert G_n(\varphi _{\lambda ,n}({s}))\Vert _{\mathscr {L}^2(U,H_n)}^2 \,{\mathrm d}{s}\right| ^{\frac{p}{4}} \nonumber \\&\qquad \le C\mathop { \mathbb {E}}\left| \sup _{\tau \in [0,\,t]}\Vert \varphi _{\lambda ,n}(\tau )\Vert _H^2\int _0^t\Vert G_n(\varphi _{\lambda ,n}({s}))\Vert _{\mathscr {L}^2(U,H_n)}^2\, {\mathrm d}{s}\right| ^{\frac{p}{4}} \nonumber \\&\qquad \le Ct^\frac{p}{4}\mathop { \mathbb {E}}\left| \sup _{\tau \in [0,\,t]}\Vert \varphi _{\lambda ,n}(\tau )\Vert _H^2\right| ^{\frac{p}{4}} \nonumber \\&\qquad \le \dfrac{1}{2}\mathop { \mathbb {E}}\sup _{\tau \in [0,\,t]}\Vert \varphi _{\lambda ,n}(\tau )\Vert _H^{p} + Ct^{\frac{p}{2}} \end{aligned}$$

where the constant *C* depends on the structural parameters of the problem, including *p*. Hence, thanks to (3.13) and Young’s inequality, we arrive at

$$\begin{aligned}&\mathop { \mathbb {E}}\sup _{\tau \in [0,\,t]}\Vert \varphi _{\lambda ,n}(\tau )\Vert _H^p + \mathop { \mathbb {E}}\left| \int _0^t\Vert \nabla \varphi _{\lambda ,n}(\tau )\Vert _{\boldsymbol{H}}^2 \, {\mathrm d}\tau \right| ^{\frac{p}{2}}\\&\quad \le C\left( 1 + \mathop { \mathbb {E}}\Vert \varphi _{0}\Vert _H^p + \mathop { \mathbb {E}}\left| \int _0^t\Vert \varphi _{\lambda ,n}(\tau )\Vert _H^2 \, {\mathrm d}\tau \right| ^\frac{p}{2}\right) . \end{aligned}$$

Finally, an application of Gronwall’s lemma implies that there exists some constant $C_1$, independent of *n* and λ, such that

3.14 $$\begin{aligned} \Vert \varphi _{\lambda ,n}\Vert _{L^p(\Omega ;C^0([0,T];H))} + \Vert \varphi _{\lambda ,n}\Vert _{L^p(\Omega ;L^2(0,T;V))} \le C_1 \end{aligned}$$

and the first estimate is proved. □

A second useful estimate is an energy inequality. In particular, we want to apply the Itô formula (see [8, Theorem 4.32]) to the approximated free energy functional

$$\begin{aligned} \mathcal {E}_\lambda : H_n \rightarrow \mathbb {R}, \quad \mathcal {E}_\lambda (v)&:= \frac{1}{4}\int _\mathcal {O}\int _\mathcal {O}K(x-y)|v(x) - v(y)|^2 \, {\mathrm d}y \, {\mathrm d}x \\&\quad + \int _\mathcal {O}F_\lambda (v(x)) \, {\mathrm d}x, \end{aligned}$$

for all $v \in H_n$. In the following, for convenience, we treat the nonlocal part of $\mathcal {E}_\lambda$ as a bilinear nonlocal operator $\Lambda : H_n \times H_n \rightarrow \mathbb {R}$, so that

3.15 $$\begin{aligned} \begin{aligned} \Lambda (h, k)&= \int _\mathcal {O}\int _\mathcal {O}K(x-y)(h(x)-h(y))(k(x)-k(y))\, {\mathrm d}y\,{\mathrm d}x \\&= 2(1, K*hk)_H-2(h, K*k)_H \end{aligned} \end{aligned}$$

the equality holding by means of Assumption (**A1**), ensuring that the convolution operator is a symmetric map on H×H.

#### Lemma 3.3

The bilinear form Λ is well defined on H×H. Moreover, Λ is bounded, i.e., there exists a positive constant C>0 such that

$$ |\Lambda (h, k)| \le C\Vert h\Vert _H\Vert k\Vert _H $$

for all *h* and k∈H.

#### Proof

Let *h* and *k* to be arbitrary but fixed elements of *H*. The claim is a direct consequence of Young’s convolutional inequality. Indeed,

$$ \begin{aligned} |\Lambda (h, k)|&= |2(1, K*hk)_H-2(h, K*k)_H| \\&\le 2|(1, K*hk)_H|+ 2|(h, K*k)_H| \\&\le 2\Vert 1\Vert _{L^\infty (\mathcal {O})}\Vert K * hk\Vert _{L^1(\mathcal {O})} + 2\Vert h\Vert _H\Vert K*k\Vert _H \\&\le 2\Vert K\Vert _{L^1(B_R)} \left( \Vert hk\Vert _{L^1(\mathcal {O})} + \Vert h\Vert _H\Vert k\Vert _H \right) \\&\le 4\Vert K\Vert _{L^1(B_R)}\Vert h\Vert _H\Vert k\Vert _H, \end{aligned} $$

and the proof is complete. □

Moreover, let us show a simple, yet crucial property of the approximated energy functional.

#### Lemma 3.4

For any constant M>0, there exists $\eta = \eta (M) > 0$ and a constant $C_\eta > 0$ only depending on η such that

$$ \mathcal {E}_\lambda (v) > M\Vert v\Vert ^2_H- C_\eta $$

for all v∈H and all $\lambda \in (0,\eta )$.

#### Proof

Fix v∈H and η>0, and let $\lambda \in (0, \eta )$. Applying [25, Lemma 2.11] and owing to the computations in the proof of Lemma 3.3, we get

$$ \begin{aligned} \mathcal {E}_\lambda (v)&= \dfrac{1}{4} \Lambda (v, v) + \int _\mathcal {O}F_\lambda (v(x)) \, {\mathrm d}x \\&= \dfrac{1}{4} \Lambda (v, v) + \int _\mathcal {O}\Psi _\lambda (v(x)) - {\dfrac{\gamma }{2}}v^2(x) \, {\mathrm d}x + |\mathcal {O}|F(0) \\&\ge \dfrac{1}{4\eta }\Vert v\Vert ^2_H - \Vert K\Vert _{L^1(\mathcal {O})}\Vert v\Vert ^2_H -{\dfrac{\gamma }{2}}\Vert v\Vert ^2_H - C_\eta \\&= \left( \dfrac{1}{4\eta } - \Vert K\Vert _{L^1(\mathcal {O})} - \gamma \right) \Vert v\Vert ^2_H - C_\eta , \end{aligned} $$

so that by choosing η>0 such that

$$ \dfrac{1}{4\eta } - \Vert K\Vert _{L^1(\mathcal {O})} - \gamma > {M} \Leftrightarrow \eta < \dfrac{1}{4({M}+\Vert K\Vert _{L^1(\mathcal {O})}+\gamma )} $$

the proof is complete. □

Lemma 3.4 implies that the approximated energy functional is almost coercive if λ is sufficiently small. Without loss of generality, we shall then assume that, for some fixed $0<{\nu }< \eta$,

$$ \lambda < \min \{{\nu }, 1\} $$

and consider, for convenience and without relabeling, $\mathcal {E}_\lambda$ to be coercive by adding the constant $C_{{\nu }}$, i.e.,

$$\begin{aligned} \mathcal {E}_\lambda : H_n \rightarrow \mathbb {R} \quad \mathcal {E}_\lambda (v)&:= \frac{1}{4}\int _\mathcal {O}\int _\mathcal {O}K(x-y)|v(x) - v(y)|^2 \, {\mathrm d}y \, {\mathrm d}x \\&\quad + \int _\mathcal {O}F_\lambda (v(x)) \, {\mathrm d}x + C_{{\nu }},\qquad \forall \, v\in H_n. \end{aligned}$$

In order to apply the Itô lemma, we need to show that $\mathcal {E}_\lambda$ is twice Fréchet differentiable. Observe that the constant $C_{{\nu }}$ does not affect any derivative of $\mathcal {E}_\lambda$.

#### Lemma 3.5

The functional $\mathcal {E}_\lambda$ is twice Fréchet differentiable with uniformly continuous derivatives on bounded subsets of $H_n$.

#### Proof

First, observe that $\mathcal {E}_\lambda$ is indeed Fréchet-differentiable with $D\mathcal {E}_\lambda : H_n\rightarrow H_n^*$ given by

$$\begin{aligned} D\mathcal {E}_\lambda (v)[h] := \frac{1}{2}\Lambda (v, h) + \int _\mathcal {O}F^\prime _\lambda (v(x))h(x) \, {\mathrm d}x,\qquad \forall \, v,\,h\in H_n. \end{aligned}$$

Indeed, for any $v,\,h\in H_n$, we have

$$\begin{aligned}&\mathcal {E}_\lambda (v+h) - \mathcal {E}_\lambda (v) -D\mathcal {E}_\lambda (v)[h] \\&\qquad = \frac{1}{4}\int _\mathcal {O}\int _\mathcal {O}K(x-y)|v(x)+h(x)-v(y)-h(y)|^2\, {\mathrm d}y\, {\mathrm d}x + \int _\mathcal {O}F_\lambda (v(x)+h(x))\,{\mathrm d}x \\&\qquad \qquad - \frac{1}{4}\int _\mathcal {O}\int _\mathcal {O}K(x-y)|v(x)-v(y)|^2\, {\mathrm d}y\, {\mathrm d}x - \int _\mathcal {O}F_\lambda (v(x))\, {\mathrm d}x \\&\qquad \qquad - \frac{1}{2}\int _\mathcal {O}\int _\mathcal {O}K(x-y)(v(x)-v(y))(h(x)-h(y))\, {\mathrm d}y\, {\mathrm d}x - \int _\mathcal {O}F^\prime _\lambda (v(x))h(x)\, {\mathrm d}x. \end{aligned}$$

Using the identity

$$\begin{aligned} |v(x)-v(y)+h(x)-h(y)|^2&= |v(x)-v(y)|^2 + |h(x)-h(y)|^2 \\&\quad + 2(v(x)-v(y))(h(x)-h(y)), \end{aligned}$$

as well as the fundamental theorem of calculus, we obtain

$$\begin{aligned}&\big |\mathcal {E}_\lambda (v+h) - \mathcal {E}_\lambda (v) -D\mathcal {E}_\lambda (v)h\big | \\&\le \frac{1}{4}\int _\mathcal {O}\int _\mathcal {O}K(x-y)|h(x)-h(y)|^2\, {\mathrm d}y\, {\mathrm d}x \\&\quad + \left| \int _\mathcal {O}F_\lambda (v(x)+h(x))\, {\mathrm d}x - \int _\mathcal {O}F_\lambda (v(x))\, {\mathrm d}x - \int _\mathcal {O}F^\prime _\lambda (v(x))h(x)\, {\mathrm d}x\right| \\&= \frac{1}{4}\int _\mathcal {O}\int _\mathcal {O}K(x-y)|h(x)-h(y)|^2\,{\mathrm d}y\,{\mathrm d}x \\&\quad + \left| \int _0^1\int _\mathcal {O}F^\prime _\lambda (v(x)+th(x))h(x)\, {\mathrm d}x\, {\mathrm d}t - \int _\mathcal {O}F^\prime _\lambda (v(x))h(x)\, {\mathrm d}x\right| \\&= \frac{1}{4}\int _\mathcal {O}\int _\mathcal {O}K(x-y)|h(x)-h(y)|^2\, {\mathrm d}y\, {\mathrm d}x \\&\quad + \left| \int _0^1\int _\mathcal {O}\Big (F^\prime _\lambda (v(x)+th(x)) - F^\prime _\lambda (v(x))\Big )h(x)\, {\mathrm d}x\, {\mathrm d}t\right| \\&\le C\Vert h\Vert ^2_H + C_\lambda \Vert h\Vert _H^2 \\&\le C_\lambda \Vert h\Vert _{H_n}^2, \end{aligned}$$

where we used Lemma 3.3, Assumption (**A1**) and the Lipschitz continuity of $F_\lambda ^\prime$. This shows that $\mathcal {E}_\lambda$ is Fréchet differentiable. Now, let us show that also $D\mathcal {E}_\lambda$ is Fréchet differentiable with $D^2\mathcal {E}_\lambda : H_n \rightarrow \mathscr {L}(H_n,H_n^*)$ given by

$$\begin{aligned} D^2\mathcal {E}_\lambda (v)[h,k] := \frac{1}{2}\Lambda (h,k)+ \int _\mathcal {O}F^{\prime \prime }_\lambda (v(x))h(x)k(x)\,{\mathrm d}x \end{aligned}$$

for any $v,h,k\in H_n$. In fact, for every $v,h,k\in H_n$, it holds

$$\begin{aligned}&\left| D\mathcal {E}_\lambda (v+k){[h]} - D\mathcal {E}_\lambda (v){[h]} - D^2\mathcal {E}_\lambda (v)[h,k]\right| \\&\qquad = \left| \int _\mathcal {O}F^\prime _\lambda (v(x)+k(x))h(x)\,{\mathrm d}x - \int _\mathcal {O}F^\prime _\lambda (v(x))h(x)\,{\mathrm d}x - \int _\mathcal {O}F^{\prime \prime }_\lambda (v(x))h(x)k(x)\,{\mathrm d}x\right| \\&\qquad = \left| \int _0^1\int _\mathcal {O}\big (F^{\prime \prime }_\lambda (v(x)+tk(x))-F^{\prime \prime }_\lambda (v(x))\big )h(x)k(x)\,{\mathrm d}x\,{\mathrm d}t\right| \\&\qquad \le \Vert h\Vert _{L^\infty (\mathcal {O})}\Vert k\Vert _{L^\infty (\mathcal {O})}\int _0^1\Vert F^{\prime \prime }_\lambda (v+tk)-F^{\prime \prime }_\lambda (v)\Vert _{L^1(\mathcal {O})}\,{\mathrm d}t \\&\qquad \le C\Vert h\Vert _{H_n}\Vert k\Vert _{H_n}\int _0^1\Vert F^{\prime \prime }_\lambda (v+tk)-F^{\prime \prime }_\lambda (v)\Vert _{L^1(\mathcal {O})}\,{\mathrm d}t, \end{aligned}$$

where we used Hölder’s inequality and the continuous embedding $H_n \hookrightarrow L^\infty (\mathcal {O})$. Recalling that $F_\lambda ^{\prime \prime }$ is continuous and bounded, the dominated convergence theorem yields

$$\begin{aligned} \int _0^1\Vert F^{\prime \prime }_\lambda (v+tk)-F^{\prime \prime }_\lambda (v)\Vert _{L^1(\mathcal {O})}\,{\mathrm d}t \rightarrow 0 \qquad \text { as }\Vert k\Vert _{H_n}\rightarrow 0. \end{aligned}$$

Hence, we obtain

$$\begin{aligned} \sup _{\Vert h\Vert _{H_n}\le 1}\Big |D\mathcal {E}_\lambda (v+k){[h]} - D\mathcal {E}_\lambda (v){[h]} - D^2\mathcal {E}_\lambda (v)[h,k]\Big | = {o}(\Vert k\Vert _{H_n})\qquad \text { as }\Vert k\Vert _{H_n}\rightarrow 0. \end{aligned}$$

In particular, this shows that $D\mathcal {E}_\lambda$ is Fréchet-differentiable with derivative $D^2\mathcal {E}_\lambda$ as above. Moreover, we note that by similar arguments, it is easily shown that both $D\mathcal {E}_\lambda$ and $D^2\mathcal {E}_\lambda$ are uniformly continuous and bounded on bounded subsets of $H_n$. Indeed, this follows from Lemma 3.3, as well as the facts that $F^\prime _\lambda$ is Lipschitz-continuous and that $F^{\prime \prime }_\lambda$ is continuous and bounded. □

Owing to the previous lemma, we are in a position to show the next estimate.

#### Lemma 3.6

It holds that

(i) the sequence $\{\mu _{\lambda ,n}\}_{n \in \mathbb {N}}$ is uniformly bounded in $L^p(\Omega ;L^2(0,T;V))$;
(ii) the sequence $\{\Psi _\lambda (\varphi _{\lambda , n})\}_{n \in \mathbb {N}}$ is uniformly bounded in $L^\frac{p}{2}(\Omega ; L^\infty (0,T;L^1(\mathcal {O})))$;

for any p≥2.

#### Proof

First of all, we note that $D\mathcal {E}_\lambda (\varphi _{\lambda ,n}) = \mu _{\lambda ,n}$ due to Assumption (**A1**). Hence, applying the Itô lemma to the regularized energy functional $\mathcal {E}_\lambda$, we obtain

3.16 $$\begin{aligned}&\mathcal {E}_\lambda (\varphi _{\lambda ,n}(t)) + \int _0^t\int _\mathcal {O}|\nabla \mu _{\lambda ,n}(s)|^2\,{\mathrm d}x\,{\mathrm d}s\nonumber \\&\quad = \mathcal {E}_\lambda (\varphi _{0,n}) + \int _0^t\Big (\mu _{\lambda ,n}(s),G_n(\varphi _{\lambda ,n}(s))\,{\mathrm d}W(s)\Big )_H \nonumber \\&\qquad + \frac{1}{2}\int _0^t\sum _{k=0}^{+\infty }\left[ \frac{1}{2}\Lambda (G_n(\varphi _{\lambda ,n}(s))[u_k], G_n(\varphi _{\lambda ,n}(s))[u_k])\right. \nonumber \\&\qquad \left. +\int _\mathcal {O}F^{\prime \prime }_\lambda (\varphi _{\lambda ,n}(s))|G_n(\varphi _{\lambda ,n}(s))[u_k]|^2\,{\mathrm d}x \right] \,{\mathrm d}s. \end{aligned}$$

Appealing to Lemma 3.3 and Assumption (**A3**) yields

$$ \begin{aligned} \int _0^t\sum _{k=0}^{+\infty }\left| \Lambda (G_n(\varphi _{\lambda ,n}(s))[u_k], G_n(\varphi _{\lambda ,n}(s))[u_k])\right| \, {\mathrm d}s&\le C\int _0^t \Vert G_n(\varphi _{\lambda , n}(s))\Vert ^2_{\mathscr {L}^2(U, H)} \, {\mathrm d}s \\&\le C t, \end{aligned} $$

and similarly

$$ \begin{aligned} \int _0^t\sum _{k=0}^{+\infty }\int _\mathcal {O}F^{\prime \prime }_\lambda (\varphi _{\lambda ,n}(s))|G_n(\varphi _{\lambda ,n}(s))[u_k]|^2\,{\mathrm d}x \, {\mathrm d}s&\le C_\lambda \int _0^t \Vert G_n(\varphi _{\lambda , n}(s))\Vert ^2_{\mathscr {L}^2(U, H)} \, {\mathrm d}s \\&\le C_\lambda t. \end{aligned} $$

As far as the initial energy is concerned, once again by Lemma 3.3 and the boundedness of $F''_\lambda$ we have

$$ |\mathcal {E}_\lambda (\varphi _{0,n})| \le C_\lambda \left( 1 + \Vert \varphi _{0,n}\Vert ^2_H \right) \le C_\lambda \left( 1 + \Vert \varphi _{0}\Vert ^2_H \right) , $$

where we also used the fact that $\Vert \varphi _{0,n}\Vert _H \le \Vert \varphi _0\Vert _H$ by construction, $\mathbb {P}$-almost surely. Combining the above estimates, and then taking $\frac{p}{2}$-powers, the supremum over $[0,{t}]$ and $\mathbb {P}$-expectations in (3.16), we get, recalling the definition of $\mathcal {E}_\lambda$

3.17 $$\begin{aligned}  &   \mathop { \mathbb {E}}\sup _{\tau \in [0,\,t]}\mathcal {E}_\lambda ^\frac{p}{2}(\varphi _{\lambda , n}(\tau )) + \mathop { \mathbb {E}}\left| \int _0^t \Vert \nabla \mu _{\lambda ,n}(\tau )\Vert _{{\boldsymbol{H}}}^2 \, {\mathrm d}\tau \right| ^\frac{p}{2} \ \nonumber \\  &   \le C_\lambda \left( 1+\mathop { \mathbb {E}}\Vert \varphi _{0}\Vert _H^{p} +\mathop { \mathbb {E}}\sup _{\tau \in [0,\,t]}\left| \int _0^\tau \big (\mu _{\lambda ,n}(s),G_n(\varphi _{\lambda ,n}(s))\,{\mathrm d}W(s)\big )_H\right| ^{\frac{p}{2}} \right) , \end{aligned}$$

where the constant $C_\lambda$ is independent of *n*, but depends on λ and *T*. Next, by the Burkholder–Davis–Gundy inequality and (3.5), we have

3.18 $$\begin{aligned} \begin{aligned}&\mathop { \mathbb {E}}\sup _{\tau \in [0,\,t]}\left| \int _0^\tau \big (\mu _{\lambda ,n}(s),G_n(\varphi _{\lambda ,n}(s)) \, {\mathrm d}W(s)\big )_H\right| ^{\frac{p}{2}} \\&\qquad \le C\mathop { \mathbb {E}}\left[ \int _0^t\Vert \mu _{\lambda ,n}(s)\Vert _H^2\Vert G_n(\varphi _{\lambda ,n}(s))\Vert _{\mathscr {L}^2(U,H)}^2 \, {\mathrm d}s\right] ^{\frac{p}{4}} \\&\qquad \le C\mathop { \mathbb {E}}\left[ \sup _{\tau \in [0,\,t]}\Vert G_n(\varphi _{\lambda ,n}(\tau ))\Vert _{\mathscr {L}^2(U,H)}^2\int _0^t\Vert \mu _{\lambda ,n}(s)\Vert _{H}^2 \, {\mathrm d}s\right] ^{\frac{p}{4}} \\&\qquad \le C\mathop { \mathbb {E}}\left[ \int _0^t\Vert \mu _{\lambda ,n}(s)\Vert _{H}^2 \, {\mathrm d}s\right] ^{\frac{p}{4}} \\&\qquad \le \varepsilon \mathop { \mathbb {E}}\left| \int _0^t \Vert \mu _{\lambda ,n}(s)\Vert ^2_{H} \, {\mathrm d}s \right| ^{\frac{p}{2}} + C \end{aligned} \end{aligned}$$

where ε>0 is arbitrary and *C* is independent of both *n* and λ. In order to handle the last term, we resort to the Poincaré inequality. Choosing ε>0 suitably and by summing and subtracting the integral average of the approximated chemical potential, we have

3.19 $$\begin{aligned} \varepsilon \mathop { \mathbb {E}}\left| \int _0^t \Vert \mu _{\lambda ,n}(s)\Vert ^2_{H} \, {\mathrm d}s \right| ^{\frac{p}{2}} \le \dfrac{1}{2} \mathop { \mathbb {E}}\left| \int _0^t \Vert \nabla \mu _{\lambda ,n}(s)\Vert ^2_{{\boldsymbol{H}}} \, {\mathrm d}s \right| ^{\frac{p}{2}} + C\mathop { \mathbb {E}}\left| \int _0^t |\overline{\mu _{\lambda , n}(s)}|^2 \, {\mathrm d}s \right| ^{\frac{p}{2}}. \end{aligned}$$

Testing the equation for the approximated chemical potential in (3.6) by 1, we have

$$\begin{aligned} |\mathcal {O}|\overline{\mu _{\lambda ,n}} = (\mu _{\lambda ,n}, 1)_H = (a\varphi _{\lambda ,n}, 1)_H + (K * \varphi _{\lambda ,n}, 1)_H + (F^\prime _\lambda (\varphi _{\lambda ,n}), 1)_H, \end{aligned}$$

implying, recalling that $F'_\lambda$ is linearly bounded,

3.20 $$\begin{aligned} \mathop { \mathbb {E}}\left| \int _0^t |\overline{\mu _{\lambda , n}(s)}|^2 \, {\mathrm d}s \right| ^{\frac{p}{2}} \le C_\lambda \left( 1 + \mathop { \mathbb {E}}\left| \int _0^t \Vert \varphi _{\lambda , n}(s)\Vert _H^2 \, {\mathrm d}s \right| ^{\frac{p}{2}}\right) \end{aligned}$$

via Young’s convolutional inequality and the Hölder inequality. Combining all of the above in (3.17), also recalling Lemma 3.2, we arrive at

$$\begin{aligned} \mathop { \mathbb {E}}\sup _{\tau \in [0,\,t]}\mathcal {E}_\lambda ^\frac{p}{2}(\varphi _{\lambda , n}(\tau )) + \mathop { \mathbb {E}}\left| \int _0^t \Vert \nabla \mu _{\lambda ,n}(\tau )\Vert _{{\boldsymbol{H}}}^2 \, {\mathrm d}\tau \right| ^\frac{p}{2} \le C_\lambda \left( 1+\Vert \varphi _{0}\Vert _H^{p} \right) . \end{aligned}$$

The Gronwall lemma and (3.20) then give

3.21 $$\begin{aligned} \Vert \mu _{\lambda ,n}\Vert _{L^p(\Omega ;L^2(0,T;V))}\le C_\lambda . \end{aligned}$$

Finally, arguing by comparison observe that

$$ \left| \int _\mathcal {O}\Psi '_\lambda (\varphi _{\lambda , n}) \, {\mathrm d}x\right| \le |\mathcal {E}_\lambda (\varphi _{\lambda , n})| + C\Vert \varphi _{\lambda , n}\Vert ^2_H + C_\Lambda $$

and hence we conclude that

$$\begin{aligned} \Vert \mu _{\lambda ,n}\Vert _{L^p(\Omega ;L^2(0,T;V))} + \Vert \Psi _\lambda (\varphi _{\lambda , n})\Vert _{L^\frac{p}{2}(\Omega ; L^\infty (0,T;L^1(\mathcal {O})))} \le C_{2, \lambda }, \end{aligned}$$

and the proof is complete. □

Concerning the stochastic integrals, we have the following result.

#### Lemma 3.7

It holds that

(i) the sequence $\{G_n(\varphi _{\lambda ,n})\}_{n \in \mathbb {N}}$ is uniformly bounded in
  $$\begin{aligned} L^\infty (\Omega \times (0,T);\mathscr {L}^2(U,H)) \cap L^p(\Omega ;L^2(0,T;\mathscr {L}^2(U,V))) \end{aligned}$$
  for any p≥2;
(ii) the sequence $\left\{ \displaystyle \int _0^. G_n(\varphi _{\lambda ,n}(s)) \, {\mathrm d}W(s)\right\} _{n \in \mathbb {N}}$ is uniformly bounded in
  $$\begin{aligned} L^q(\Omega ;W^{k,q}(0,T;H))\cap L^2(\Omega ;H^{k}(0,T;V)) \end{aligned}$$
  for every $k \in (0,\frac{1}{2})$ and q≥2.

#### Proof

A direct application of Assumption (**A3**) yields

$$\begin{aligned} \Vert G_n(\varphi _{\lambda ,n})\Vert _{\mathscr {L}^2(U,V)}^2&\le \Vert G(\varphi _{\lambda ,n})\Vert _{\mathscr {L}^2(U,V)}^2 \le 2L_G^2(1 + \Vert \varphi _{\lambda ,n}\Vert ^2_V) \end{aligned}$$

and thus, using Lemma 3.2, it follows that

$$\begin{aligned} \mathop { \mathbb {E}}\left| \int _0^t\Vert G_n(\varphi _{\lambda ,n}(s))\Vert _{\mathscr {L}^2(U,V)}^2 \, {\mathrm d}s\right| ^\frac{p}{2} \le {C}. \end{aligned}$$

Therefore, the estimate above and similar computations imply

3.22 $$\begin{aligned} \Vert G_n(\varphi _{\lambda ,n})\Vert _{L^\infty (\Omega \times (0,T);\mathscr {L}^2(U,H))\cap L^p(\Omega ;L^2(0,T;\mathscr {L}^2(U,V)))} \le C_\lambda , \end{aligned}$$

where the constant $C_\lambda$ only depends on λ>0. By [20, Lemma 2.1], an estimate on the corresponding Itô integrals holds, namely

3.23 $$\begin{aligned} \left\| \int _0^. G_n(\varphi _{\lambda ,n}(s)) \, {\mathrm d}W(s)\right\| _{L^q(\Omega ;W^{k,q}(0,T;H))\cap L^2(\Omega ;H^{k}(0,T;V))} \le C_{\lambda }, \end{aligned}$$

for every $k\in (0,\frac{1}{2})$ and q≥2. □

Finally, the last estimate follows by a comparison argument.

#### Lemma 3.8

The sequence $\{\varphi _{\lambda , n}\}_{n \in \mathbb {N}}$ is uniformly bounded in $L^p(\Omega ;W^{\alpha , p}(0,T;V^*))$ for any $\alpha \in (0, \frac{1}{2})$ and p≥2.

#### Proof

Let us consider the weak formulation of the discretized problem as an equality in $V^*$, i.e.

$$\begin{aligned} \langle \varphi _{\lambda ,n}(t),\psi \rangle _{V^*,V} + \int _0^t\int _\mathcal {O}\nabla \mu _{\lambda ,n}(s)\cdot \nabla \psi \,{\mathrm d}x \, {\mathrm d}s&= \langle \varphi _{0,n},\psi \rangle _{V^*,V} \\&\quad + \Big (\int _0^tG_n(\varphi _{\lambda ,n}(s))\, {\mathrm d}W(s),\psi \Big )_H \end{aligned}$$

for every $\psi \in V$ such that $\Vert \psi \Vert _V=1$, all t∈[0,T] and $\mathbb {P}$-almost surely. Since

$$\begin{aligned} \left| \int _\mathcal {O}\nabla \mu _{\lambda ,n}(s)\cdot \nabla \psi \,{\mathrm d}x\right| \le \Vert \nabla \mu _{\lambda ,n}(s)\Vert _{\boldsymbol{H}} \end{aligned}$$

and thus, by Lemma 3.6, we arrive at

$$\begin{aligned} \left\| \int _0^t\int _\mathcal {O}\Delta \mu _{\lambda ,n}(s)\,{\mathrm d}x \, {\mathrm d}s\right\| _{L^p(\Omega ;H^1(0,T;V^*))}\le C_\lambda , \end{aligned}$$

where $C_\lambda >0$ depends on λ,p and *T*. Now, recalling

$$\begin{aligned} |\langle \varphi _{0,n},\psi \rangle _{V^*,V}| \le \Vert \varphi _{0,n}\Vert _H \le \Vert \varphi _{0}\Vert _H \end{aligned}$$

and estimate (3.23), we eventually get by comparison and invoking [14, Lemma 2.1]

$$\begin{aligned} \Vert \varphi _{\lambda ,n}\Vert _{L^p(\Omega ;W^{\alpha ,p}(0,T;V^*))} \le C_{4, \lambda } \end{aligned}$$

for some $\alpha = \alpha (p) > \frac{1}{p}$ if p>2 and $\alpha \in (0, \frac{1}{2})$ if p=2. Note that the constant may depend on $\lambda ,\alpha$ and *T*. The proof is complete. □

### Passage to the limit as $n \rightarrow +\infty$

On account of the previous estimates, we now pass to the limit $n\rightarrow +\infty$, keeping λ>0 fixed, by means of a stochastic compactness argument. First of all, let us show that the sequence of laws of $\{\varphi _{\lambda ,n}\}_{n\in \mathbb {N}}$ is tight in a suitable path space. To this end, we recall that the embeddings (see [20, Theorem 2.1])

$$\begin{aligned} L^2(0,T;V)\cap H^\alpha (0,T;V^*)&\hookrightarrow L^2(0,T;H) \\ L^\infty (0,T;H) \cap W^{\alpha , p}(0,T;V^*)&\hookrightarrow C^0([0,T]; H^{-\delta }(\mathcal {O})) \end{aligned}$$

are compact, with the caveat that the second embedding only holds if αp>1, i.e., only if p>2, for some small δ>0.

#### Lemma 3.9

If p>2, then the family of laws of $\{\varphi _{\lambda ,n}\}_{n\in \mathbb {N}}$ is tight in the space $\mathcal {X}:= L^2(0,T;H) \cap C^0([0,T]; H^{-\delta }(\mathcal {O}))$ for any $\delta \in (0, \frac{1}{2})$.

#### Proof

For any R>0, let $B_R$ denote the closed ball of radius *R* in $L^2(0,T;V)\cap H^\alpha (0,T;V^*)$. Then, Markov’s inequality as well as Lemmas 3.2 and 3.8 imply

$$\begin{aligned} \mathbb {P}\{\varphi _{\lambda ,n}\in B_R^c\}&= \mathbb {P}\{\Vert \varphi _{\lambda ,n}\Vert _{L^2(0,T;V)\cap H^\alpha (0,T;V^*)}\ge R\} \\&\le \frac{1}{R^p}\mathop { \mathbb {E}}\Vert \varphi _{\lambda ,n}\Vert _{L^2(0,T;V)\cap H^\alpha (0,T;V^*)}^p \le \frac{C}{R^p}. \end{aligned}$$

In particular, this yields

$$\begin{aligned} \lim \limits _{R\rightarrow +\infty }\sup _{n\in \mathbb {N}}\mathbb {P}\{\varphi _{\lambda ,n}\in B_R^c\} = 0. \end{aligned}$$

This shows that the family of laws $(\varphi _{\lambda ,n})_{n\in \mathbb {N}}$ on $L^2(0,T;H)$ is tight. An analogous argument can be carried out for the second embedding. □

Let us now set

$$\begin{aligned} G_n(\varphi _{\lambda ,n})\cdot W := \int _0^. G_n(\varphi _{\lambda ,n}(s)){\mathrm d}W(s). \end{aligned}$$

In the spirit of Lemma 3.9, using (3.23) and [20, Theorem 2.2] we exploit the compact embedding

$$ W^{k, q}(0,T; H) \hookrightarrow C^0([0,T]; V^*) $$

holding whenever kq>1 (here, we are free to choose q≥2 and $k > \frac{1}{q}$). Hence, we immediately have

#### Lemma 3.10

The family of laws of $\{G_n(\varphi _{\lambda ,n})\cdot W\}_{n \in \mathbb {N}}$ is tight in the space $C^0([0,T]; V^*)$.

By identifying *W* with a constant sequence of random variables $\{W_n\}_{n \in \mathbb {N}}$ with values in $C^0([0,T];U_0)$, we also get that

#### Lemma 3.11

The family of laws of $\{W_n\}_{n \in \mathbb {N}}$ is tight in the space $C^0([0,T]; U_0)$.

Finally, the sequence of initial conditions is clearly tight in $V^*$ as $H \hookrightarrow V^*$ compactly. Namely, we have

#### Lemma 3.12

The family of laws of $\{\varphi _{0,n}\}_{n \in \mathbb {N}}$ is tight in $V^*$.

Collecting Lemmas 3.9-3.12, we obtain that the family of laws

$$\begin{aligned} \{\varphi _{\lambda ,n},\, G_n(\varphi _{\lambda ,n})\cdot W,\, W_n,\, \varphi _{0,n}\}_{n\in \mathbb {N}} \end{aligned}$$

is tight in the space

$$\begin{aligned} \mathcal {X} \times C^0([0,T]; V^*) \times C^0([0,T];U_0) \times V^*. \end{aligned}$$

Owing to the Prokhorov and Skorokhod theorems (see [32, Theorem 2.7] and [39, Theorem 1.10.4, Addendum 1.10.5]), there exists a probability space $(\widetilde{\Omega }, \widetilde{\mathscr {F}},\widetilde{\mathbb {P}})$ and a sequence of random variables $X_n: (\widetilde{\Omega }, \widetilde{\mathscr {F}})\rightarrow (\Omega , \mathscr {F})$ such that the law of $X_n$ is $\mathbb {P}$ for every $n \in \mathbb {N}$, namely $\widetilde{\mathbb {P}}\circ X_n^{-1} = \mathbb {P}$ (so that composition with $X_n$ preserves laws), and the following convergences hold

$$\begin{aligned} \widetilde{\varphi }_{\lambda ,n} := \varphi _{\lambda ,n}\circ X_n \rightarrow \widetilde{\varphi }_\lambda&\quad \text { in }\mathcal {X}, \, \widetilde{\mathbb {P}}\text {-a.s.,}\\ \widetilde{I}_{\lambda ,n} := (G_n(\varphi _{\lambda ,n})\cdot W)\circ X_n \rightarrow \widetilde{I}_\lambda&\quad \text { in }C^0([0,T];V^*), \, \widetilde{\mathbb {P}}\text {-a.s.,}\\ \widetilde{W}_n:= W\circ X_n \rightarrow \widetilde{W}&\quad \text { in }C^0([0,T];U_0),\, \widetilde{\mathbb {P}}\text {-a.s.,}\\ \widetilde{\varphi }_{0,n} := \varphi _{0,n} \circ X_n \rightarrow \widetilde{\varphi }_{0}&\quad \text { in }V^*,\, \widetilde{\mathbb {P}}\text {-a.s.,} \end{aligned}$$

and, in turn, exploiting the Vitali convergence theorem, as well as the Banach–Alaoglu theorem and reflexivity, we conclude that

$$\begin{aligned} \widetilde{\varphi }_{\lambda ,n} \rightarrow \widetilde{\varphi }_\lambda&\quad \text { in }L^q(\widetilde{\Omega };L^2(0,T;H) \cap C^0([0,T]; H^{-\delta }(\mathcal {O}))\;\;\forall \, q<p,\\ \widetilde{\varphi }_{\lambda ,n} \overset{*}{\rightharpoonup }\ \widetilde{\varphi }_\lambda&\quad \text { in }L^p_w(\widetilde{\Omega }; L^\infty (0,T;H)) \cap L^p(\widetilde{\Omega };H^\alpha (0,T;V^*)),\\ \widetilde{\varphi }_{\lambda ,n} \rightharpoonup \widetilde{\varphi }_\lambda&\quad \text { in }L^p(\widetilde{\Omega };L^2(0,T;V)), \\ \widetilde{\mu }_{\lambda ,n} := \mu _{\lambda ,n}\circ X_n \rightharpoonup \widetilde{\mu }_\lambda&\quad \text { in }L^{p}(\widetilde{\Omega };L^2(0,T;V)), \\ \widetilde{I}_{\lambda ,n} \rightarrow \widetilde{I}_\lambda&\quad \text { in }L^q(\widetilde{\Omega };C^0([0,T];V^*))\;\;\forall \, q<p,\\ \widetilde{W}_n\rightarrow \widetilde{W}&\quad \text { in }L^q(\widetilde{\Omega };C^0([0,T];U_0))\;\;\forall \, q<p, \\ \widetilde{\varphi }_{0,n}\rightarrow \widetilde{\varphi }_{0}&\quad \text { in }L^q(\widetilde{\Omega };V^*)\;\;\forall \, q<p, \end{aligned}$$

for some limiting measurable processes enjoying the following regularity properties:

$$\begin{aligned} \widetilde{\varphi }_\lambda&\in L^p_w(\widetilde{\Omega };L^\infty (0,T;H))\cap L^p(\widetilde{\Omega };H^\alpha (0,T;V^*))\cap L^p(\widetilde{\Omega };L^2(0,T;V) \\&\cap C^0([0,T]; H^{-\delta }(\mathcal {O})),\\ \widetilde{\mu }_\lambda&\in L^p(\widetilde{\Omega };L^2(0,T;V)),\\ \widetilde{I}_\lambda&\in L^p(\widetilde{\Omega };C^0([0,T];V^*)), \\ \widetilde{W}&\in L^p(\widetilde{\Omega };C^0([0,T];U)), \\ \widetilde{\varphi }_0&\in L^p(\widetilde{\Omega }; H). \end{aligned}$$

Observe that from the proof of Skorokhod’s theorem, the probability space $(\widetilde{\Omega },\widetilde{\mathscr {F}},\widetilde{\mathbb {P}})$ can be made independent of λ.

### Identification of the limit solution

Before moving to proving that the limiting quantities satisfy the regularized problem (3.4), we deduce the behavior of the nonlinearities of the problem. Using the strong convergence of

$$ \widetilde{\varphi }_{\lambda ,n} \rightarrow \widetilde{\varphi }_\lambda \quad \text { in }L^q(\widetilde{\Omega };L^2(0,T;H)) $$

for q<p, jointly with the Lipschitz-continuity of $F_\lambda ^\prime$ (that is uniform in *n*), we immediately have

$$\begin{aligned} F^\prime _\lambda (\widetilde{\varphi }_{\lambda ,n}) \rightarrow F^\prime _\lambda (\widetilde{\varphi }_{\lambda })\quad \text { in } L^q(\widetilde{\Omega };L^2(0,T;H)) \end{aligned}$$

for q<p. Similarly, since $G_n$ is Lipschitz-continuous, and on account of

$$\begin{aligned}  &   \Vert G_n(\widetilde{\varphi }_{\lambda ,n}) - G(\widetilde{\varphi }_{\lambda })\Vert _{L^q(\widetilde{\Omega };L^2(0,T;\mathscr {L}^2(U,H)))}\\  &   \quad \quad \le \Vert G_n(\widetilde{\varphi }_{\lambda ,n}) - G_n(\widetilde{\varphi }_{\lambda })\Vert _{L^q(\widetilde{\Omega };L^2(0,T;\mathscr {L}^2(U,H)))}\\  &   \quad \quad \quad \quad \quad \quad \quad \quad + \Vert G_n(\widetilde{\varphi }_{\lambda }) - G(\widetilde{\varphi }_{\lambda })\Vert _{L^q(\widetilde{\Omega };L^2(0,T;\mathscr {L}^2(U,H)))} \rightarrow 0 \end{aligned}$$

as $n \rightarrow +\infty$, it follows that

$$\begin{aligned} G_n(\widetilde{\varphi }_{\lambda ,n}) \rightarrow G(\widetilde{\varphi }_{\lambda })\quad \text { in }L^q(\widetilde{\Omega };L^2(0,T;\mathscr {L}^2(U,H))) \end{aligned}$$

for all q<p. Next, we identify the limit of the stochastic integral sequence. To this end, we employ a standard method illustrated, for instance, in [8, Section 8.4]. Consider the family of filtrations on $(\widetilde{\Omega }, \widetilde{\mathscr {F}}, \widetilde{\mathbb {P}})$ generated by the Galerkin solutions, that is,

$$ \widetilde{\mathscr {F}}_{\lambda ,n,t} := \sigma \left\{ \widetilde{\varphi }_{\lambda ,n}(s),\, \widetilde{I}_{\lambda ,n}(s),\widetilde{W}_{n}(s),\,\widetilde{\varphi }_{0,n}, \, s \in [0,t]\right\} , $$

for any t≥0, $n \in \mathbb {N}$ and $\lambda \in (0,1)$. By definition, we have that the processes $\widetilde{W}_n$ are clearly adapted to $\widetilde{\mathscr {F}}_{\lambda , n, t}$. In particular, by preservation of laws and the definitions of Wiener process and stochastic integral, we readily have that $\widetilde{W}_{n}$ is a $Q_0$-Wiener process on some larger space $U_0 \supset U$ and, correspondingly,

$$ \widetilde{I}_{\lambda , n} = \int _0^t G_{n}(\widetilde{\varphi }_{\lambda ,n}(\tau )) \, \textrm{d}\widetilde{W}_{n}(\tau ), $$

is a martingale with values in $V^*$. Similarly, by considering the limit processes, we can define the family of increasing filtrations

$$ \widetilde{\mathscr {F}}_{\lambda ,t} := \sigma \left\{ \widetilde{\varphi }_{\lambda }(s),\, \widetilde{I}_{\lambda }(s), \widetilde{W}(s), \,\widetilde{\varphi }_0,\, s \in [0,t]\right\} . $$

Let now t>0, s∈[0,t] and consider the path spaces

$$\begin{aligned} \mathcal {X}_{s}&:= L^2(0,s;H) \cap C^0([0,s];H^{-\delta }(\mathcal {O})), \\ \mathcal {Z}_s&:= \mathcal {X}_s \times C^0([0,s]; V^*) \times C^0([0,s]; U^0) \times V^*. \end{aligned}$$

In what follows, we denote by ψ an arbitrary but fixed element of $C_\text {b}(\mathcal {Z}_s)$, namely, a continuous and bounded function on $\mathcal {Z}_s$ with values in $\mathbb {R}$. By definition of martingale (or Wiener process), we have

3.24 $$\begin{aligned} \widetilde{\mathbb {E}}\left[ \left( \widetilde{W}_{n}(t) -\widetilde{W}_{n}(s) \right) \psi \left( \widetilde{\varphi }_{\lambda ,n},\, \widetilde{I}_{\lambda ,n},\, \widetilde{W}_{n},\,\widetilde{\varphi }_{0,n} \right) \right] = 0 \end{aligned}$$

for any bounded and continuous function $\psi \in C_\text {b}(\mathcal {Z}_s)$. Of course, the arguments of ψ are intended to be restricted over [0, *s*]. Letting $n \rightarrow +\infty$ in (3.24), an application of the dominated convergence theorem, owing to the proven convergences and the properties of ψ, entails

3.25 $$\begin{aligned} \widetilde{\mathbb {E}}\left[ \left( \widetilde{W}(t) -\widetilde{W}(s) \right) \psi \left( \widetilde{\varphi }_{\lambda },\, \widetilde{I}_{\lambda },\,\widetilde{W},\,\widetilde{\varphi }_0 \right) \right] = 0, \end{aligned}$$

which implies that $\widetilde{W}$ is a $U_0$-valued $(\widetilde{\mathscr {F}}_{\lambda ,t})_t$-adapted martingale. In order to show that $\widetilde{W}$ is indeed a Wiener process, we resort to the characterization of *Q*-Wiener processes given by [8, Theorem 4.6]. To this end, notice that (3.25) means that, for every $v, w \in {U_0}$

$$\begin{aligned}&\widetilde{\mathbb {E}}\left[ \left( \left( \widetilde{W}_{n}(t), v \right) _{U_0} \left( \widetilde{W}_{n}(t), w \right) _{U_0} -\left( \widetilde{W}_{n}(s), v \right) _{U_0} \left( \widetilde{W}_{n}(s), w \right) _{U_0} - (t-s)\left( Q_0v,w\right) _{U_0}\right) \right. \\&\quad \left. \psi \left( \widetilde{\varphi }_{\lambda ,n},\, \widetilde{I}_{\lambda ,n},\, \widetilde{W}_{n},\,\widetilde{\varphi }_{0,n} \right) \right] = 0, \end{aligned}$$

and using once more the dominated convergence theorem, we get

$$\begin{aligned}&\widetilde{\mathbb {E}}\left[ \left( \left( \widetilde{W}(t), v \right) _{U_0} \left( \widetilde{W}(t), w \right) _{U_0} -\left( \widetilde{W}(s), v \right) _{U_0} \left( \widetilde{W}(s), w \right) _{U_0} - (t-s)\left( Q_0v,w\right) _{U_0}\right) \right. \\&\quad \left. \psi \left( \widetilde{\varphi }_{\lambda },\, \widetilde{I}_{\lambda },\, \widetilde{W},\,\widetilde{\varphi }_{0} \right) \right] = 0, \end{aligned}$$

namely the quadratic variation is exactly

$$\begin{aligned} \big \langle \!\big \langle {\widetilde{W}}\big \rangle \!\big \rangle (t) = t{Q_0}, \qquad t \in [0,T], \end{aligned}$$

which is enough to conclude that $\widetilde{W}$ is a $Q_0$-Wiener process, adapted to $(\widetilde{\mathscr {F}}_{\lambda ,t})_t$, owing to [8, Theorem 4.6]. In a similar way, we address the stochastic integrals. Arguing as in (3.24)-(3.25), we conclude that $\widetilde{I}_{\lambda }$ is a $V^*$-valued martingale. As far as the quadratic variations are concerned, an application of [8, Theorem 4.27] yields

$$\begin{aligned} \big \langle \!\big \langle \widetilde{I}_{\lambda ,n}\big \rangle \!\big \rangle (t) = \int _0^t G_{n}(\widetilde{\varphi }_{\lambda ,n}(\tau )) \circ G_{n}(\widetilde{\varphi }_{\lambda ,n}(\tau ))^*\, \textrm{d}\tau , \end{aligned}$$

for every t∈[0,T]. Let v,w∈V be fixed. Then

$$\begin{aligned}  &   \widetilde{\mathbb {E}}\left[ \left( \left\langle \widetilde{I}_{\lambda ,n}(t), v \right\rangle _{V^*, V}\left\langle \widetilde{I}_{\lambda ,n}(t), w \right\rangle _{V^*, V} - \left\langle \widetilde{I}_{\lambda ,n}(s), v \right\rangle _{V^*, V}\left\langle \widetilde{I}_{\lambda ,n}(s), w \right\rangle _{V^*, V} \right. \right. \\    &   \left. \left. -\int _0^t (G_{n}(\widetilde{\varphi }_{\lambda ,n}(\tau )) \circ G_{n}(\widetilde{\varphi }_{\lambda ,n}(\tau ))^*v, w)_H \, \textrm{d}\tau \right) \psi \left( \widetilde{\varphi }_{\lambda ,n},\,\widetilde{I}_{\lambda ,n},\, \widetilde{W}_{n} ,\,\widetilde{\varphi }_{0,n}\right) \right] = 0, \end{aligned}$$

and, as $n \rightarrow +\infty$, the dominated convergence theorem implies that

$$\begin{aligned}  &   \widetilde{\mathbb {E}}\left[ \left( \left\langle \widetilde{I}_{\lambda }(t), v \right\rangle _{V^*, V}\left\langle \widetilde{I}_{\lambda }(t), w \right\rangle _{V^*, V} - \left\langle \widetilde{I}_{\lambda }(s), v \right\rangle _{V^*, V}\left\langle \widetilde{I}_{\lambda }(s), w \right\rangle _{V^*, V} \right. \right. \\    &   \quad \quad \quad \quad \left. \left. - \int _0^t (G(\widetilde{\varphi }_{\lambda }(\tau )) \circ G(\widetilde{\varphi }_{\lambda }(\tau ))^*v, w)_H \, \textrm{d}\tau \right) \psi \left( \widetilde{\varphi }_{\lambda },\,\widetilde{I}_{\lambda },\, \widetilde{W} ,\,\widetilde{\varphi }_{0}\right) \right] = 0, \end{aligned}$$

namely, we have shown that the quadratic variation of $\widetilde{I}_{\lambda }$ is

$$ \big \langle \!\big \langle \widetilde{I}_{\lambda } \big \rangle \!\big \rangle (t) = \int _0^t G(\widetilde{\varphi }_{\lambda }(\tau )) \circ G(\widetilde{\varphi }_{\lambda }(\tau ))^* \, \textrm{d}\tau , \qquad t \in [0,T]. $$

In particular, we are left to show that $\widetilde{I}_{\lambda }$ can be identified with the process

$$ \widetilde{M}_{\lambda }(t) := \int _0^t {G(\widetilde{\varphi }_{\lambda }(\tau ))}\, \textrm{d}{\widetilde{W}}(\tau ), $$

which is a $V^*$-valued $(\widetilde{\mathscr {F}}_{\lambda ,t})_t$-adapted martingale having the same quadratic variation of $\widetilde{I}_{\lambda }$. By [34, Theorem 3.2], we can write

3.26 $$\begin{aligned} \begin{aligned} \big \langle \!\big \langle \widetilde{M}_{\lambda } - \widetilde{I}_{\lambda } \big \rangle \!\big \rangle&= \big \langle \!\big \langle \widetilde{M}_{\lambda } \big \rangle \!\big \rangle + \big \langle \!\big \langle \widetilde{I}_{\lambda } \big \rangle \!\big \rangle -2 \big \langle \!\big \langle \widetilde{M}_{\lambda }, \widetilde{I}_{\lambda } \big \rangle \!\big \rangle \\&= 2\int _0^\cdot G(\widetilde{\varphi }_{\lambda }(\tau )) \circ G(\widetilde{\varphi }_{\lambda }(\tau ))^*\, \textrm{d}\tau - 2\int _0^\cdot G(\widetilde{\varphi }_{\lambda }(\tau )) \, \textrm{d}\big \langle \!\big \langle \widetilde{W}, \widetilde{I}_{\lambda } \big \rangle \!\big \rangle (\tau ). \end{aligned} \end{aligned}$$

Moreover, still by [34, Theorem 3.2], we have that the cross quadratic variation satisfies

$$ \begin{aligned} \big \langle \!\big \langle \widetilde{I}_{\lambda ,n}, \widetilde{W}_{n} \big \rangle \!\big \rangle&= \int _0^\cdot G_{n}(\widetilde{\varphi }_{\lambda ,n}(\tau )) \circ \iota ^{-1}_1 \, \textrm{d}\big \langle \!\big \langle \widetilde{W}_{n}, \widetilde{W}_{n}\big \rangle \!\big \rangle (\tau ) \\&= \int _0^\cdot G_{n}(\widetilde{\varphi }_{\lambda ,n}(\tau )) \circ \iota ^{-1} \circ Q_0 \, \textrm{d}\tau \\&= \int _0^\cdot G_{n}(\widetilde{\varphi }_{\lambda ,n}(\tau )) \circ \iota ^{-1} \circ \iota \circ \iota ^* \, \textrm{d}\tau \\&= \int _0^\cdot G_{n}(\widetilde{\varphi }_{\lambda ,n}(\tau )) \circ \iota ^* \, \textrm{d}\tau , \end{aligned} $$

where we also used the fact that $Q_0 = \iota _1 \circ \iota ^*_1$, where $\iota : U \rightarrow U_0$ is the classical Hilbert-Schmidt embedding. This implies that

$$ \big \langle \!\big \langle \widetilde{W}_{n}, \widetilde{I}_{\lambda ,n} \big \rangle \!\big \rangle = \int _0^\cdot \iota \circ G_{n}(\widetilde{\varphi }_{\lambda ,n}(\tau ))^* \, \textrm{d}\tau . $$

A further application of the dominated convergence theorem entails that, as $n \rightarrow +\infty$,

3.27 $$\begin{aligned} \big \langle \!\big \langle \widetilde{W}, \widetilde{I}_{\lambda } \big \rangle \!\big \rangle = \int _0^\cdot \iota \circ G(\widetilde{\varphi }_{\lambda }(\tau ))^* \, \textrm{d}\tau . \end{aligned}$$

The identification follows injecting (3.27) in (3.26). Finally, the proven convergences are enough to pass to the limit in the Galerkin problem and retrieve that the limit processes are indeed a martingale solution of (3.4).

### Uniform estimates with respect to λ

Here, we want to show further estimates, now uniform in the Yosida parameter λ>0. The symbol *C* (possibly numbered) denotes a positive constant, always independent of λ, but possibly dependent on other structural quantities of the problem, and may change from line to line. First of all, from the proof of Lemma 3.2 we observe that the constant $C_1$ in estimate (3.14) is already independent of λ.

#### Lemma 3.13

The family $\{\widetilde{\varphi }_\lambda \}_{\lambda > 0}$ is uniformly bounded in $L^p(\widetilde{\Omega };L^\infty (0,T;H)) \cap L^p(\widetilde{\Omega };L^2(0,T;V))$ for all p≥2.

#### Proof

By using Lemma 3.2, lower semicontinuity and preservation of laws of $X_n$, we obtain

$$\begin{aligned} \Vert \widetilde{\varphi }_{\lambda }\Vert _{L^p(\widetilde{\Omega };L^\infty (0,T;H))} + \Vert \widetilde{\varphi }_{\lambda }\Vert _{L^p(\widetilde{\Omega };L^2(0,T;V))} \le C_1, \end{aligned}$$

and the proof is complete. □

Next, the key energy inequality must be revisited in order to obtain independence from λ. In particular, we shall deduce the following estimate.

#### Lemma 3.14

It holds that

(i) the family $\{\widetilde{\mu }_\lambda \}_{\lambda > 0}$ is uniformly bounded in $L^\frac{p}{2}(\widetilde{\Omega };L^2(0,T;V))$;
(ii) the family $\{\nabla \widetilde{\mu }_\lambda \}_{\lambda > 0}$ is uniformly bounded in $L^p(\widetilde{\Omega };L^2(0,T;{\boldsymbol{H}}))$;
(iii) the family $\{\Psi _\lambda (\widetilde{\varphi }_\lambda )\}_{\lambda >0}$ is uniformly bounded in $L^\frac{p}{2}(\widetilde{\Omega };L^\infty (0,T;L^1(\mathcal {O})))$;

for all p≥2.

#### Proof

Let us start back from

3.28 $$\begin{aligned}&\mathcal {E}_\lambda (\varphi _{\lambda ,n}(t)) + \int _0^t\int _\mathcal {O}|\nabla \mu _{\lambda ,n}(s)|^2\,{\mathrm d}x\,{\mathrm d}s\nonumber \\&\quad = \mathcal {E}_\lambda (\varphi _{0,n}) + \int _0^t\Big (\mu _{\lambda ,n}(s),G_n(\varphi _{\lambda ,n}(s))\,{\mathrm d}W(s)\Big )_H \nonumber \\&\qquad + \frac{1}{2}\int _0^t\sum _{k=0}^{+\infty }\left[ \frac{1}{2}\Lambda (G_n(\varphi _{\lambda ,n}(s))[u_k], G_n(\varphi _{\lambda ,n}(s))[u_k]) \right. \nonumber \\&\qquad \left. +\int _\mathcal {O}F^{\prime \prime }_\lambda (\varphi _{\lambda ,n}(s))|G_n(\varphi _{\lambda ,n}(s))[u_k]|^2\,{\mathrm d}x \right] \,{\mathrm d}s. \end{aligned}$$

In order to proceed, it will be much more convenient to recast (3.28) with “lower-order” terms to the right hand side, namely

3.29 $$\begin{aligned}&\int _\mathcal {O}\Psi _\lambda (\varphi _{\lambda ,n}(t)) \, {\mathrm d}x+ \int _0^t\int _\mathcal {O}|\nabla \mu _{\lambda ,n}(s)|^2\,{\mathrm d}x\,{\mathrm d}s\nonumber \\&\quad = - \Lambda (\varphi _{\lambda ,n}(t), \varphi _{\lambda ,n}(t)) + \dfrac{\gamma }{2}\int _\mathcal {O}\varphi _{\lambda ,n}^2(t) \, {\mathrm d}x \nonumber \\  &\qquad +\mathcal {E}_\lambda (\varphi _{0,n}) + \int _0^t\Big (\mu _{\lambda ,n}(s),G_n(\varphi _{\lambda ,n}(s))\,{\mathrm d}W(s)\Big )_H \nonumber \\  &\qquad + \frac{1}{2}\int _0^t\sum _{k=0}^{+\infty }\left[ \frac{1}{2}\Lambda (G_n(\varphi _{\lambda ,n}(s))[u_k], G_n(\varphi _{\lambda ,n}(s))[u_k]) \right. \nonumber \\&\qquad \left. +\int _\mathcal {O}F^{\prime \prime }_\lambda (\varphi _{\lambda ,n}(s))|G_n(\varphi _{\lambda ,n}(s))[u_k]|^2\,{\mathrm d}x \right] \,{\mathrm d}s. \end{aligned}$$

Firstly, observe that the terms in Λ (i.e., the first and fifth terms on the right hand side) can be bounded independently of λ through Lemma 3.3 (as in the proof of Lemma 3.6). Then, we consider the initial energy. It holds

$$ \begin{aligned} \mathcal {E}_\lambda ({\varphi _{0,n}}) = \dfrac{1}{4}\Lambda ({\varphi _{0,n}}, {\varphi _{0,n}}) + \int _\mathcal {O}\Psi _\lambda (\varphi _{0,n}(x)) - \dfrac{\gamma }{2}\varphi _{0,n}^2(x)\, {\mathrm d}x + C, \end{aligned} $$

where $C = C_\Lambda + F(0)|\mathcal {O}|$ is independent of λ. By Lemma 3.3 and the convergence $\varphi _{0,n} \rightarrow \varphi _0$ in *H* with $\Vert \varphi _{0,n}\Vert ^2_H \le \Vert \varphi _0\Vert ^2_H$ for all $n \in \mathbb {N}$, $\mathbb {P}$-almost surely, we get

$$ \begin{aligned} \mathcal {E}_\lambda ({\varphi _{0,n}})&\le C\left( 1 + \int _\mathcal {O}\Psi _\lambda (\varphi _{0,n}(x)) \, {\mathrm d}x + \Vert \varphi _0\Vert ^2_H\right) \end{aligned} $$

$\mathbb {P}$-almost surely for a constant *C* independent of λ and *n*. Now, collecting all the above controls, raising the result to the power $\frac{p}{2}$, taking supremums and $\mathbb {P}$-expectations we arrive at

$$\begin{aligned}&\mathop { \mathbb {E}}\sup _{\tau \in [0,\,t]}\mathcal \Vert \Psi _\lambda (\varphi _{\lambda , n}(\tau ))\Vert ^\frac{p}{2}_{L^1(\mathcal {O})} + \mathop { \mathbb {E}}\left| \int _0^t \Vert \nabla \mu _{\lambda ,n}(\tau )\Vert _{{\boldsymbol{H}}}^2 \, {\mathrm d}\tau \right| ^\frac{p}{2} \\  &\quad \le C\left[ 1+\mathop { \mathbb {E}}\Vert \varphi _{0}\Vert _H^{p} + {\mathop { \mathbb {E}}\Vert \Psi _\lambda (\varphi _{0,n})\Vert _{L^1(\mathcal {O})}^\frac{p}{2}}\right] \\&\qquad + C\left[ \mathop { \mathbb {E}}\left| \int _0^t\sum _{k=0}^{+\infty }\int _\mathcal {O}|F_\lambda ^{\prime \prime }(\varphi _{\lambda ,n})||G_n(\varphi _{\lambda ,n})[u_k]|^2\,{\mathrm d}x \, {\mathrm d}s\right| ^{\frac{p}{2}}\right. \\&\qquad \left. + \mathop { \mathbb {E}}\sup _{\tau \in [0,\,t]}\left| \int _0^\tau \Big (\mu _{\lambda ,n}(s),G_n(\varphi _{\lambda ,n}(s))\,{\mathrm d}W(s)\Big )_H\right| ^\frac{p}{2} \right] . \end{aligned}$$

Using the Burkholder-Davis-Gundy inequality, as in the proof of Lemma 3.6 (concatenating, in particular (3.18), ignoring the last line, and (3.19) replacing, $\frac{p}{2}$ with $\frac{p}{4}$), we can bound the last term and obtain an additional term depending on the spatial average of the chemical potential, i.e.,

3.30 $$\begin{aligned}&\mathop { \mathbb {E}}\sup _{\tau \in [0,\,t]}\mathcal \Vert \Psi _\lambda (\varphi _{\lambda , n}(\tau ))\Vert ^\frac{p}{2}_{L^1(\mathcal {O})} + \mathop { \mathbb {E}}\left| \int _0^t \Vert \nabla \mu _{\lambda ,n}(\tau )\Vert _{{\boldsymbol{H}}}^2 \, {\mathrm d}\tau \right| ^\frac{p}{2} \nonumber \\&\quad \le C\left[ 1+\mathop { \mathbb {E}}\Vert \varphi _{0}\Vert _H^{p} + {\mathop { \mathbb {E}}\Vert \Psi _\lambda (\varphi _{0,n})\Vert _{L^1(\mathcal {O})}^\frac{p}{2}}\right] \nonumber \\&\qquad + C\left[ \mathop { \mathbb {E}}\left| \int _0^t\sum _{k=0}^{+\infty }\int _\mathcal {O}|F_\lambda ^{\prime \prime }(\varphi _{\lambda ,n})||G_n(\varphi _{\lambda ,n})[u_k]|^2\,{\mathrm d}x \, {\mathrm d}s\right| ^{\frac{p}{2}} +\mathop { \mathbb {E}}\left| \int _0^t |\overline{\mu _{\lambda , n}(s)}|^2 \, {\mathrm d}s \right| ^{\frac{p}{4}} \right] \end{aligned}$$

Finally, we observe that, given the growth condition Assumption (**A2**)

$$ \begin{aligned} \left| |\mathcal {O}|\overline{\mu _{\lambda ,n}} \right|&= \left| (\mu _{\lambda ,n}, 1)_H \right| \\&\le |(a\varphi _{\lambda ,n}, 1)_H| + |(K * \varphi _{\lambda ,n}, 1)_H| + |(\Psi ^\prime _\lambda (\varphi _{\lambda ,n}), 1)_H| + \gamma (\varphi _{\lambda ,n}, 1)_H \\&\le C\left[ 1 + \Vert \varphi _{\lambda ,n}\Vert _H + \int _\mathcal {O}\Psi (J_\lambda (\varphi _{\lambda ,n})) \, {\mathrm d}x\right] \\&\le C\left[ 1 + \Vert \varphi _{\lambda ,n}\Vert _H + \int _\mathcal {O}\Psi _\lambda (\varphi _{\lambda ,n}) \, {\mathrm d}x\right] \end{aligned} $$

since the general theory (see [2]) implies $\Psi \circ J_\lambda \le \Psi _\lambda$ in $\mathbb {R}$. Therefore, we arrive at

3.31 $$\begin{aligned} \mathop { \mathbb {E}}\sup _{\tau \in [0,\,t]}|\overline{\mu _{\lambda ,n}}(\tau )|^\frac{p}{2} \le C\left[ 1 +\mathop { \mathbb {E}}\sup _{\tau \in [0,\,t]}\Vert \Psi _\lambda (\varphi _{\lambda ,n}(\tau ))\Vert _{L^1(\mathcal {O})}^\frac{p}{2} \right] . \end{aligned}$$

Hence, multiplying (3.31) for a sufficiently small constant, we can sum (3.31) to (3.30) and conclude, exploiting also the equality of laws preserved by $X_n$,

3.32 $$\begin{aligned}&\widetilde{\mathbb {E}}\sup _{\tau \in [0,\,t]}\mathcal \Vert \Psi _\lambda (\widetilde{\varphi }_{\lambda , n}(\tau ))\Vert ^\frac{p}{2}_{L^1(\mathcal {O})} + \widetilde{\mathbb {E}}\sup _{\tau \in [0,\,t]}|\overline{\widetilde{\mu }_{\lambda ,n}(\tau )}|^\frac{p}{2} + \widetilde{\mathbb {E}}\left| \int _0^t \Vert \nabla \widetilde{\mu }_{\lambda ,n}(\tau )\Vert _{{\boldsymbol{H}}}^2 \, {\mathrm d}\tau \right| ^\frac{p}{2} \nonumber \\  &\quad \le C\left[ 1+\widetilde{\mathbb {E}}\Vert \widetilde{\varphi }_{0}\Vert _H^{p} + \widetilde{\mathbb {E}}\Vert \Psi _\lambda ({\widetilde{\varphi }_{0,n}})\Vert _{L^1(\mathcal {O})}^\frac{p}{2} + \widetilde{\mathbb {E}}\left| \int _0^t |\overline{\widetilde{\mu }_{\lambda , n}(s)}|^2 \, {\mathrm d}s \right| ^{\frac{p}{4}} \right. \nonumber \\&\qquad \left. + \widetilde{\mathbb {E}}\left| \int _0^t\sum _{k=0}^{+\infty }\int _\mathcal {O}|F_\lambda ^{\prime \prime }(\widetilde{\varphi }_{\lambda ,n}(s))||G_n(\widetilde{\varphi }_{\lambda ,n}(s))[u_k]|^2\,{\mathrm d}x \, {\mathrm d}s\right| ^{\frac{p}{2}} \right] . \end{aligned}$$

Now, we would like to let $n\rightarrow +\infty$ in (3.32). By weak lower semicontinuity of the norms, given the weak convergences proven in the previous subsection, it is immediate to pass to the limit to the second and third terms to the left hand side. As ${\widetilde{\varphi }_{0,n}} \rightarrow {\widetilde{\varphi }_{0}}$ in $L^\frac{p}{2}(\widetilde{\Omega }; H)$, it follows that $\Psi _\lambda ({\widetilde{\varphi }_{0,n}}) \rightarrow \Psi _\lambda ({\widetilde{\varphi }_{0}})$ in $L^\frac{p}{2}(\widetilde{\Omega };L^1(\mathcal {O}))$ as $n\rightarrow +\infty$ by the dominated convergence theorem. Moreover, due to the strong convergences

$$\begin{aligned} G_n(\widetilde{\varphi }_{\lambda ,n}) \rightarrow G(\widetilde{\varphi }_{\lambda })&\quad \text { in }L^q(\widetilde{\Omega };L^2(0,T;\mathscr {L}^2(U,H))),\\ \widetilde{\varphi }_{\lambda ,n} \rightarrow \widetilde{\varphi }_\lambda&\quad \text { in }L^q(\widetilde{\Omega };L^2(0,T;H)) \end{aligned}$$

for all q<p, we deduce that, up to subsequences that we do not relabel for readability purposes,

$$\begin{aligned} |F_\lambda ^{\prime \prime }(\widetilde{\varphi }_{\lambda ,n})||G_n(\widetilde{\varphi }_{\lambda ,n})[u_k]|^2 \rightarrow |F_\lambda ^{\prime \prime }(\widetilde{\varphi }_{\lambda })||G(\widetilde{\varphi }_{\lambda })[u_k]|^2\quad \text {a.e. in }\widetilde{\Omega }\times (0,T)\times \mathcal {O}\end{aligned}$$

for all $k\in \mathbb {N}$. Noticing that $|F^{\prime \prime }_\lambda | \le C_\lambda$ for some constant $C_\lambda >0$, we infer that

$$\begin{aligned} \sum _{k=0}^{+\infty }\int _\mathcal {O}|F_\lambda ^{\prime \prime }(\widetilde{\varphi }_{\lambda ,n})||G_n(\widetilde{\varphi }_{\lambda ,n})[u_k]|^2{\mathrm d}x \le C_\lambda \Vert G_n(\widetilde{\varphi }_{\lambda ,n})\Vert _{\mathscr {L}^2(U,H)}^2 \le C_\lambda \end{aligned}$$

owing to Assumption (**A3**). Hence, given Lemma 3.13, we can apply the dominated convergence theorem to conclude

$$\begin{aligned}&\widetilde{\mathop { \mathbb {E}}}\left( \int _0^t\sum _{k=0}^{+\infty }\int _\mathcal {O}|F_\lambda ^{\prime \prime }(\widetilde{\varphi }_{\lambda ,n})||G_n(\widetilde{\varphi }_{\lambda ,n})[u_k]|^2\,{\mathrm d}x \, {\mathrm d}s\right) ^{\frac{p}{2}} \\&\quad \rightarrow \widetilde{\mathop { \mathbb {E}}}\left( \int _0^t\sum _{k=0}^{+\infty }\int _\mathcal {O}|F_\lambda ^{\prime \prime }(\widetilde{\varphi }_{\lambda })||G(\widetilde{\varphi }_{\lambda })[u_k]|^2\,{\mathrm d}x \, {\mathrm d}s\right) ^{\frac{p}{2}} \end{aligned}$$

as $n\rightarrow +\infty$. We are now in a position to pass to the limit as $n \rightarrow +\infty$ in (3.32), yielding

3.33 $$\begin{aligned}&\widetilde{\mathbb {E}}\sup _{\tau \in [0,\,t]}\mathcal \Vert \Psi _\lambda (\widetilde{\varphi }_{\lambda }(\tau ))\Vert ^\frac{p}{2}_{L^1(\mathcal {O})} + \widetilde{\mathbb {E}}\sup _{\tau \in [0,\,t]}|\overline{\widetilde{\mu }_{\lambda }}(\tau )|^\frac{p}{2} + \widetilde{\mathbb {E}}\left| \int _0^t \Vert \nabla \widetilde{\mu }_{\lambda }(\tau )\Vert _{{\boldsymbol{H}}}^2 \, {\mathrm d}\tau \right| ^\frac{p}{2} \nonumber \\  &\quad \le C\left[ 1+\widetilde{\mathbb {E}}\Vert \widetilde{\varphi }_{0}\Vert _H^{p} + \widetilde{\mathbb {E}}\Vert \Psi _\lambda (\widetilde{\varphi }_{0})\Vert _{L^1(\mathcal {O})}^\frac{p}{2} + \widetilde{\mathbb {E}}\left| \int _0^t |\overline{\widetilde{\mu }_{\lambda }(\tau )}|^2 \, {\mathrm d}\tau \right| ^\frac{p}{4} \right. \nonumber \\  &\qquad \left. + \widetilde{\mathbb {E}}\left| \int _0^t\sum _{k=0}^{+\infty }\int _\mathcal {O}|F_\lambda ^{\prime \prime }(\widetilde{\varphi }_{\lambda }(s))||G(\widetilde{\varphi }_{\lambda }(s))[u_k]|^2\,{\mathrm d}x \, {\mathrm d}s\right| ^{\frac{p}{2}} \right] , \end{aligned}$$

where C>0 is independent of λ. Finally, we observe that since the Yosida approximation is monotonically convergent to Ψ from below, we have

$$ \widetilde{\mathbb {E}}\Vert \Psi _\lambda (\widetilde{\varphi }_{0})\Vert _{L^1(\mathcal {O})}^\frac{p}{2} \le \widetilde{\mathbb {E}}\Vert \Psi (\widetilde{\varphi }_{0})\Vert _{L^1(\mathcal {O})}^\frac{p}{2} = \mathop { \mathbb {E}}\Vert \Psi (\varphi _{0})\Vert _{L^1(\mathcal {O})}^\frac{p}{2} $$

for any λ>0. The last term makes use of the fact that the approximation scheme satisfies the crucial property (thanks to Assumption (**A2**))

$$ \begin{aligned} |F''_\lambda (r)|&= |\Psi ''_\lambda (r)+\gamma | \\&= |\Psi ''(J_\lambda (r))J'_\lambda (r) + \gamma | \\&\le C\left( 1 + \Psi (J_\lambda (r)) \right) \\&\le C\left( 1 + \Psi _\lambda (r)) \right) \end{aligned} $$

for all $r \in \mathbb {R}$. The Hölder inequality then implies

$$ \begin{aligned}&\widetilde{\mathbb {E}}\left| \int _0^t\sum _{k=0}^{+\infty }\int _\mathcal {O}|F_\lambda ^{\prime \prime }(\widetilde{\varphi }_{\lambda }(s))||G(\widetilde{\varphi }_{\lambda }(s))[u_k]|^2\,{\mathrm d}x \, {\mathrm d}s\right| ^{\frac{p}{2}} \\&\hspace{1cm}\le C\left[ 1 + \widetilde{\mathbb {E}}\left| \int _0^t\sum _{k=0}^{+\infty }\int _\mathcal {O}|\Psi _\lambda (\widetilde{\varphi }_{\lambda }(s))||G(\widetilde{\varphi }_{\lambda }(s))[u_k]|^2\,{\mathrm d}x \, {\mathrm d}s\right| ^{\frac{p}{2}} \right] \\&\hspace{1cm}\le C\left[ 1 + \widetilde{\mathbb {E}}\left| \int _0^t\Vert \Psi _\lambda (\widetilde{\varphi }_{\lambda }(s))\Vert _{L^1(\mathcal {O})}\sum _{k=0}^{+\infty } \Vert G(\widetilde{\varphi }_{\lambda }(s))[u_k]\Vert _{L^\infty (\mathcal {O})}^2 \, {\mathrm d}s\right| ^{\frac{p}{2}} \right] \\&\hspace{1cm}\le C\left[ 1 + \widetilde{\mathbb {E}}\left| \int _0^t\Vert \Psi _\lambda (\widetilde{\varphi }_{\lambda }(s))\Vert _{L^1(\mathcal {O})} \, {\mathrm d}s\right| ^{\frac{p}{2}} \right] . \end{aligned} $$

Injecting in (3.33) gives

$$\begin{aligned}&\widetilde{\mathbb {E}}\sup _{\tau \in [0,\,t]}\mathcal \Vert \Psi _\lambda (\widetilde{\varphi }_{\lambda }(\tau ))\Vert ^\frac{p}{2}_{L^1(\mathcal {O})} + \widetilde{\mathbb {E}}\sup _{\tau \in [0,\,t]}|\overline{\widetilde{\mu }_{\lambda }}(\tau )|^\frac{p}{2} + \widetilde{\mathbb {E}}\left| \int _0^t \Vert \nabla \widetilde{\mu }_{\lambda }(\tau )\Vert _{{\boldsymbol{H}}}^2 \, {\mathrm d}\tau \right| ^\frac{p}{2} \\  &\quad \le C\left[ 1+\widetilde{\mathbb {E}}\Vert \widetilde{\varphi }_{0}\Vert _H^{p} + \widetilde{\mathbb {E}}\Vert \Psi _\lambda (\widetilde{\varphi }_{0})\Vert _{L^1(\mathcal {O})}^\frac{p}{2} + \widetilde{\mathbb {E}}\left| \int _0^t |\overline{\widetilde{\mu }_{\lambda }(\tau )}|^2 \, {\mathrm d}\tau \right| ^\frac{p}{4} \right. \\&\left. \qquad + \widetilde{\mathbb {E}}\left| \int _0^t\Vert \Psi _\lambda (\widetilde{\varphi }_{\lambda }(s))\Vert _{L^1(\mathcal {O})} \, {\mathrm d}s\right| ^{\frac{p}{2}} \right] , \end{aligned}$$

and the Gronwall lemma gives the second estimate

$$\begin{aligned} \Vert \widetilde{\mu }_{\lambda }\Vert _{L^\frac{p}{2}(\widetilde{\Omega };L^2(0,T;V))} + \Vert \nabla \widetilde{\mu }_{\lambda }\Vert _{L^p(\widetilde{\Omega };L^2(0,T;{\boldsymbol{H}}))}+\Vert \Psi _\lambda (\widetilde{\varphi }_\lambda ))\Vert _{L^\frac{p}{2}(\widetilde{\Omega };L^\infty (0,T;L^1(\mathcal {O})))} \le C_2, \end{aligned}$$

and the proof is complete. □

The third and fourth estimates play out very similarly with respect to what is carried out in the proofs of Lemmas 3.7 and 3.8. Indeed, they are essentially corollaries of the first two estimates. For the sake of brevity, we do not report the full computations, but only highlight the results in the following lemmas.

#### Lemma 3.15

It holds that

(i) the family $\{G(\widetilde{\varphi }_{\lambda })\}_{\lambda > 0}$ is uniformly bounded in
  $$ L^\infty (\Omega \times (0,T);\mathscr {L}^2(U,H))\cap L^p(\Omega ;L^2(0,T;\mathscr {L}^2(U,V))) $$
  for all p≥2;
(ii) the family $\left\{ \displaystyle \int _0^. G(\widetilde{\varphi }_{\lambda }(s)) \, {\mathrm d}W(s)\right\} _{\lambda > 0}$ is uniformly bounded in
  $$ L^q(\Omega ;W^{k,q}(0,T;H))\cap L^2(\Omega ;H^{k}(0,T;V)) $$
  for all $k \in (0,\frac{1}{2})$ and q≥2.

Exploiting the weak formulation of the problem (passing to the limit of $n\rightarrow +\infty$ therein, owing to the proven convergences) we eventually also show that

#### Lemma 3.16

The family $\{\widetilde{\varphi }_{\lambda }\}_{\lambda > 0}$ is uniformly bounded in $L^p(\widetilde{\Omega };W^{\alpha ,p}(0,T;V^*))$ for all $\alpha \in (0, \frac{1}{2})$ and p≥2.

Finally, let us provide an additional estimate, since in order to pass to the limit as $\lambda \rightarrow 0^+$ we can no longer rely on the Lipschitz continuity of $\Psi '_\lambda$, as the Lipschitz constants are not uniformly bounded in λ.

#### Lemma 3.17

The family $\{F^\prime _\lambda (\widetilde{\varphi }_\lambda )\}_{\lambda > 0}$ is uniformly bounded in $L^{\frac{p}{2}}(\widetilde{\Omega };L^2(0,T;H))$ for all p≥2.

#### Proof

Testing the equation for $\widetilde{\mu }_\lambda$ by $\Psi _\lambda (\widetilde{\varphi }_\lambda ) = F_\lambda ^\prime (\widetilde{\varphi }_\lambda ) + \gamma \widetilde{\varphi }_\lambda$, we have

$$\begin{aligned}&\int _\mathcal {O}\widetilde{\mu }_\lambda F_\lambda ^\prime (\widetilde{\varphi }_\lambda ) \, {\mathrm d}x + \gamma \int _\mathcal {O}\widetilde{\mu }_\lambda \widetilde{\varphi }_\lambda \, {\mathrm d}x \\  &\quad =\int _\mathcal {O}a {\widetilde{\varphi }_\lambda }F_\lambda '(\widetilde{\varphi }_\lambda )\, {\mathrm d}x + {\gamma }\int _\mathcal {O}a{\widetilde{\varphi }_\lambda ^2}\, {\mathrm d}x - \int _\mathcal {O}(K*\widetilde{\varphi }_\lambda )F^\prime _\lambda (\widetilde{\varphi }_\lambda ) \,{\mathrm d}x \\&\qquad - \gamma \int _\mathcal {O}(K*\widetilde{\varphi }_\lambda )\widetilde{\varphi }_\lambda \,{\mathrm d}x + \int _\mathcal {O}|F^\prime _\lambda (\widetilde{\varphi }_\lambda )|^2{\mathrm d}x + \gamma \int _\mathcal {O}\widetilde{\varphi }_\lambda F'_\lambda (\widetilde{\varphi }_\lambda ) \, {\mathrm d}x. \end{aligned}$$

Rearranging terms, we can use Young’s inequality multiple times to conclude

$$\begin{aligned} \Vert F_\lambda ^\prime (\widetilde{\varphi }_\lambda )\Vert _{L^2(0,T;H)}^2 \le C\left[ 1 + \Vert \widetilde{\mu }_\lambda \Vert _{L^2(0,T;H)}^2 + \Vert \widetilde{\varphi }_\lambda \Vert _{L^2(0,T;H)}^2\right] , \end{aligned}$$

for a constant C>0 independent of λ. Therefore, thanks to Lemmas 3.13 and 3.14, we infer that

$$\begin{aligned} \Vert F^\prime _\lambda (\widetilde{\varphi }_\lambda )\Vert _{L^{\frac{p}{2}}(\widetilde{\Omega };L^2(0,T;H))}\le C_5 \end{aligned}$$

for some $C_5>0$ independent of λ>0. □

### Passage to the limit as $\lambda \rightarrow 0^+$

The previous estimates allow us to pass to the limit as $\lambda \rightarrow 0^+$, by means of a second stochastic compactness argument. From this subsection onwards, we assume to fix once and for all a suitable vanishing sequence $\{\lambda _n\}_{n \in \mathbb {N}}$ and we understand without relabeling that the passage to the limit is performed along said sequence. The sequence of laws of $\{\varphi _{\lambda }\}_{\lambda > 0}$ is tight in the same path space illustrated in Subsection 3.3. To this end, we still make use of the compact embeddings (see [20, Theorem 2.1])

$$\begin{aligned} L^2(0,T;V)\cap H^\alpha (0,T;V^*)&\hookrightarrow L^2(0,T;H) \\ L^\infty (0,T;H) \cap W^{\alpha , p}(0,T;V^*)&\hookrightarrow C^0([0,T]; H^{-\delta }(\mathcal {O})) \end{aligned}$$

still assuming that αp>1, i.e., that p>2, and for some small δ>0. The following tightness lemmas hold; their proofs closely follow the corresponding ones in Lemmas 3.9-3.12.

#### Lemma 3.18

If p>2, then the family of laws of $\{\widetilde{\varphi }_{\lambda }\}_{\lambda > 0}$ is tight in the space $\mathcal {X}:= L^2(0,T;H) \cap C^0([0,T]; H^{-\delta }(\mathcal {O}))$ for any $\delta \in (0, \frac{1}{2})$.

#### Lemma 3.19

The family of laws of $\{G(\widetilde{\varphi }_\lambda ) \cdot \widetilde{W}\}_{\lambda > 0}$ is tight in the space $C^0([0,T]; V^*)$.

By identifying $\widetilde{W}$ with a constant sequence of random variables $\{\widetilde{W}_\lambda \}_{\lambda > 0}$ with values in $C^0([0,T];U_0)$, we also get that

#### Lemma 3.20

The family of laws of $\{\widetilde{W}_\lambda \}_{\lambda > 0}$ is tight in the space $C^0([0,T]; U_0)$.

By the same token, we identify $\widetilde{\varphi }_0$ with a constant sequence $\{\widetilde{\varphi }_{0,\lambda }\}_{\lambda >0}$ that is again tight in $V^*$ as $H \hookrightarrow V^*$ compactly. Namely, we have

#### Lemma 3.21

The family of laws of $\{\widetilde{\varphi }_{0,\lambda }\}_{\lambda > 0}$ is tight in $V^*$.

Collecting Lemmas 3.18-3.21, we obtain that the family of laws

$$\begin{aligned} \{\widetilde{\varphi }_{\lambda },\, G(\widetilde{\varphi }_{\lambda })\cdot W,\, W_\lambda ,\, \varphi _{0,\lambda }\}_{\lambda >0} \end{aligned}$$

is tight in the space

$$\begin{aligned} \mathcal {X} \times C^0([0,T]; V^*) \times C^0([0,T];U_0) \times V^*. \end{aligned}$$

Owing to the Prokhorov and Skorokhod theorems (see [32, Theorem 2.7] and [39, Theorem 1.10.4, Addendum 1.10.5]), there exists a probability space $(\widehat{\Omega }, \widehat{\mathscr {F}},\widehat{\mathbb {P}})$ and a family of random variables $Y_\lambda : (\widehat{\Omega }, \widehat{\mathscr {F}})\rightarrow (\widetilde{\Omega }, \widetilde{\mathscr {F}})$ such that the law of $Y_\lambda$ is $\widetilde{\mathbb {P}}$ for every $\lambda \in (0,1)$, namely $\widehat{\mathbb {P}}\circ Y_\lambda ^{-1} = \widetilde{\mathbb {P}}$ (so that composition with $Y_\lambda$ preserves laws), and, thanks to the Vitali and Banach–Alaoglu theorems and reflexivity, the following convergences hold as $\lambda \rightarrow 0^+$:

$$\begin{aligned} \widehat{\varphi }_{\lambda }:= \widetilde{\varphi }_\lambda \circ Y_\lambda \rightarrow \widehat{\varphi }&\quad \text { in }L^q(\widehat{\Omega };L^2(0,T;H) \cap C^0([0,T]; H^{-\delta }(\mathcal {O}))\;\;\forall \, q<p, \\ \widehat{\varphi }_{\lambda } \overset{*}{\rightharpoonup }\ \widehat{\varphi }&\quad \text { in }L^p(\widehat{\Omega };H^\alpha (0,T;V^*)),\\ \widehat{\varphi }_{\lambda } \rightharpoonup \widehat{\varphi }&\quad \text { in }L^p(\widehat{\Omega };L^2(0,T;V)), \\ \widehat{\mu }_{\lambda } := \widetilde{\mu }_{\lambda }\circ Y_\lambda \rightharpoonup \widehat{\mu }&\quad \text { in }L^{\frac{p}{2}}(\widehat{\Omega };L^2(0,T;V)), \\ \nabla \widehat{\mu }_{\lambda } \rightharpoonup \nabla \widehat{\mu }&\quad \text { in }L^p(\widehat{\Omega };L^2(0,T;{\boldsymbol{H}})), \\ \widehat{I}_\lambda := (G(\widetilde{\varphi }_\lambda )\cdot \widetilde{W}) \circ Y_\lambda \rightarrow \widehat{I}&\quad \text { in }L^q(\widehat{\Omega };C^0([0,T];H))\;\;\forall \, q<p,\\ \widehat{W}_\lambda := \widetilde{W}\circ Y_\lambda \rightarrow \widehat{W}&\quad \text { in }L^q(\widehat{\Omega };C^0([0,T];U))\;\;\forall \, q<p, \end{aligned}$$

for some limiting measurable processes enjoying the following regularity properties:

$$\begin{aligned} \widehat{\varphi }&\in L^p_w(\widehat{\Omega };L^\infty (0,T;H))\cap L^p(\widehat{\Omega };H^\alpha (0,T;V^*))\\&\cap L^p(\widehat{\Omega };L^2(0,T;V) \cap C^0([0,T]; H^{-\delta }(\mathcal {O})),\\ \widehat{\mu }&\in L^\frac{p}{2}(\widehat{\Omega };L^2(0,T;V)),\\ \nabla \widehat{\mu }&\in L^{p}(\widehat{\Omega };L^2(0,T;{\boldsymbol{H}})),\\ \widehat{I}&\in L^p(\widehat{\Omega };C^0([0,T];V^*)), \\ \widehat{W}&\in L^p(\widehat{\Omega };C^0([0,T];U)), \\ \widehat{\varphi }_0&\in L^p(\widehat{\Omega }; H). \end{aligned}$$

Finally, we address once again the nonlinearities of the problem. Since the space $L^\frac{p}{2}(\widehat{\Omega };L^2(0,T;H))$ is reflexive for p>2, the uniform bound given in Lemma 3.17 yields

$$ F'_\lambda (\widehat{\varphi }_\lambda ) \rightharpoonup \xi \quad \text {in } L^\frac{p}{2}(\widehat{\Omega };L^2(0,T;H)). $$

Let us show that indeed $\xi = \Psi '(\widehat{\varphi }) -\gamma \widehat{\varphi }= {F'}(\widehat{\varphi })$. Using the strong convergence of

$$ \widehat{\varphi }_{\lambda } \rightarrow \widehat{\varphi }\quad \text { in }L^q(\widehat{\Omega };L^2(0,T;H)) $$

for q<p, by comparison it is immediate to deduce that

$$ \Psi '_\lambda (\widehat{\varphi }_\lambda ) \rightharpoonup \xi + \gamma \widehat{\varphi }\quad \text {in } L^\frac{p}{2}(\widehat{\Omega };L^2(0,T;H)). $$

As by definition it holds $\Psi '_\lambda = \Psi ' \circ J_\lambda$, we can investigate the sequence $\{J_\lambda (\widehat{\varphi }_\lambda )\}_{\lambda > 0}$, namely

$$ \begin{aligned} \Vert J_\lambda (\widehat{\varphi }_\lambda (s)) - \widehat{\varphi }(s)\Vert ^2_H&\le 2\Vert J_\lambda (\widehat{\varphi }_\lambda (s))-\widehat{\varphi }_\lambda (s)\Vert ^2_H + 2\Vert \widehat{\varphi }_\lambda (s)-\widehat{\varphi }(s)\Vert ^2_H \\&= 2\lambda ^2\Vert \Psi '_\lambda (\widehat{\varphi }_\lambda (s))\Vert ^2_H + 2\Vert \widehat{\varphi }_\lambda (s)-\widehat{\varphi }(s)\Vert ^2_H \end{aligned} $$

implying that

$$ J_\lambda (\widehat{\varphi }_\lambda ) \rightarrow \widehat{\varphi }\quad \text {in } L^\frac{p}{2}(\widehat{\Omega };L^2(0,T;H)), $$

thanks to Lemma 3.14. Then, the strong-weak closure of maximal monotone operators (see [2, Proposition 2.1] implies $\xi + \gamma \widehat{\varphi }= \Psi '(\widehat{\varphi })$, as claimed. Given (**A3**), we immediately have

$$\begin{aligned} \Vert G(\widehat{\varphi }_{\lambda }) - G(\widehat{\varphi })\Vert _{L^q(\widehat{\Omega };L^2(0,T;\mathscr {L}^2(U,H)))} \rightarrow 0 \end{aligned}$$

as $\lambda \rightarrow 0^+$, it follows that

$$\begin{aligned} G(\widehat{\varphi }_{\lambda }) \rightarrow G(\widehat{\varphi })\quad \text { in }L^q(\widehat{\Omega };L^2(0,T;\mathscr {L}^2(U,H))) \end{aligned}$$

for all q<p.

### Identification of the limit solution

This is essentially a *verbatim* iteration of the argument presented in Subsection 3.4, and is therefore omitted. However, it completes the proof of existence by retrieving a martingale solution to (1.6).

## Pathwise uniqueness and existence of probabilistically-strong solutions

The present section is devoted to proving Theorem 2.7 and Corollary 2.8, i.e., to showing a continuous dependence estimate for problem (1.6), entailing pathwise uniqueness in dimension two and three and, in turn, existence and uniqueness of a probabilistically-strong solution for the stochastic nonlocal Cahn–Hilliard equation. For $i \in \{1,2\}$, let $\varphi _{0i}$ comply with the assumptions listed in Definition 2.2. By Theorem 2.4, the formal problem

4.1 $$\begin{aligned} {\left\{ \begin{array}{ll} {\mathrm d}\widehat{\varphi }_i - \Delta \widehat{\mu }_i = G(\widehat{\varphi }_i) \, {\mathrm d}W &  \quad \text {in }(0,T) \times \mathcal {O}, \\ \widehat{\mu }_i = a\widehat{\varphi }_i - K * \widehat{\varphi }_i + F'(\widehat{\varphi }_i) &  \quad \text {in }(0,T) \times \mathcal {O}, \\ \partial _{{\boldsymbol{n}}} \widehat{\mu }_i = 0 &  \quad \text {on }(0,T) \times \partial \mathcal {O}, \\ \widehat{\varphi }(0) = \varphi _{0i} &  \quad \text {in } \mathcal {O}, \end{array}\right. } \end{aligned}$$

admits at least one martingale solution $\widehat{\varphi }_i$ for $i \in \{1,2\}$, defined on a suitable filtered probability space and satisfying (4.1) with respect to some cylindrical Wiener process on *U*. Assuming that $\widehat{\varphi }_1$ and $\widehat{\varphi }_2$ are defined on the same stochastic basis

$$ \left\{ \widehat{\Omega },\, \widehat{\mathscr {F}},\, (\widehat{\mathscr {F}}_t)_{t \in [0,T]},\, \widehat{\mathbb {P}}\right\} $$

and satisfy (4.1) with respect to the same cylindrical Wiener process $\widehat{W}$ on *U*, we define the differences

$$ \widehat{\varphi }:= \widehat{\varphi }_1 -\widehat{\varphi }_2, \qquad \widehat{\mu }:= \widehat{\mu }_1-\widehat{\mu }_2, \qquad \widehat{\varphi }_0 := \widehat{\varphi }_{1}(0) - \widehat{\varphi }_{2}(0). $$

Under the additional assumption (**A4**), it is straightforward to see that the process is pathwise mass-conservative, as one can easily see by plugging a constant nonzero function in the variational formulation of the problem (see Definition 2.2). In particular, it is immediate to observe that

$$ \overline{\varphi _{01}} =\overline{\varphi _{02}} \Rightarrow \overline{\widehat{\varphi }(t)} = 0 $$

for all t≥0, $\mathbb {P}$-almost surely. Applying the Itô lemma for twice Fréchet-differentiable functionals (see [8, Theorem 4.32]) to

$$ \Gamma : V^* \rightarrow \mathbb {R}, \qquad \Gamma (u) = \dfrac{1}{2} \Vert \nabla \mathcal {N} (u -|\mathcal {O}|^{-1}\langle u, 1\rangle _{V^*,V})\Vert ^2_{\boldsymbol{H}}, $$

again, evaluated at $u = \widehat{\varphi }(t)$. This results in

4.2 $$\begin{aligned}&\dfrac{1}{2} \Vert \nabla \mathcal {N} \widehat{\varphi }(t)\Vert ^2_{\boldsymbol{H}} + \int _0^t (\widehat{\varphi }(\tau ),\,\widehat{\mu }(\tau ))_H\, {\mathrm d}\tau \nonumber \\&\quad = \dfrac{1}{2} \Vert \nabla \mathcal {N} \widehat{\varphi }_0\Vert ^2_{\boldsymbol{H}} + \dfrac{1}{2} \int _0^t \left\| \nabla \mathcal {N} \left[ G(\widehat{\varphi }_1(\tau ))-G(\widehat{\varphi }_2(\tau )) - \left( \overline{G(\widehat{\varphi }_1(\tau ))} - \overline{G(\widehat{\varphi }_2(\tau ))} \right) \right] \right\| ^2_{\mathscr {L}^2(U, {\boldsymbol{H}})} \, {\mathrm d}\tau \nonumber \\  &\qquad + \int _0^t \left( \widehat{\varphi }(\tau ), \, \mathcal {N} \left[ G(\widehat{\varphi }_1(\tau ))-G(\widehat{\varphi }_2(\tau )) - \left( \overline{G(\widehat{\varphi }_1(\tau ))} - \overline{G(\widehat{\varphi }_2(\tau ))} \right) \right] \, {\mathrm d}\widehat{W}\right) _H. \end{aligned}$$

Using Assumption (**A2**) and the definition of $\widehat{\mu }$, we get

4.3 $$\begin{aligned} \begin{aligned}&\int _0^t (\widehat{\varphi }(\tau ),\,\widehat{\mu }(\tau ))_H\, {\mathrm d}\tau \\&\quad = \int _0^t (\widehat{\varphi }(\tau ),\,a\widehat{\varphi }(\tau ))_H \, {\mathrm d}\tau - \int _0^t (\widehat{\varphi }(\tau ),\,K * \widehat{\varphi }(\tau ))_H\, {\mathrm d}\tau \\&\qquad + \int _0^t (\widehat{\varphi }(\tau ),\,F'(\widehat{\varphi }_1(\tau ))-F'(\widehat{\varphi }_2(\tau )))_H\, {\mathrm d}\tau \\&\quad \ge C_0\int _0^t\Vert \widehat{\varphi }(\tau )\Vert ^2_H \, {\mathrm d}\tau + \int _0^t (\nabla \mathcal {N}\widehat{\varphi }(\tau ),\,\nabla K * \widehat{\varphi }(\tau ))_{\boldsymbol{H}}\, {\mathrm d}\tau . \end{aligned} \end{aligned}$$

Moreover, we have

4.4 $$\begin{aligned} \left| \int _0^t (\nabla \mathcal {N}\widehat{\varphi }(\tau ),\,\nabla K * \widehat{\varphi }(\tau ))_{\boldsymbol{H}}\, {\mathrm d}\tau \right| \le \dfrac{C_0}{2}\int _0^t \Vert \widehat{\varphi }(\tau )\Vert _H^2 \, {\mathrm d}\tau + \dfrac{\Vert \nabla K\Vert _{{\boldsymbol{L}}^1(B_R)}^2}{2C_0} \int _0^t \Vert \nabla \mathcal {N}\widehat{\varphi }(\tau )\Vert _{{\boldsymbol{H}}}^2 \, {\mathrm d}\tau . \end{aligned}$$

Recalling [15, Proposition 2.1], we observe that

$$ \begin{aligned} \Vert G(\widehat{\varphi }_1(\tau )) - G(\widehat{\varphi }_2(\tau ))\Vert ^2_{\mathscr {L}^2(U,V^*)}&= \sum _{k=0}^{+\infty } \Vert G(\widehat{\varphi }_1(\tau ))[u_k] - G(\widehat{\varphi }_2(\tau ))[u_k]\Vert ^2_{V^*} \\&\ge C\sum _{k=0}^{+\infty } \left\| \nabla \mathcal {N} \left[ G(\widehat{\varphi }_1(\tau ))[u_k] - G(\widehat{\varphi }_2(\tau ))[u_k] \right] \right\| ^2_{{\boldsymbol{H}}} \end{aligned} $$

for a constant C>0 only depending on $\mathcal {O}$, and therefore, by possibly updating the value of *C*,

4.5 $$\begin{aligned} \begin{aligned} \int _0^t \left\| \nabla \mathcal {N} \left[ G(\widehat{\varphi }_1(\tau ))-G(\widehat{\varphi }_2(\tau )) \right] \right\| ^2_{\mathscr {L}^2(U, {\boldsymbol{H}})} \, {\mathrm d}\tau&\le C\int _0^t \Vert G(\widehat{\varphi }_1(\tau )) - G(\widehat{\varphi }_2(\tau ))\Vert ^2_{V^*} \, {\mathrm d}\tau \\&\le CL_G^2\int _0^t \Vert \widehat{\varphi }(\tau )\Vert ^2_{V^*} \, {\mathrm d}\tau \end{aligned} \end{aligned}$$

for some constant C>0 only depending on $|\mathcal {O}|$. Using (4.3)-(4.5) in (4.2) yields

4.6 $$\begin{aligned}  &   \dfrac{1}{2} \Vert \nabla \mathcal {N} \widehat{\varphi }(t)\Vert ^2_{\boldsymbol{H}} + \dfrac{C_0}{2} \int _0^t \Vert \widehat{\varphi }(\tau )\Vert _H^2\, {\mathrm d}\tau \le \dfrac{1}{2} \Vert \nabla \mathcal {N} \widehat{\varphi }_0\Vert ^2_{\boldsymbol{H}} +C\widehat{L}_G^2\int _0^t \Vert \widehat{\varphi }(\tau )\Vert ^2_{V^*} \, {\mathrm d}\tau \nonumber \\    &   + \dfrac{\Vert \nabla K\Vert _{{\boldsymbol{L}}^1(B_R)}^2}{2C_0} \int _0^t \Vert \nabla \mathcal {N}\widehat{\varphi }(\tau )\Vert _{{\boldsymbol{H}}}^2 \, {\mathrm d}\tau + \int _0^t \left( \widehat{\varphi }(\tau ), \, \mathcal {N} \left[ G(\widehat{\varphi }_1(\tau ))-G(\widehat{\varphi }_2(\tau ))\right] \, {\mathrm d}\widehat{W}\right) _H. \end{aligned}$$

Raising (4.6) to the power $\frac{p}{2}$, taking supremums in time and $\widehat{\mathbb {P}}$-expectations leads to

$$\begin{aligned}  &   \widehat{\mathbb {E}}\sup _{\tau \in [0,\,t]}\Vert \widehat{\varphi }(\tau )\Vert ^p_{V^*} + \widehat{\mathbb {E}}\left| \int _0^t \Vert \widehat{\varphi }(\tau )\Vert ^2_H \, {\mathrm d}\tau \right| ^\frac{p}{2} \le C\left[ \Vert \widehat{\varphi }_0\Vert ^p_{V^*} + \widehat{\mathbb {E}}\left| \int _0^t \Vert \widehat{\varphi }(\tau )\Vert ^2_{V^*} \, {\mathrm d}\tau \right| ^\frac{p}{2} \right. \\  &   \quad \quad \left. + \widehat{\mathbb {E}}\sup _{\tau \in [0,\,t]}\left| \int _0^\tau \left( \widehat{\varphi }(s), \, \mathcal {N} \left[ G(\widehat{\varphi }_1(s))-G(\widehat{\varphi }_2(s)) \right] \, {\mathrm d}\widehat{W}(s) \right) _H \right| ^\frac{p}{2} \right] , \end{aligned}$$

where the constant C>0 now also depends on *p*. We are then left with estimating the stochastic integrals on the right hand side of the inequality above. This is done by exploiting the Burkholder–Davis–Gundy inequality. Indeed, recalling (4.5) (see also [14, Section 4])

$$\begin{aligned} \begin{aligned}&\widehat{\mathbb {E}}\sup _{\tau \in [0,\,t]}\left| \int _0^\tau \left( \widehat{\varphi }(s), \, \mathcal {N} \left[ G(\widehat{\varphi }_1(s))-G(\widehat{\varphi }_2(s)) \right] \, {\mathrm d}\widehat{W}(s) \right) _H \right| ^\frac{p}{2} \\&\hspace{0.3cm} \le C\widehat{\mathbb {E}}\left| \int _0^t \Vert \nabla \mathcal {N}\widehat{\varphi }(\tau )\Vert _{\boldsymbol{H}}^2\left\| \nabla \mathcal {N} \left[ G(\widehat{\varphi }_1(\tau ))-G(\widehat{\varphi }_2(\tau )) \right] \right\| ^2_{\mathscr {L}^2(U, {\boldsymbol{H}})} \, {\mathrm d}\tau \right| ^\frac{p}{4} \\&\hspace{0.3cm} \le C\widehat{\mathbb {E}}\left[ \sup _{\tau \in [0,\,t]}\Vert \nabla \mathcal {N} \widehat{\varphi }(\tau )\Vert _{\boldsymbol{H}}^\frac{p}{2} \left| \int _0^t \Vert \widehat{\varphi }(\tau )\Vert ^2_{V^*} \, {\mathrm d}\tau \right| ^\frac{p}{4} \right] \\&\hspace{0.3cm} \le \dfrac{1}{4}\widehat{\mathbb {E}}\sup _{\tau \in [0,\,t]}\Vert \widehat{\varphi }(\tau )\Vert _{V^*}^p + C\widehat{\mathbb {E}}\left| \int _0^t \Vert \widehat{\varphi }(\tau )\Vert ^2_{V^*} \, {\mathrm d}\tau \right| ^\frac{p}{2}. \end{aligned} \end{aligned}$$

In light of all of the above, we conclude that

4.7 $$\begin{aligned} \widehat{\mathbb {E}}\sup _{\tau \in [0,\,t]}\Vert \widehat{\varphi }(\tau )\Vert ^p_{V^*} + \widehat{\mathbb {E}}\left| \int _0^t \Vert \widehat{\varphi }(\tau )\Vert ^2_H \, {\mathrm d}\tau \right| ^\frac{p}{2} \le C\left[ \Vert \widehat{\varphi }_0\Vert ^p_{V^*} + \widehat{\mathbb {E}}\left| \int _0^t \Vert \widehat{\varphi }(\tau )\Vert ^2_{V^*} \, {\mathrm d}\tau \right| ^\frac{p}{2} \right] , \end{aligned}$$

for a constant C>0 only depending on the structural parameters of the problem, including *p* and *T*. Therefore, (4.7) and the Gronwall lemma imply that

$$\begin{aligned} \Vert \widehat{\varphi }\Vert ^p_{L^p(\widehat{\Omega }; L^\infty (0,T;V^*))} + \Vert \widehat{\varphi }\Vert ^p_{L^p(\widehat{\Omega }; L^2(0,T;H))} \le C\Vert \widehat{\varphi }_0\Vert ^p_{L^p(\widehat{\Omega };V^*)}, \end{aligned}$$

and choosing $\varphi _{01} = \varphi _{02}$ in (4.1) leads to pathwise uniqueness, and the proof is complete.

## Nonlocal-to-local asymptotics: potentials of polynomial growth

Hereafter, we show the proof of Theorem 2.12, i.e., that probabilistically-strong solutions to the stochastic nonlocal Cahn–Hilliard problem (2.4) converge to the probabilistically-strong solution to the local problem (2.2) as $\varepsilon \rightarrow 0^+$. In particular, for any ε>0, the process $\varphi _\varepsilon$ solves the nonlocal problem (2.4), that for convenience we recall here:

5.1 $$\begin{aligned} {\left\{ \begin{array}{ll} {\mathrm d}\varphi _\varepsilon - \Delta \mu _\varepsilon \, {\mathrm d}t = G(\varphi _\varepsilon ) \, {\mathrm d}W &  \quad \text {in } (0,T) \times \mathcal {O}, \\ \mu _\varepsilon = \mathcal {L}_\varepsilon \varphi _\varepsilon + F'(\varphi _\varepsilon )&  \quad \text {in } (0,T) \times \mathcal {O}, \\ \partial _{{\boldsymbol{n}}} \mu _\varepsilon = 0 &  \quad \text {on } (0,T) \times \partial \mathcal {O}, \\ \varphi _\varepsilon (0) = \varphi _{0,\varepsilon } &  \quad \text {in } \mathcal {O}. \end{array}\right. } \end{aligned}$$

On the other hand, the process φ solves the local Cahn–Hilliard system (2.2), reading

5.2 $$\begin{aligned} {\left\{ \begin{array}{ll} {\mathrm d}\varphi - \Delta \mu \, {\mathrm d}t = G(\varphi ) \, {\mathrm d}W &  \quad \text {in } (0,T) \times \mathcal {O}, \\ \mu = -\Delta \varphi + F'(\varphi )&  \quad \text {in } (0,T) \times \mathcal {O}, \\ \partial _{{\boldsymbol{n}}} \mu = \partial _{\boldsymbol{n}}\varphi =0 &  \quad \text {on } (0,T) \times \partial \mathcal {O}, \\ \varphi (0) = \varphi _0 &  \quad \text {in } \mathcal {O}. \end{array}\right. } \end{aligned}$$

Since both problems admit a unique probabilistically-strong solution, we fix once and for all a filtered probability space

$$ \left\{ \Omega ,\, \mathscr {F},\, (\mathscr {F}_t)_{t \in [0,T]},\, \mathbb {P}\right\} $$

and, accordingly, a cylindrical Wiener process *W* on *U* to formulate (5.1) and (5.2). As in the previous section, we define the differences

$$ \xi := \varphi _\varepsilon - \varphi , \qquad \eta := \mu _\varepsilon -\mu , \qquad \xi _0 := \varphi _{0,\varepsilon }-\varphi _{0}. $$

Once again, Assumption (**A4**) yields the key conservation property

$$ \overline{\xi (t)} = 0 $$

for all t≥0, $\mathbb {P}$-almost surely and for all ε>0. Applying the Itô lemma for twice Fréchet-differentiable functionals (see [8, Theorem 4.32]) to

$$ \Gamma : V^* \rightarrow \mathbb {R}, \qquad \Gamma (u) = \dfrac{1}{2} \Vert \nabla \mathcal {N} (u -|\mathcal {O}|^{-1}\langle u, 1\rangle _{V^*,V})\Vert ^2_{\boldsymbol{H}}, $$

evaluated at u=ξ(t) results in

5.3 $$\begin{aligned}&\dfrac{1}{2} \Vert \nabla \mathcal {N} \xi (t)\Vert ^2_{\boldsymbol{H}} + \int _0^t (\xi (\tau ),\,\eta (\tau ))_H\, {\mathrm d}\tau \nonumber \\&\quad = \dfrac{1}{2} \Vert \nabla \mathcal {N} \xi _0\Vert ^2_{\boldsymbol{H}} + \dfrac{1}{2} \int _0^t \left\| \nabla \mathcal {N} \left[ G(\varphi _\varepsilon (\tau ))-G(\varphi (\tau )) \right] \right\| ^2_{\mathscr {L}^2(U, {\boldsymbol{H}})} \, {\mathrm d}\tau \nonumber \\&\qquad + \int _0^t \left( \xi (\tau ), \, \mathcal {N} \left[ G(\varphi _\varepsilon (\tau ))-G(\varphi (\tau )) \right] \, {\mathrm d}W \right) _H. \end{aligned}$$

Expanding the scalar product to the left hand side, by summing and subtracting $\mathcal {L}_\varepsilon \varphi$ we get

5.4 $$\begin{aligned} \int _0^t (\xi (\tau ),\,\eta (\tau ))_H\, {\mathrm d}\tau&= \int _0^t (\xi (\tau ),\,\mathcal {L}_\varepsilon \varphi (\tau )+\Delta \varphi (\tau ))_H \, {\mathrm d}\tau + \int _0^t (\xi (\tau ),\,\mathcal {L}_\varepsilon \xi (\tau )) \, {\mathrm d}\tau \nonumber \\&\quad +\int _0^t (\xi (\tau ),\,F'(\varphi _\varepsilon (\tau ))-F'(\varphi (\tau )))_H\, {\mathrm d}\tau . \end{aligned}$$

On the right hand side of (5.4), we control the three terms as follows. We exploit [1, Corollary 4.2] and the Young inequality to infer that

5.5 $$\begin{aligned} \begin{aligned} \left| \int _0^t (\xi (\tau ),\,\mathcal {L}_\varepsilon \varphi (\tau )+\Delta \varphi (\tau ))_H \, {\mathrm d}\tau \right| \le \frac{1}{2} \int _0^t \Vert \xi (\tau )\Vert ^2_H \, {\mathrm d}\tau +C\varepsilon \int _0^t\Vert \varphi (\tau )\Vert ^2_{H^3(\mathcal {O})} \, {\mathrm d}\tau , \end{aligned} \end{aligned}$$

while, given the positivity of the kernel $K_\varepsilon$, we can apply the nonlocal interpolation inequality [9, Lemma 3.4]

5.6 $$\begin{aligned} \int _0^t (\xi (\tau ),\,\mathcal {L}_\varepsilon \xi (\tau )) \, {\mathrm d}\tau \ge (C_F +1)\int _0^t \Vert \xi (\tau )\Vert ^2_H \, {\mathrm d}\tau - C\int _0^t \Vert \xi (\tau )\Vert ^2_{V^*} \,{\mathrm d}\tau , \end{aligned}$$

and finally by Assumption (**A2**)

5.7 $$\begin{aligned} \int _0^t (\xi (\tau ),\,F'(\varphi _\varepsilon (\tau ))-F'(\varphi (\tau )))_H\, {\mathrm d}\tau \ge - C_F\int _0^t \Vert \xi (\tau )\Vert ^2_H \, {\mathrm d}\tau . \end{aligned}$$

The stochastic diffusion can be handled as usual, i.e., recalling again [15, Proposition 2.1], we have

5.8 $$\begin{aligned} \begin{aligned} \int _0^t \left\| \nabla \mathcal {N} \left[ G(\varphi _\varepsilon (\tau ))-G(\varphi (\tau )) \right] \right\| ^2_{\mathscr {L}^2(U, {\boldsymbol{H}})} \, {\mathrm d}\tau&\le C\int _0^t \Vert G(\varphi _\varepsilon (\tau )) - G(\varphi (\tau ))\Vert ^2_{V^*} \, {\mathrm d}\tau \\&\le CL_G^2\int _0^t \Vert \xi (\tau )\Vert ^2_{V^*} \, {\mathrm d}\tau \end{aligned} \end{aligned}$$

for some constant C>0 only depending on $|\mathcal {O}|$. Collecting (5.4)-(5.8) in (5.3) we arrive at

5.9 $$\begin{aligned}  &   \Vert \nabla \mathcal {N} \xi (t)\Vert ^2_{\boldsymbol{H}} + \int _0^t \Vert \xi (\tau )\Vert _H^2\, {\mathrm d}\tau \le C\left[ \Vert \nabla \mathcal {N} \xi _0\Vert ^2_{\boldsymbol{H}} + \int _0^t \Vert \xi (\tau )\Vert ^2_{V^*} \, {\mathrm d}\tau \right. \nonumber \\    &   \left. + \int _0^t \left( \widehat{\varphi }(\tau ), \, \mathcal {N} \left[ G(\widehat{\varphi }_1(\tau ))-G(\widehat{\varphi }_2(\tau ))\right] \, {\mathrm d}\widehat{W}\right) _H + \varepsilon \int _0^t \Vert \varphi (\tau )\Vert ^2_{H^3(\mathcal {O})} \, {\mathrm d}\tau \right] . \end{aligned}$$

Raising (5.9) to the power $\frac{p}{8}$, taking supremums in time and $\mathbb {P}$-expectations leads to

$$\begin{aligned}  &   \mathop { \mathbb {E}}\sup _{\tau \in [0,\,t]}\Vert \xi (\tau )\Vert ^\frac{p}{4}_{V^*} + \mathop { \mathbb {E}}\left| \int _0^t \Vert \xi (\tau )\Vert ^2_H \, {\mathrm d}\tau \right| ^\frac{p}{8} \le C\left[ \mathop { \mathbb {E}}\Vert \xi _0\Vert ^\frac{p}{4}_{V^*} + \mathop { \mathbb {E}}\left| \int _0^t \Vert \xi (\tau )\Vert ^2_{V^*} \, {\mathrm d}\tau \right| ^\frac{p}{8} \right. \\  &   \left. + \mathop { \mathbb {E}}\sup _{\tau \in [0,\,t]}\left| \int _0^\tau \left( \xi (s), \, \mathcal {N} \left[ G(\varphi _\varepsilon (s))-G(\varphi (s)) \right] \, {\mathrm d}W(s) \right) _H \right| ^\frac{p}{8} + \varepsilon ^\frac{p}{8}\mathop { \mathbb {E}}\left| \int _0^t \Vert \varphi (\tau )\Vert ^2_{H^3(\mathcal {O})} \, {\mathrm d}\tau \right| ^\frac{p}{8}\right] , \end{aligned}$$

where the constant C>0 depends on *p*. The stochastic integral is controlled using the Burkholder–Davis–Gundy inequality, namely

$$\begin{aligned} \begin{aligned}&\mathop { \mathbb {E}}\sup _{\tau \in [0,\,t]}\left| \int _0^\tau \left( \xi (s), \, \mathcal {N} \left[ G(\varphi _\varepsilon (s))-G(\varphi (s)) \right] \, {\mathrm d}W(s) \right) _H \right| ^\frac{p}{8} \\&\hspace{0.3cm} \le C\mathop { \mathbb {E}}\left| \int _0^t \Vert \nabla \mathcal {N}\xi (\tau )\Vert _{\boldsymbol{H}}^2\left\| \nabla \mathcal {N} \left[ G(\varphi _\varepsilon (s))-G(\varphi (s)) \right] \right\| ^2_{\mathscr {L}^2(U, {\boldsymbol{H}})} \, {\mathrm d}\tau \right| ^\frac{p}{16} \\&\hspace{0.3cm} \le C\mathop { \mathbb {E}}\left[ \sup _{\tau \in [0,\,t]}\Vert \nabla \mathcal {N} \xi (\tau )\Vert _{\boldsymbol{H}}^\frac{p}{8} \left| \int _0^t \Vert \xi (\tau )\Vert ^2_{V^*} \, {\mathrm d}\tau \right| ^\frac{p}{16} \right] \\&\hspace{0.3cm} \le \dfrac{1}{4}\mathop { \mathbb {E}}\sup _{\tau \in [0,\,t]}\Vert \xi (\tau )\Vert _{V^*}^{\frac{p}{4}} + C\mathop { \mathbb {E}}\left| \int _0^t \Vert \xi (\tau )\Vert ^2_{V^*} \, {\mathrm d}\tau \right| ^\frac{p}{8}. \end{aligned} \end{aligned}$$

All of the estimates above then imply

5.10 $$\begin{aligned}  &   \mathop { \mathbb {E}}\sup _{\tau \in [0,\,t]}\Vert \xi (\tau )\Vert ^\frac{p}{4}_{V^*} + \mathop { \mathbb {E}}\left| \int _0^t \Vert \xi (\tau )\Vert ^2_H \, {\mathrm d}\tau \right| ^\frac{p}{8} \nonumber \\    &   \le C\left[ \mathop { \mathbb {E}}\Vert \xi _0\Vert ^\frac{p}{4}_{V^*} + \mathop { \mathbb {E}}\left| \int _0^t \Vert \xi (\tau )\Vert ^2_{V^*} \, {\mathrm d}\tau \right| ^\frac{p}{8} + \varepsilon ^\frac{p}{8}\mathop { \mathbb {E}}\left| \int _0^t \Vert \varphi (\tau )\Vert ^2_{H^3(\mathcal {O})} \, {\mathrm d}\tau \right| ^\frac{p}{8}\right] , \end{aligned}$$

for a constant C>0 only depending on the structural parameters of the problem, including *p* and *T*. Therefore, (5.10) and the Gronwall lemma imply that

$$\begin{aligned}&\Vert \widehat{\varphi }\Vert ^\frac{p}{4}_{L^\frac{p}{4}(\Omega ; L^\infty (0,T;V^*))} + \Vert \widehat{\varphi }\Vert ^\frac{p}{4}_{L^\frac{p}{4}(\Omega ; L^2(0,T;H))} \\&\quad \le C\left[ \Vert \xi _0\Vert ^\frac{p}{4}_{L^\frac{p}{4}(\Omega ;V^*)} + \varepsilon ^\frac{p}{8}\mathop { \mathbb {E}}\left| \int _0^t \Vert \varphi (\tau )\Vert ^2_{H^3(\mathcal {O})} \, {\mathrm d}\tau \right| ^\frac{p}{8}\right] \end{aligned}$$

and using the assumption on the initial condition the claim is proven.

## Nonlocal-to-local asymptotics: general regular potentials

Finally, this section is devoted to show Theorem 2.14. To establish the nonlocal-to-local asymptotics for the system in the case of general regular potentials, we devise uniform estimates and perform again a compactness argument. As we shall see, some additional effort is needed to obtain ε-uniform estimates.

### Uniform estimates with respect to ε

In this subsection, we prove that the solutions to (2.4) satisfy uniform estimates independent of ε. The dependence of the estimate for the *H*-norm of $\varphi _\varepsilon$ on the norm of ∇K makes it challenging to retrieve a uniform estimate with respect to ε. Therefore, we resort to a weaker norm, in the same spirit of the computations shown in Section 5. More precisely, we show the following lemma.

#### Lemma 6.1

The estimate

$$\begin{aligned}  &   \mathop { \mathbb {E}}\sup _{\tau \in [0,\,t]}\Vert \varphi _\varepsilon (\tau )\Vert ^p_{V^*} + \mathop { \mathbb {E}}\left| \int _0^t \Lambda _\varepsilon (\varphi _\varepsilon (\tau ),\varphi _\varepsilon (\tau )) \, {\mathrm d}\tau \right| ^\frac{p}{2} \\  &   \le C\left[ \mathop { \mathbb {E}}\Vert \varphi _{0,\varepsilon }\Vert ^p_{V^*} + \mathop { \mathbb {E}}\left| \int _0^t \Vert \varphi _\varepsilon (\tau )\Vert ^2_{V^*} \, {\mathrm d}\tau \right| ^\frac{p}{2} + \mathop { \mathbb {E}}\left| \int _0^t \Vert F(\varphi _\varepsilon (\tau ))\Vert _{L^1(\mathcal {O})}\, {\mathrm d}\tau \right| ^\frac{p}{2}\right] \end{aligned}$$

holds for all p≥2, all ε>0 and for a constant C>0 independent of ε.

#### Proof

We apply the Itô formula to the functional

$$ \Gamma : V^*_0 \rightarrow \mathbb {R}, \qquad \Gamma (u) = \dfrac{1}{2} \Vert \nabla \mathcal {N} u \Vert ^2_{\boldsymbol{H}} $$

evaluated at $u=\varphi _\varepsilon (t)-\overline{\varphi _{0,\varepsilon }}$. This is well defined owing to Assumption (**A4**), and results in

6.1 $$\begin{aligned}&\dfrac{1}{2} \Vert \nabla \mathcal {N} (\varphi _\varepsilon (t)-\overline{\varphi _{0,\varepsilon }})\Vert ^2_{\boldsymbol{H}} + \int _0^t (\varphi _\varepsilon (\tau )-\overline{\varphi _{0,\varepsilon }},\,\mu _\varepsilon (\tau ))_H\, {\mathrm d}\tau \nonumber \\&\quad = \dfrac{1}{2} \Vert \nabla \mathcal {N} (\varphi _{0,\varepsilon }-\overline{\varphi _{0,\varepsilon }})\Vert ^2_{\boldsymbol{H}} + \dfrac{1}{2} \int _0^t \left\| \nabla \mathcal {N} \left[ G(\varphi _\varepsilon (\tau )) \right] \right\| ^2_{\mathscr {L}^2(U, {\boldsymbol{H}})} \, {\mathrm d}\tau \nonumber \\  &\qquad + \int _0^t \left( \varphi _\varepsilon (\tau )-\overline{\varphi _{0,\varepsilon }}, \, \mathcal {N} \left[ G(\varphi _\varepsilon (\tau ))\right] \, {\mathrm d}W \right) _H. \end{aligned}$$

Recalling the definition of $\mu _\varepsilon$, it follows that

$$\begin{aligned}&\int _0^t (\varphi _\varepsilon (\tau )-\overline{\varphi _{0,\varepsilon }},\,\mu _\varepsilon (\tau ))_H\, {\mathrm d}\tau \\&\quad = \int _0^t \big (\varphi _\varepsilon (\tau )-\overline{\varphi _{0,\varepsilon }},\,\mathcal {L}_\varepsilon \varphi _\varepsilon (\tau ) + F^\prime (\varphi _\varepsilon (\tau ))\big )_H\, {\mathrm d}\tau \\&\quad = 2\int _0^t\Lambda _\varepsilon (\varphi _\varepsilon (\tau ),\, \varphi _\varepsilon (\tau )) \, {\mathrm d}\tau + \int _0^t \big (\varphi _\varepsilon (\tau ), F^\prime (\varphi _\varepsilon (\tau ))\big )_H\, {\mathrm d}\tau \\&\qquad - \int _0^t \overline{\varphi _{0,\varepsilon }} \, \overline{F'(\varphi _\varepsilon (\tau ))}|\mathcal {O}| \, {\mathrm d}\tau , \end{aligned}$$

owing to the fact that $\overline{\mathcal {L}_\varepsilon \varphi _\varepsilon } = 0$. Here, we also defined the positive nonlocal energy contribution

$$\begin{aligned}&\Lambda _\varepsilon : H\times H\rightarrow \mathbb {R}^+, \quad \\&\Lambda _\varepsilon (h,k) := \dfrac{1}{2}\int _\mathcal {O}\int _\mathcal {O}K_\varepsilon (x-y)(h(x)-h(y))(k(x)-k(y))\,{\mathrm d}y\, {\mathrm d}x. \end{aligned}$$

In particular, Assumption (**A2**) and Remark 2.1 yield

$$\begin{aligned} \int _0^t \big (\varphi _\varepsilon (\tau ), F^\prime (\varphi _\varepsilon (\tau ))\big )_H\, {\mathrm d}\tau \ge -\alpha \int _0^t \Vert \varphi _\varepsilon (\tau )\Vert _H^2\, {\mathrm d}\tau , \end{aligned}$$

while, proceeding similarly as in (4.5), Assumption (**A4**) gives

$$\begin{aligned} \int _0^t \left\| \nabla \mathcal {N} \left[ G(\varphi _\varepsilon (\tau )) \right] \right\| ^2_{\mathscr {L}^2(U, {\boldsymbol{H}})} \, {\mathrm d}\tau \le C\int _0^t\Vert \varphi _\varepsilon (\tau )\Vert _{V^*}^2{\mathrm d}\tau . \end{aligned}$$

Finally, observe that, for ε sufficiently small,

$$ \begin{aligned} \int _0^t |\overline{\varphi _{0,\varepsilon }}| \, |\overline{F'(\varphi _\varepsilon (\tau ))}||\mathcal {O}|\,{{\mathrm d}\tau }&\le C(1 + |\overline{\varphi _0}|)\left( t+\int _0^t \Vert F(\varphi _\varepsilon (\tau ))\Vert _{L^1(\mathcal {O})}\, {\mathrm d}\tau \right) \\&= C\left( t+\int _0^t \Vert F(\varphi _\varepsilon (\tau ))\Vert _{L^1(\mathcal {O})} \, {\mathrm d}\tau \right) . \end{aligned} $$

In light of all of the above, recalling the embedding $H \hookrightarrow V^*$, (6.1) reads as

6.2 $$\begin{aligned}&\Vert \nabla \mathcal {N} (\varphi _\varepsilon (t)-\overline{\varphi _{0,\varepsilon }})\Vert ^2_{\boldsymbol{H}} + \int _0^t\Lambda _\varepsilon (\varphi _\varepsilon (\tau ), \varphi _\varepsilon (\tau )) \, {\mathrm d}\tau \nonumber \\&\quad \le C\left[ 1+ \Vert \nabla \mathcal {N} (\varphi _{0,\varepsilon }-\overline{\varphi _{0,\varepsilon }})\Vert ^2_{\boldsymbol{H}}+ \int _0^t \Vert \varphi _\varepsilon (\tau )\Vert _H^2\, {\mathrm d}\tau + \int _0^t \Vert F(\varphi _\varepsilon (\tau ))\Vert _{L^1(\mathcal {O})}\, {\mathrm d}\tau \right. \nonumber \\&\qquad \left. + \int _0^t \left( \varphi _\varepsilon (\tau ), \, \mathcal {N} \left[ G(\varphi _\varepsilon (\tau ))\right] \, {\mathrm d}W \right) _H \right] . \end{aligned}$$

One crucial result enabled by the peculiar structure of the nonlocal kernels $K_\varepsilon$ is given by the so-called nonlocal Ehrling inequality (see [9, Lemma 3.4]), enabling to control the second term to the right hand side in terms of $\Lambda _\varepsilon$ and the $V^*$-norm. This then leads from (6.2) to

6.3 $$\begin{aligned}&\Vert \nabla \mathcal {N} (\varphi _\varepsilon (t)-\overline{\varphi _{0,\varepsilon }})\Vert ^2_{\boldsymbol{H}} + \int _0^t\Lambda _\varepsilon (\varphi _\varepsilon (\tau ), \varphi _\varepsilon (\tau )) \, {\mathrm d}\tau \nonumber \\&\quad \le C\left[ 1 + \Vert \nabla \mathcal {N} (\varphi _{0,\varepsilon }-\overline{\varphi _{0,\varepsilon }})\Vert ^2_{\boldsymbol{H}}+ \int _0^t \Vert \varphi _\varepsilon (\tau )\Vert _{V^*}^2\, {\mathrm d}\tau +\int _0^t \Vert F(\varphi _\varepsilon (\tau ))\Vert _{L^1(\mathcal {O})}\, {\mathrm d}\tau \right. \nonumber \\  &\qquad \left. + \int _0^t \left( \varphi _\varepsilon (\tau ), \, \mathcal {N} \left[ G(\varphi _\varepsilon (\tau ))\right] \, {\mathrm d}W \right) _H \right] . \end{aligned}$$

Summing to both sides of (6.3) the average $|\overline{\varphi _{0,\varepsilon }}|^2$, then raising the result to the power $\tfrac{p}{2}$, taking supremums in time and $\mathbb {P}$-expectations, yields

$$\begin{aligned}&\mathop { \mathbb {E}}\sup _{\tau \in [0,\,t]}\Vert \varphi _\varepsilon (\tau )\Vert ^p_{V^*} + \mathop { \mathbb {E}}\left| \int _0^t \Lambda _\varepsilon (\varphi _\varepsilon (\tau ),\varphi _\varepsilon (\tau )) \, {\mathrm d}\tau \right| ^\frac{p}{2} \\&\quad \le C\left[ \mathop { \mathbb {E}}\Vert \varphi _{0,\varepsilon }\Vert ^p_{V^*} + \mathop { \mathbb {E}}\left| \int _0^t \Vert \varphi _\varepsilon (\tau )\Vert ^2_{V^*} \, {\mathrm d}\tau \right| ^\frac{p}{2} + \mathop { \mathbb {E}}\left| \int _0^t \Vert F(\varphi _\varepsilon (\tau ))\Vert _{L^1(\mathcal {O})}\, {\mathrm d}\tau \right| ^\frac{p}{2}\right. \\&\qquad \left. + \mathop { \mathbb {E}}\sup _{\tau \in [0,\,t]}\left| \int _0^\tau \left( \varphi _\varepsilon (s), \, \mathcal {N} \left[ G(\varphi _\varepsilon (s))\right] \, {\mathrm d}W(s) \right) _H \right| ^\frac{p}{2} \right] , \end{aligned}$$

where the constant C>0 depends on *p* but is independent of ε. Exploiting the Burkholder-Davis-Gundy inequality as in the previous section, it holds

$$\begin{aligned} \mathop { \mathbb {E}}\sup _{\tau \in [0,\,t]}\left| \int _0^\tau \left( \varphi _\varepsilon (s), \, \mathcal {N} \left[ G(\varphi _\varepsilon (s))\right] \, {\mathrm d}W(s) \right) _H \right| ^\frac{p}{2} \le \dfrac{1}{2}\mathop { \mathbb {E}}\sup _{\tau \in [0,\,t]}\Vert \varphi _\varepsilon (\tau )\Vert _{V^*}^p \\ + C\mathop { \mathbb {E}}\left| \int _0^t \Vert \varphi _\varepsilon (\tau )\Vert ^2_{V^*} \, {\mathrm d}\tau \right| ^\frac{p}{2}. \end{aligned}$$

Altogether, we conclude

$$\begin{aligned}  &   \mathop { \mathbb {E}}\sup _{\tau \in [0,\,t]}\Vert \varphi _\varepsilon (\tau )\Vert ^p_{V^*} + \mathop { \mathbb {E}}\left| \int _0^t \Lambda _\varepsilon (\varphi _\varepsilon (\tau ),\varphi _\varepsilon (\tau )) \, {\mathrm d}\tau \right| ^\frac{p}{2} \\  &   \quad \le C\left[ \mathop { \mathbb {E}}\Vert \varphi _{0,\varepsilon }\Vert ^p_{V^*} + \mathop { \mathbb {E}}\left| \int _0^t \Vert \varphi _\varepsilon (\tau )\Vert ^2_{V^*} \, {\mathrm d}\tau \right| ^\frac{p}{2} + \mathop { \mathbb {E}}\left| \int _0^t \Vert F(\varphi _\varepsilon (\tau ))\Vert _{L^1(\mathcal {O})}\, {\mathrm d}\tau \right| ^\frac{p}{2}\right] \end{aligned}$$

where the constant C>0 is independent of ε. □

In order to be able to close the estimate in Lemma 6.1, we need to refine the energy estimate. Precisely, we show the following result.

#### Lemma 6.2

It holds that

(i) the family $\{\varphi _\varepsilon \}_{\varepsilon > 0}$ is uniformly bounded in $L^p(\Omega ;L^\infty (0,T;V^*))$;
(ii) the family $\{\Lambda _\varepsilon (\varphi _\varepsilon ,\varphi _\varepsilon )\}_{\varepsilon > 0}$ is uniformly bounded in $L^\frac{p}{2}(\Omega ;L^\infty (0,T))$;
(iii) the family $\{\mu _\varepsilon \}_{\varepsilon >0}$ is uniformly bounded in $L^\frac{p}{2}(\Omega ;L^2(0,T;V))$;
(iv) the family $\{\nabla \mu _\varepsilon \}_{\varepsilon >0}$ is uniformly bounded in $L^p(\Omega ;L^2(0,T;{\boldsymbol{H}}))$;
(v) the family $\{\Psi (\varphi _\varepsilon )\}_{\varepsilon >0}$ is uniformly bounded in $L^\frac{p}{2}(\Omega ;L^\infty (0,T;L^1(\mathcal {O})))$.

#### Proof

We apply the Itô formula to the full nonlocal energy functional

$$\begin{aligned} \mathcal {E}_\varepsilon : H\rightarrow \mathbb {R},\qquad \mathcal {E}_\varepsilon (u) := \frac{1}{4}\int _\mathcal {O}\int _\mathcal {O}K_\varepsilon (x-y)|u(x) - u(y)|^2 \, {\mathrm d}y \, {\mathrm d}x + \int _\mathcal {O}F(u(x)) \, {\mathrm d}x \end{aligned}$$

evaluated at $u=\varphi _\varepsilon (t)$. This yields

6.4 $$\begin{aligned}  &   \mathcal {E}_\varepsilon (\varphi _\varepsilon (t)) + \int _0^t\int _\mathcal {O}|\nabla \mu _{\varepsilon }(\tau )|^2\,{\mathrm d}x\,{\mathrm d}\tau = \mathcal {E}_\varepsilon (\varphi _{0, \varepsilon }) + \int _0^t\Big (\mu _{\varepsilon }(s),G(\varphi _\varepsilon (s))\,{\mathrm d}W(s)\Big )_H \nonumber \\  &   \qquad \quad + \frac{1}{2}\int _0^t\sum _{k=0}^{+\infty }\left[ \frac{1}{2}\Lambda _\varepsilon (G(\varphi _\varepsilon (\tau ))[u_k], G(\varphi _\varepsilon (\tau ))[u_k]) +\int _\mathcal {O}F^{\prime \prime }(\varphi _\varepsilon (\tau ))|G(\varphi _\varepsilon (\tau ))[u_k]|^2\,{\mathrm d}x \right] \,{\mathrm d}\tau . \end{aligned}$$

By means of the nonlocal Poincaré (see [35, Theorem 1.1]) and Ehrling inequalities, as well as Assumption (**A7**), we can control the third term on the right hand side as

$$\begin{aligned}&\int _0^t\sum _{k=0}^{+\infty }\Lambda _\varepsilon (G(\varphi _\varepsilon (\tau ))[u_k], G(\varphi _\varepsilon (\tau ))[u_k])\,{\mathrm d}\tau \\&\le C\int _0^t\sum _{k=0}^{+\infty }\Vert G(\varphi _\varepsilon (\tau ))[u_k]\Vert _V^2\,{\mathrm d}\tau \\&=C\int _0^t\Vert G(\varphi _\varepsilon (\tau ))\Vert _{\mathscr {L}^2(U,V)}^2\,{\mathrm d}\tau \\&\le C\left( 1+ \int _0^t\Vert \varphi _\varepsilon (\tau )\Vert _H^2\,{\mathrm d}\tau \right) \\&\le C\left( 1+\int _0^t \Lambda _\varepsilon (\varphi _\varepsilon (\tau ), \varphi _\varepsilon (\tau )) \, {\mathrm d}\tau + \int _0^t\Vert \varphi _\varepsilon (\tau )\Vert _{V^*}^2\,{\mathrm d}\tau \right) . \end{aligned}$$

As, by assumption, the initial energies $\mathcal {E}_\varepsilon (\varphi _{0,\varepsilon })$ are suitably uniformly bounded, taking into account the above inequality in (6.4), raising to the power $\tfrac{p}{2}$ and taking the supremum in time as well as $\mathbb {P}$-expectations, we have

$$\begin{aligned}&\mathop { \mathbb {E}}\sup _{\tau \in [0,\,t]}|\Lambda _\varepsilon (\varphi _\varepsilon (t), \varphi _\varepsilon (\tau ))|^\frac{p}{2} + \mathop { \mathbb {E}}\sup _{\tau \in [0,\,t]}\Vert F(\varphi _\varepsilon (t))\Vert _{L^1(\mathcal {O})}^{\tfrac{p}{2}} + \mathop { \mathbb {E}}\left| \int _0^t\Vert \nabla \mu _\varepsilon (\tau )\Vert _{\boldsymbol{H}}^2\,{\mathrm d}\tau \right| ^{\tfrac{p}{2}} \\&\quad \le C\Bigg (1+\mathop { \mathbb {E}}\left| \int _0^t \Lambda _\varepsilon (\varphi _\varepsilon (\tau ), \varphi _\varepsilon (\tau )) \, {\mathrm d}\tau \right| ^\frac{p}{2} +\mathop { \mathbb {E}}\left| \int _0^t\Vert \varphi _\varepsilon (\tau )\Vert _{V^*}^2\,{\mathrm d}\tau \right| ^\frac{p}{2} \\&\qquad +\mathop { \mathbb {E}}\left| \int _0^t\Big (\mu _{\varepsilon }(s),G(\varphi _\varepsilon (s))\,{\mathrm d}W(s)\Big )_H\right| ^{\tfrac{p}{2}} + \mathop { \mathbb {E}}\left| \int _0^t\sum _{k=0}^{+\infty }\int _\mathcal {O}F^{\prime \prime }(\varphi _\varepsilon (\tau ))|G(\varphi _\varepsilon (\tau )[u_k]|^2\,{\mathrm d}x \,{\mathrm d}\tau \right| ^{\tfrac{p}{2}}\Bigg ). \end{aligned}$$

Using the Burkholder-Davis-Gundy inequality (as in (3.18)), we can bound the stochastic integral on the right-hand side and get an additional term involving the mean of $\mu _\varepsilon$. For the remaining term, we invoke Assumptions (**A2**)-(ii), (**A3**)-(ii) and Hölder’s inequality to get

$$ \begin{aligned}&\mathop { \mathbb {E}}\left| \int _0^t\sum _{k=0}^{+\infty }\int _\mathcal {O}|F^{\prime \prime }(\varphi _{\varepsilon }(\tau ))||G(\varphi _{\varepsilon }(\tau )[u_k]|^2\,{\mathrm d}x \, {\mathrm d}s\right| ^{\frac{p}{2}} \\&\hspace{1cm}\le C\left[ 1 + \mathop { \mathbb {E}}\left| \int _0^t\sum _{k=0}^{+\infty }\int _\mathcal {O}|F(\varphi _{\varepsilon }(\tau ))||G(\varphi _{\varepsilon }(\tau )[u_k]|^2\,{\mathrm d}x \, {\mathrm d}s\right| ^{\frac{p}{2}} \right] \\&\hspace{1cm}\le C\left[ 1 + \mathop { \mathbb {E}}\left| \int _0^t\Vert F(\varphi _{\varepsilon }(\tau ))\Vert _{L^1(\mathcal {O})}\sum _{k=0}^{+\infty } \Vert G(\varphi _{\varepsilon }(\tau )[u_k]\Vert _{L^\infty (\mathcal {O})}^2 \, {\mathrm d}s\right| ^{\frac{p}{2}} \right] \\&\hspace{1cm}\le C\left[ 1 + \mathop { \mathbb {E}}\left| \int _0^t\Vert F(\varphi _{\varepsilon }(\tau ))\Vert _{L^1(\mathcal {O})} \, {\mathrm d}s\right| ^{\frac{p}{2}} \right] . \end{aligned} $$

Finally, it is left to control the mean value of $\mu _\varepsilon$. Testing the equation for the chemical potential by 1 and recalling that $\overline{\mathcal {L}_\varepsilon \varphi _\varepsilon }=0$, we arrive at

$$ \begin{aligned} \left| |\mathcal {O}|\,\overline{\mu _{\varepsilon }} \right|&= \left| (\mu _{\varepsilon }, 1)_H \right| \le |(\mathcal {L}_\varepsilon \varphi _{\varepsilon }, 1)_H| + |(F^\prime (\varphi _{\varepsilon }), 1)_H| \\&\le C\left( 1 + \Vert F(\varphi _{\varepsilon })\Vert _{L^1(\mathcal {O})}\right) , \end{aligned} $$

implying

6.5 $$\begin{aligned} \mathop { \mathbb {E}}\sup _{\tau \in [0,\,t]}|\overline{\mu _\varepsilon (\tau )}|^\frac{p}{2} \le C\left( 1 + \mathop { \mathbb {E}}\sup _{\tau \in [0,\,t]}\Vert F(\varphi _\varepsilon (\tau ))\Vert _{L^1(\mathcal {O})}^\frac{p}{2} \right) . \end{aligned}$$

Eventually, collecting all the terms above, we arrive at

6.6 $$\begin{aligned}  &   \mathop { \mathbb {E}}\sup _{\tau \in [0,\,t]}|\Lambda _\varepsilon (\varphi _\varepsilon (\tau ), \varphi _\varepsilon (\tau ))|^\frac{p}{2}+\mathop { \mathbb {E}}\sup _{\tau \in [0,\,t]}\Vert F(\varphi _\varepsilon (\tau ))\Vert _{L^1(\mathcal {O})}^{\frac{p}{2}} + \mathop { \mathbb {E}}\left| \int _0^t\Vert \nabla \mu _\varepsilon (\tau )\Vert _H^2\,{\mathrm d}\tau \right| ^{\frac{p}{2}} \\  &   \le C\Bigg (1+\mathop { \mathbb {E}}\left| \int _0^t \Lambda _\varepsilon (\varphi _\varepsilon (\tau ), \varphi _\varepsilon (\tau )) \, {\mathrm d}\tau \right| ^\frac{p}{2}+ \mathop { \mathbb {E}}\left| \int _0^t\Vert \varphi _\varepsilon (\tau )\Vert _{V^*}^2\,{\mathrm d}\tau \right| ^\frac{p}{2} +\mathop { \mathbb {E}}\left| \int _0^t \Vert F(\varphi _\varepsilon (\tau ))\Vert _{L^1(\mathcal {O})} \, {\mathrm d}\tau \right| ^\frac{p}{2}\Bigg ). \end{aligned}$$

Summing the estimate in Lemma 6.1, (6.66.7) as well as (6.5) multiplied by a sufficiently small constant yields

6.7 $$\begin{aligned}&\mathop { \mathbb {E}}\sup _{\tau \in [0,\,t]}\Vert \varphi _\varepsilon (\tau )\Vert ^p_{V^*} + \mathop { \mathbb {E}}\sup _{\tau \in [0,\,t]}|\Lambda _\varepsilon (\varphi _\varepsilon (\tau ), \varphi _\varepsilon (\tau ))|^\frac{p}{2}+\mathop { \mathbb {E}}\sup _{\tau \in [0,\,t]}\Vert F(\varphi _\varepsilon (\tau ))\Vert _{L^1(\mathcal {O})}^{\frac{p}{2}} \nonumber \\&\qquad + \mathop { \mathbb {E}}\sup _{\tau \in [0,\,t]}|\overline{\mu _\varepsilon (\tau )}|^\frac{p}{2}+\mathop { \mathbb {E}}\left| \int _0^t\Vert \nabla \mu _\varepsilon (\tau )\Vert _H^2\,{\mathrm d}\tau \right| ^{\frac{p}{2}} \nonumber \\&\quad \le C\left[ 1 +\mathop { \mathbb {E}}\Vert \varphi _{0,\varepsilon }\Vert ^p_{V^*} + \mathop { \mathbb {E}}\left| \int _0^t \Vert \varphi _\varepsilon (\tau )\Vert ^2_{V^*} \, {\mathrm d}\tau \right| ^\frac{p}{2} + \mathop { \mathbb {E}}\left| \int _0^t \Vert F(\varphi _\varepsilon (\tau ))\Vert _{L^1(\mathcal {O})}\, {\mathrm d}\tau \right| ^\frac{p}{2}\right. \nonumber \\&\quad \left. + \mathop { \mathbb {E}}\left| \int _0^t \Lambda _\varepsilon (\varphi _\varepsilon (\tau ), \varphi _\varepsilon (\tau )) \, {\mathrm d}\tau \right| ^\frac{p}{2}\right] \end{aligned}$$

and the Gronwall lemma gives the estimates

$$\begin{aligned} \Vert \varphi _\varepsilon \Vert _{L^p(\Omega ;L^\infty (0,T;V^*))}+\Vert \Lambda _\varepsilon (\varphi _\varepsilon , \varphi _\varepsilon )\Vert _{L^\frac{p}{2}(\Omega ;L^\infty (0,T))}&\le C,\\ \Vert \mu _{\varepsilon }\Vert _{L^\frac{p}{2}(\Omega ;L^2(0,T;V))} + \Vert \nabla \mu _{\varepsilon }\Vert _{L^p(\Omega ;L^2(0,T;{\boldsymbol{H}}))}+\Vert \Psi (\varphi _\varepsilon )\Vert _{L^\frac{p}{2}(\Omega ;L^\infty (0,T;L^1(\mathcal {O})))}&\le C, \end{aligned}$$

and the proof is complete. □

For brevity, we collect the next three estimates in the following result.

#### Lemma 6.3

It holds that

(i) the family $\{\varphi _\varepsilon \}_{\varepsilon >0}$ is uniformly bounded in $L^p(\Omega ;L^\infty (0,T;H))$ for all p≥2;
(ii) the family $\{G(\varphi _\varepsilon )\}_{\varepsilon >0}$ is uniformly bounded in
  $$ L^\infty (\Omega \times (0,T);\mathscr {L}^2(U,H))\cap L^p(\Omega ;L^\infty (0,T;\mathscr {L}^2(U,V))) $$
  for all p≥2;
(iii) the family $\left\{ \displaystyle \int _0^. G(\varphi _{\varepsilon }(s)) \, {\mathrm d}W(s)\right\} _{\varepsilon >0}$ is uniformly bounded in
  $$ L^q(\Omega ;W^{k,q}(0,T;H))\cap L^p(\Omega ;W^{k,p}(0,T;V)) $$
  for all p≥2, q≥2 and $k \in (0, \frac{1}{2})$.

#### Proof

Once again, the nonlocal Ehrling inequality proves to be a crucial result to carry out the nonlocal-to-local asymptotics. Indeed, for ε sufficiently small, it holds

$$\begin{aligned} \Vert \varphi _\varepsilon (t)\Vert _H^2 \le \delta \Lambda _\varepsilon (\varphi _\varepsilon (t),\varphi _\varepsilon (t)) + C_\delta \Vert \varphi _\varepsilon (t)\Vert _{V^*}^2 \end{aligned}$$

for some fixed δ>0, and therefore

$$ \Vert \varphi _\varepsilon \Vert _{L^p(\Omega ;L^\infty (0,T;H))} \le C\left( \Vert \Lambda _\varepsilon (\varphi _\varepsilon ,\varphi _\varepsilon )\Vert _{L^\frac{p}{2}(\Omega ;L^\infty (0,T))} + \Vert \varphi _\varepsilon \Vert _{L^p(\Omega ;L^\infty (0,T;V^*))} \right) . $$

Owing to Lemma 6.2, we then conclude that

6.8 $$\begin{aligned} \Vert \varphi _\varepsilon \Vert _{L^p(\Omega ;L^\infty (0,T;H))}\le C \end{aligned}$$

for a constant C>0 independent of ε. Arguing as in Subsection 3.2, exploiting [20, Lemma 2.1] jointly with Assumptions (**A3**) and (**A7**), we get

$$\begin{aligned} \Vert G(\varphi _\varepsilon )\Vert _{L^\infty (\Omega \times (0,T);\mathscr {L}^2(U,H))\cap L^p(\Omega ;L^\infty (0,T;\mathscr {L}^2(U,V)))}&\le C, \\ \left\| \int _0^. G(\varphi _{\varepsilon }(s)) \, {\mathrm d}W(s)\right\| _{L^q(\Omega ;W^{k,q}(0,T;H))\cap L^p(\Omega ;W^{k,p}(0,T;V))}&\le C \end{aligned}$$

for every $k\in (0,\frac{1}{2})$ and q≥2. Similarly as in the proof of Lemma 3.8, it follows that

$$\begin{aligned} \Vert \varphi _{\varepsilon }\Vert _{L^p(\Omega ;W^{\alpha ,p}(0,T;V^*))} \le C \end{aligned}$$

for some $\alpha = \alpha (p) > \frac{1}{p}$ if p>2. □

Finally, we show the last estimate.

#### Lemma 6.4

The families $\{F^\prime (\varphi _\varepsilon )\}_{\varepsilon >0}$ and $\{\mathcal {L}_\varepsilon \varphi _\varepsilon \}_{\varepsilon >0}$ are uniformly bounded in $L^{\frac{p}{2}}(\Omega ;L^2(0,T;H))$.

#### Proof

Using Assumption (**A2**) and recalling Remark 2.1, we can multiply the equation for $\mu _\varepsilon$ by $F^\prime (\varphi _\varepsilon )+\gamma \varphi _\varepsilon$, where $\gamma > \alpha$. Hence, we get

$$\begin{aligned} \int _\mathcal {O}\mu _\varepsilon F^\prime (\varphi _\varepsilon )\;{\mathrm d}x + \gamma \int _\mathcal {O}\varphi _\varepsilon \mu _\varepsilon \;{\mathrm d}x&= \int _\mathcal {O}\mathcal {L}_\varepsilon \varphi _\varepsilon (F^\prime (\varphi _\varepsilon )+\gamma \varphi _\varepsilon )\;{\mathrm d}x \\&\quad +\int _\mathcal {O}|F^\prime (\varphi _\varepsilon )|^2\;{\mathrm d}x + \int _\mathcal {O}\varphi _\varepsilon F'(\varphi _\varepsilon ) \, {\mathrm d}x. \end{aligned}$$

Letting $\Psi '(s):= F^\prime (s)+\gamma s$ for any $s \in \mathbb {R}$, we observe that it is monotone by construction. Therefore, since the kernel $K_\varepsilon$ is nonnegative, we have

$$\begin{aligned}&\int _\mathcal {O}\mathcal {L}_\varepsilon \varphi _\varepsilon \Psi ^\prime (\varphi _\varepsilon )\;{\mathrm d}x \\&\quad = \frac{1}{2}\int _\mathcal {O}\int _\mathcal {O}K_\varepsilon (x-y)(\varphi _\varepsilon (x)-\varphi _\varepsilon (y))(\Psi ^\prime (\varphi _\varepsilon (x))-\Psi ^\prime (\varphi _\varepsilon (y)))\;{\mathrm d}y\;{\mathrm d}x \ge 0. \end{aligned}$$

Eventually, invoking Young‘s inequality twice, it follows that

$$\begin{aligned} \Vert F^\prime (\varphi _\varepsilon )\Vert _{H}^2 \le C(\Vert \mu _\varepsilon \Vert _H^2 + \Vert \varphi _\varepsilon \Vert _H^2) \end{aligned}$$

and therefore, owing to the proven estimates,

$$\begin{aligned} \Vert F^\prime (\varphi _\varepsilon )\Vert _{L^\frac{p}{2}(\Omega ;L^2(0,T;H))} \le C, \end{aligned}$$

where the constant *C* does not depend on ε. As a byproduct, by comparison in the equation for $\mu _\varepsilon$, we obtain

$$\begin{aligned} \Vert \mathcal {L}_\varepsilon \varphi _\varepsilon \Vert _{L^\frac{p}{2}(\Omega ;L^2(0,T;H))} \le C, \end{aligned}$$

and the proof is complete. □

### Passage to the limit as $\varepsilon \rightarrow 0^+$

In this section, we pass to the limit as $\varepsilon \rightarrow 0^+$ in the nonlocal problems (2.4), exploiting the previously shown uniform estimates. Accordingly, we tacitly fix a decreasing vanishing sequence $\{\varepsilon _n\}_{n \in \mathbb {N}}$ without relabeling. In particular, following the same logic of Subsections 3.3 and 3.6, we investigate the tightness properties of the sequence of approximate solutions. Once again, we recall that the embedding

$$\begin{aligned} L^\infty (0,T;H) \cap W^{\alpha , p}(0,T;V^*)&\hookrightarrow C^0([0,T]; H^{-\delta }(\mathcal {O})) \end{aligned}$$

is compact whenever αp>1 for some small δ>0. To recover compactness in $L^2(0,T;H)$, a different route must be followed, as a uniform estimate in $L^p(\Omega ;L^2(0,T;V))$ is typically out of reach, due to the singular behavior of $\nabla K_\varepsilon$ in the limit $\varepsilon \rightarrow 0^+$. Here, we shall proceed a posteriori. The following result holds: its proof is entirely analogous to the one of Lemma 3.9 and is therefore omitted.

#### Lemma 6.5

If p>2, then the family of laws of $\{\varphi _{\varepsilon }\}_{\varepsilon >0}$ is tight in the space $C^0([0,T]; H^{-\delta }(\mathcal {O}))$ for any $\delta \in (0, \frac{1}{2})$.

Similarly, setting

$$\begin{aligned} G(\varphi _{\varepsilon })\cdot W := \int _0^. G(\varphi _{\varepsilon }(s))\,{\mathrm d}W(s), \end{aligned}$$

we immediately have the counterpart of Lemma 3.10, i.e.,

#### Lemma 6.6

The family of laws of $\{G(\varphi _\varepsilon ) \cdot W\}_{\varepsilon >0}$ is tight in the space $C^0([0,T]; V^*)$.

It even holds a stronger statement, due to Assumption (**A7**): the sequence is even tight in $C^0([0,T];H)$ if p>2. Nonetheless, Lemma 6.6 is sufficient for our purposes. As for the last two tightness lemmas, arguing similarly to Lemmas 3.11 and 3.12, i.e., identifying the Wiener process *W* with a constant sequence of random variables, we first have

#### Lemma 6.7

The family of laws of $\{W_\varepsilon \}_{\varepsilon >0}$ is tight in the space $C^0([0,T]; U_0)$.

Then, for the initial conditions, given that $\varphi _{0,\varepsilon } \rightarrow \varphi _0$ in $L^p(\Omega ;H)$, we have

#### Lemma 6.8

The family of laws of $\{\varphi _{0,\varepsilon }\}_{\varepsilon >0}$ is tight in $V^*$.

Therefore, by Lemmas 6.5-6.8, the family of laws of

$$\begin{aligned} \{\varphi _{\varepsilon },\, G(\varphi _{\varepsilon })\cdot W,\, W_\varepsilon ,\, \varphi _{0,\varepsilon }\}_{\varepsilon >0} \end{aligned}$$

is tight in the space

$$\begin{aligned} C^0([0,T];H^{-\delta }(\mathcal {O})) \times C^0([0,T]; V^*) \times C^0([0,T];U_0) \times V^*. \end{aligned}$$

In order to pass to the limit, we can exploit the approach established by Gyöngy and Krylov in [30, Lemma 1.1], by showing convergence in probability. Fix two arbitrary subsequences of $\{\varphi _\varepsilon \}_{\varepsilon >0}$, that we denote with

$$ \{\varphi _{\varepsilon _j}\}_{j \in \mathbb {N}}, \qquad \{\varphi _{\varepsilon _k}\}_{k \in \mathbb {N}}, $$

having values in the Polish space $\mathcal {X}:= C^0([0,T];H^{-\delta }(\mathcal {O}))$. The sequence of joint laws of $(\varphi _{\varepsilon _j}, \varphi _{\varepsilon _k})$ is clearly tight in $\mathcal {X}^2$ by Lemma 6.5. Therefore, by the Prokhorov theorem, the sequence is relatively compact in the space of probability measures over $\mathcal {X}^2$ (and its Borel σ-algebra), i.e., it admits a weakly convergent subsequence, namely

$$ \operatorname {Law}_\mathbb {P}(\varphi _{\varepsilon _{j_i}}, \varphi _{\varepsilon _{k_i}}) \rightharpoonup \varsigma $$

for suitable choices of $j_i$ and $k_i$ as $i \in \mathbb {N}$ and for some limit probability measure ς. Using the Skorokhod theorem, as in the proof of existence of martingale solutions, we can frame this convergence in a new probability space $(\Omega ', \mathscr {F}', \mathbb {P}')$ through law-preserving maps $Z_i:(\Omega ', \mathscr {F}')\rightarrow (\Omega , \mathscr {F})$ (i.e., such that $\mathbb {P}' \circ Z_i^{-1} = \mathbb {P}$ for all $i \in \mathbb {N}$). In particular, we obtain

$$ (\varphi _{\varepsilon _{j_i}}', \varphi _{\varepsilon _{k_i}}') :=(\varphi _{\varepsilon _{j_i}}, \varphi _{\varepsilon _{k_i}}) \circ Z_i \rightarrow (\varphi _1,\varphi _2) \qquad \text {in }\mathcal {X}^2, \quad \mathbb {P}\text {-a.s.} $$

for some limit process

$$ (\varphi _1,\varphi _2): (\Omega ', \mathscr {F}') \rightarrow (\mathcal {X}^2, \mathscr {B}(\mathcal {X}^2)). $$

By the same token, the remaining tightness results yield the convergences

$$\begin{aligned} (I'_{\varepsilon _{j_i}}, I'_{\varepsilon _{k_i}}) := (G(\varphi _{\varepsilon _{j_i}}) \cdot W,\, G(\varphi _{\varepsilon _{k_i}}) \cdot W) \circ Z_i \rightarrow (I_1, I_2)&\qquad \text {in }\mathcal {X}^2, \quad \mathbb {P}\text {-a.s.} \\ (W'_{\varepsilon _{j_i}}, W'_{\varepsilon _{k_i}}) := (W_{\varepsilon _{j_i}}, W_{\varepsilon _{k_i}}) \circ Z_i \rightarrow (W_1, W_2)&\qquad \text {in } [C^0([0,T];U_0]^2, \quad \mathbb {P}\text {-a.s.}\\ (\varphi _{0, \varepsilon _{j_i}}', \varphi _{0, \varepsilon _{k_i}}') := (\varphi _{0, \varepsilon _{j_i}}, \varphi _{0, \varepsilon _{k_i}}) \circ Z_i \rightarrow (\varphi _{01}, \varphi _{02})&\qquad \text {in }V^* \times V^*, \quad \mathbb {P}\text {-a.s.}\qquad \end{aligned}$$

where the limit processes belong to the specified spaces. Since the sequence of Wiener processes is constant and given the known convergence of the initial conditions, as laws are preserved it is immediate to identify

$$ W_1 = W_2 = W, \qquad \varphi _{01}=\varphi _{02}= \varphi _0. $$

On account of the uniform estimates proven in the previous subsection, by reflexivity and compactness we can establish the following convergences:

$$\begin{aligned} (\varphi _{\varepsilon _{j_i}}', \varphi _{\varepsilon _{k_i}}') \rightarrow (\varphi _1, \varphi _2)&\qquad \text { in } L^q(\Omega ', \mathcal {X}^2) \quad \forall \, q < p, \\ (\varphi _{\varepsilon _{j_i}}', \varphi _{\varepsilon _{k_i}}') \overset{*}{\rightharpoonup }(\varphi _1, \varphi _2)&\qquad \text { in }L^p_w({\Omega }'; L^\infty (0,T;H)) \cap L^p({\Omega }';W^{\alpha , p}(0,T;V^*)),\\ (\mu _{\varepsilon _{j_i}}', \mu _{\varepsilon _{k_i}}') := (\mu _{\varepsilon _{j_i}}, \mu _{\varepsilon _{k_i}})\circ Z_i \rightharpoonup \ {(\mu _1, \mu _2)}&\qquad \text { in }L^{\frac{p}{2}}({\Omega }';L^2(0,T;V)). \\ (F'(\varphi _{\varepsilon _{j_i}}'), F'(\varphi _{\varepsilon _{k_i}}')) \rightharpoonup {(\eta _1,\eta _2)}&\qquad \text { in }L^{\frac{p}{2}}({\Omega }';L^2(0,T;H)). \\ (\mathcal {L}_{\varepsilon _{j_i}}(\varphi _{\varepsilon _{j_i}}'), \mathcal {L}_{\varepsilon _{k_i}}(\varphi _{\varepsilon _{k_i}}')) \rightharpoonup {(\xi _1,\xi _2)}&\qquad \text { in }L^{\frac{p}{2}}({\Omega }';L^2(0,T;H)). \end{aligned}$$

Fix then any two sufficiently large values ℓ and $m \in \mathbb {N}$. Exploiting the nonlocal version of Ehrling‘s inequality, for any ρ>0 there exists $C_\rho >0$ such that

$$\begin{aligned}&\mathbb {E}^{\prime }\Vert \varphi _{\varepsilon _{j_\ell }}' - \varphi _{\varepsilon _{j_m}}'\Vert _{L^\infty (0,T;H)}^q\\&\le \rho {\mathbb {E}}^{\prime }\Vert \Lambda _{\varepsilon _{j_\ell }}(\varphi _{\varepsilon _{j_\ell }}') + \Lambda _{{j_m}}(\varphi _{\varepsilon _{j_m}}')\Vert _{L^\infty (0,T)}^\frac{q}{2} + C_\rho {\mathbb {E}}^{\prime }\Vert \varphi _{\varepsilon _{j_\ell }}' - \varphi _{\varepsilon _{j_m}}'\Vert _{C^0(0,T;V^*)}^q \\&\le C\rho + C_\rho {\mathbb {E}}^{\prime }\Vert \varphi _{\varepsilon _{j_\ell }}' - \varphi _{\varepsilon _{j_m}}'\Vert _{C^0(0,T;V^*)}^q, \end{aligned}$$

where q<p and we exploited the second uniform estimate in Lemma 6.2. Given the strong convergence in $\mathcal {X}$ and that ρ>0 can be chosen arbitrarily small, this yields that $\{\varphi _{\varepsilon _{j_i}}'\}_{i\in \mathbb {N}}$ is a Cauchy sequence in $L^q({\Omega }';L^\infty (0,T;H))$, and therefore

6.9 $$\begin{aligned} (\varphi _{\varepsilon _{j_i}}', \varphi _{\varepsilon _{k_i}}') \rightarrow (\varphi _1, \varphi _2)&\quad \text { in }L^q({\Omega }';[L^\infty (0,T;H)]^2) \quad \forall \, q < p. \end{aligned}$$

As a byproduct, recalling Assumption (**A3**) on *G*, we derive that

$$\begin{aligned} \Vert G(\varphi _{\varepsilon _{j_i}}') - G(\varphi _1)\Vert _{L^q({\Omega }';L^\infty (0,T;\mathscr {L}^2(U,H)))} \rightarrow 0 \end{aligned}$$

as $i\rightarrow +\infty$, and therefore,

$$\begin{aligned} (G(\varphi _{\varepsilon _{j_i}}'), G(\varphi _{\varepsilon _{k_i}}')) \rightarrow (G(\varphi _1),G(\varphi _2)) \quad \text { in }L^q(\Omega ';[L^\infty (0,T;\mathscr {L}^2(U,H))]^2) \quad \forall \, q <p. \end{aligned}$$

for all q<p. Next, setting as usual $\Psi '(s) = F'(s) + \gamma s$ for some $\gamma > \alpha$ and all $s \in \mathbb {R}$, let us show that $\eta _m = \Psi ^\prime (\varphi _m) -\gamma \varphi _m = F^\prime (\varphi _m)$ for $m \in \{1,2\}$. Using the strong convergence (6.10), by comparison we infer that

$$\begin{aligned} (\Psi ^\prime (\varphi _{\varepsilon _{j_i}}'), \Psi ^\prime (\varphi _{\varepsilon _{k_i}}')) \rightharpoonup (\eta _1 + \gamma \varphi _1, \eta _2+\gamma \varphi _2) \quad \text { in }L^{\frac{p}{2}}(\Omega ';[L^2(0,T;H)]^2). \end{aligned}$$

The identification is then a consequence of the weak-strong closure of maximal monotone operators. Finally, we show that $\varphi _m\in L^p({\Omega }';L^\infty (0,T;V))\cap L^{\frac{p}{2}}({\Omega }';L^2(0,T;H^2(\mathcal {O})))$ and $\xi _m = -\Delta \varphi _m$ for $m \in \{1,2\}$. For instance, since (6.10) holds in $L^q(\Omega ' \times (0,T); H)$ as well, it holds that

$$ \varphi _{\varepsilon _{j_i}}'(t) \rightarrow \varphi _1(t) \quad \text {in }H $$

in the $\mathbb {P}' \otimes {\mathrm d}t$-almost sure sense, up to a non-relabeled subsequence. We can exploit pathwise [9, Lemma 3.3] to get

$$\begin{aligned} \Lambda _0(\varphi _1(t)) \le \liminf _{i\rightarrow +\infty } \Lambda _\varepsilon (\varphi _{\varepsilon _{j_i}}'(t), \varphi _{\varepsilon _{j_i}}'(t)), \end{aligned}$$

holding $\mathbb {P}' \otimes {\mathrm d}t$-almost surely, where we set

$$\begin{aligned} \Lambda _0(u) := \frac{1}{2}\int _\mathcal {O}|\nabla u(x)|^2\;{\mathrm d}x\qquad \forall \,u\in V. \end{aligned}$$

Eventually, taking powers $\frac{p}{2}$, supremums in time and $\mathbb {P}'$-expectations yields, owing also to Fatou’s lemma,

$$ {\mathbb {E}}^{\prime }\sup _{t \in [0,T]}|\Lambda _0(\varphi _1(t))|^\frac{p}{2} \le \liminf _{i\rightarrow +\infty } {\mathbb {E}}^{\prime }\sup _{t \in [0,T]}|\Lambda _\varepsilon (\varphi _{\varepsilon _{j_i}}'(t), \varphi _{\varepsilon _{j_i}}'(t)|^\frac{p}{2} \le C, $$

ensuring that $\varphi _1\in L^p({\Omega }';L^\infty (0,T;V))$. Furthermore, due to [9, Lemma 3.2], it holds

$$\begin{aligned}  &   \int _0^T\Lambda _{\varepsilon _{j_i}}(\varphi _{ji}'(\tau ), \varphi _{ji}'(\tau ))\;{\mathrm d}\tau + \int _0^T\int _\mathcal {O}((K_{\varepsilon _{j_i}}*1)\varphi _{\varepsilon _{j_i}}' - K_\varepsilon *\varphi _{\varepsilon _{j_i}}')(\tau ,x)(\zeta -\varphi _{\varepsilon _{j_i}}')(\tau ,x)\;{\mathrm d}x\,{\mathrm d}\tau \\    &   \quad \quad \quad \quad \quad \quad \quad \quad \quad \quad \quad \le \int _0^T\Lambda _{\varepsilon _{j_i}}(\zeta (\tau ),\zeta (\tau ))\;{\mathrm d}\tau \end{aligned}$$

for all $\zeta \in L^2(0,T;V)$. On account of (6.10), [9, Lemma 3.3] as well as Fatou‘s lemma, it follows that

$$\begin{aligned}&\frac{1}{2}\int _0^T\int _\mathcal {O}|\nabla \varphi _1(\tau ,x)|^2{\mathrm d}x\,{\mathrm d}\tau + \int _0^T\int _\mathcal {O}\xi _1(\tau ,x)(\zeta -\varphi _1)(\tau ,x)\;{\mathrm d}x\,{\mathrm d}\tau \\&\quad \le \frac{1}{2}\int _0^T\int _\mathcal {O}|\nabla \zeta (\tau ,x)|^2\;{\mathrm d}x\,{\mathrm d}\tau \end{aligned}$$

for all $\zeta \in L^2(0,T;V)$, $\mathbb {P}'$-almost surely. Indeed, this qualifies $\xi _1$ as an element of the subdifferential $\partial \widetilde{\Lambda }_0$ at the point $u=\varphi _1$, where

$$ \widetilde{\Lambda }_0(u) = \dfrac{1}{2}\int _0^T\int _\mathcal {O}|\nabla u(x,t)|^2 \, {\mathrm d}x \, {\mathrm d}t \qquad \forall \, u \in L^2(0,T;V), $$

extended with value +∞ to the whole $L^2(0,T;H)$. Therefore, we have

$$\begin{aligned} \int _0^T\int _\mathcal {O}\xi _1(x, t)\zeta (x, t)\;{\mathrm d}x \, {\mathrm d}t = \int _0^T\int _\mathcal {O}\nabla \varphi _1(x,t)\cdot \nabla \zeta (x,t)\;{\mathrm d}x\, {\mathrm d}t\qquad \forall \, \zeta \in L^2(0,T;V), \end{aligned}$$

and, in turn,

$$\begin{aligned} \int _\mathcal {O}\xi _1(x, t)\zeta (x)\;{\mathrm d}x = \int _\mathcal {O}\nabla \varphi _1(x, t)\cdot \nabla \zeta (x)\;{\mathrm d}x\qquad \forall \, \zeta \in V. \end{aligned}$$

Thus, by elliptic regularity theory, we conclude that $\varphi _1\in L^2(0,T;H^2(\mathcal {O})))$ $\mathbb {P}'$-almost surely and that $\xi _1(t) = -\Delta \varphi _1(t)$ for almost all times and $\mathbb {P}'$-almost surely, as well as $\partial _{\boldsymbol{n}}\varphi _1=0$ almost everywhere on $\partial \mathcal {O}\times (0,T)$, and $\mathbb {P}'$-almost surely. Furthermore, given

$$ \Vert \varphi _1\Vert _{H^2(\mathcal {O})}^2 \le C\left( \Vert \varphi _1\Vert ^2_H + \Vert \Delta \varphi _1\Vert _H^2 \right) $$

we eventually deduce that $\varphi _1 \in L^\frac{p}{2}({\Omega }', L^2(0,T;H^2(\mathcal {O}))$, matching the regularity provided in [36]. The proven convergences are now enough to identify the limit of the problem. After carrying out a procedure analogous to the one illustrated in Subsection 3.4 to identify the stochastic integrals, it is straightforward to show that the limit processes $(\varphi _1, \varphi _2)$ satisfy the local Cahn–Hilliard system

$$ {\left\{ \begin{array}{ll} {\mathrm d}\varphi _m -\Delta \mu _m \, {\mathrm d}t = G(\varphi _m) \, {\mathrm d}W &  \quad \text {in } \mathcal {O}\times (0,T), \\ \mu _m = -\Delta \varphi _m + F'(\varphi _m)&  \quad \text {in } \mathcal {O}\times (0,T), \\ \partial _{\boldsymbol{n}}\mu _m = \partial _{\boldsymbol{n}}\varphi _m = 0 &  \quad \text {on } \partial \mathcal {O}\times (0,T), \\ \varphi _m(0) = \varphi _0 &  \quad \text {in } \mathcal {O}, \end{array}\right. } $$

where $m \in \{1,2\}$. The pathwise uniqueness result given in [37] for the system, however, yields immediately that $\varphi _1 = \varphi _2$, $\mathbb {P}'$-almost surely. Thanks to preservation of laws, therefore, the limit law ς must be supported on the diagonal of $\mathcal {X}^2$, i.e.,

$$ \varsigma ((v_1, v_2) \in \mathcal {X}^2: v_1 = v_2) = 1. $$

Then, [30, Lemma 1.1] yields convergence in probability with respect to the topology of $\mathcal {X}$ for the original sequence $\varphi _\varepsilon$, on the original probability space $(\Omega , \mathscr {F}, \mathbb {P})$. Iterating precisely the previous argument on the sequence $\{\varphi _\varepsilon \}_{\varepsilon > 0}$ finally completes the proof of Theorem 2.14.

## Data Availability

No datasets were generated or analysed during the current study.
